# Diretriz da Sociedade Brasileira de Cardiologia sobre a Análise e Emissão de Laudos Eletrocardiográficos – 2022

**DOI:** 10.36660/abc.20220623

**Published:** 2022-09-08

**Authors:** Nelson Samesima, Epotamenides Good God, Jose Claudio Lupi Kruse, Marcelo Garcia Leal, Claudio Pinho, Francisco Faustino de A. C. França, João Pimenta, Acácio Fernandes Cardoso, Adail Paixão, Alfredo Fonseca, Andrés R. Pérez-Riera, Antonio Luiz Pinho Ribeiro, Bruna Affonso Madaloso, Bráulio Luna, Carlos Alberto Rodrigues de Oliveira, César José Grupi, Dalmo Antonio Ribeiro Moreira, Elisabeth Kaiser, Gabriela Miana de Mattos Paixão, Gilson Feitosa, Horacio Gomes Pereira, José Grindler, José Luiz Aziz, Marcos Sleiman Molina, Mirella Facin, Nancy M. M. de Oliveira Tobias, Patricia Alves de Oliveira, Paulo César R. Sanches, Ricardo Alkmin Teixeira, Severiano Melo Atanes, Carlos Alberto Pastore

**Affiliations:** 1 Hospital das Clínicas Faculdade de Medicina Universidade de São Paulo São Paulo SP Brasil Instituto do Coração (InCor) do Hospital das Clínicas da Faculdade de Medicina da Universidade de São Paulo (HCFMUSP), São Paulo , SP – Brasil; 2 Hospital SOCOR Belo Horizonte MG Brasil Hospital SOCOR , Belo Horizonte , MG – Brasil; 3 Instituto de Cardiologia do Rio Grande do Sul Porto Alegre RS Brasil Instituto de Cardiologia do Rio Grande do Sul , Porto Alegre , RS – Brasil; 4 Hospital das Clínicas de Ribeirão Preto Ribeirão Preto SP Brasil Hospital das Clínicas de Ribeirão Preto , Ribeirão Preto , SP – Brasil; 5 Pontifícia Universidade Católida Campinas SP Brasil Pontifícia Universidade Católida (PUC), Campinas , SP – Brasil; 6 Clínica Pinho Valinhos SP Brasil Clínica Pinho , Valinhos , SP – Brasil; 7 Instituto Dante Pazzanese de Cardiologia São Paulo SP Brasil Instituto Dante Pazzanese de Cardiologia , São Paulo , SP – Brasil; 8 Hospital do Servidor Público Estadual São Paulo SP Brasil Hospital do Servidor Público Estadual , São Paulo , SP – Brasil; 9 Hospital das Clínicas Faculdade de Medicina Universidade de São Paulo São Paulo SP Brasil Serviço de Eletrocardiologia do Hospital das Clínicas da Faculdade de Medicina da Universidade de São Paulo (HCFMUSP), São Paulo , SP – Brasil; 10 Hospital Unimec Vitória Da Conquista BA Brasil Hospital Unimec , Vitória Da Conquista , BA – Brasil; 11 Hospital das Clínicas Faculdade de Medicina Universidade de São Paulo São Paulo SP Brasil Hospital das Clínicas da Faculdade de Medicina da Universidade de São Paulo (HCFMUSP), São Paulo , SP – Brasil; 12 Faculdade de Medicina do ABC Santo André SP Brasil Faculdade de Medicina do ABC , Santo André , SP – Brasil; 13 Universidade Federal de Minas Gerais Belo Horizonte MG Brasil Universidade Federal de Minas Gerais (UFMG), Belo Horizonte , MG – Brasil; 14 Hospital São Paulo Universidade Federal de São Paulo São Paulo SP Brasil Hospital São Paulo , Universidade Federal de São Paulo (UNIFESP), São Paulo , SP – Brasil; 15 Grupo Fleury São Paulo SP Brasil Grupo Fleury , São Paulo , SP – Brasil; 16 Hospital das Clínicas Universidade Federal de Minas Gerais Belo Horizonte MG Brasil Hospital das Clínicas da Universidade Federal de Minas Gerais (UFMG), Belo Horizonte , MG – Brasil; 17 Hospital Santa Izabel Santa Casa da Bahia Salvador BA Brasil Hospital Santa Izabel , Santa Casa da Bahia , Salvador , BA – Brasil; 18 Clínica São Paulo São Paulo SP Brasil Clínica São Paulo , São Paulo , SP – Brasil; 19 Clínica Médica de Cardiologia Dr. Paulo Sanches Campinas SP Brasil Clínica Médica de Cardiologia Dr. Paulo Sanches , Campinas , SP – Brasil; 20 Hospital Renascentista Pouso Alegre MG Brasil Hospital Renascentista , Pouso Alegre , MG – Brasil; 21 Faculdade de Medicina Universidade do Vale do Sapucaí Pouso Alegre MG Brasil Faculdade de Medicina da Universidade do Vale do Sapucaí (UNIVÁS), Pouso Alegre , MG – Brasil; 22 Policlínica Maceió SS São Paulo SP Brasil Policlínica Maceió SS , São Paulo , SP – Brasil

**Table:** 

Diretriz da Sociedade Brasileira de Cardiologia sobre a Análise e Emissão de Laudos Eletrocardiográficos – 2022	
O relatório abaixo lista as declarações de interesse conforme relatadas à SBC pelos especialistas durante o período de desenvolvimento deste posicionamento, 2021.	
Especialista	Tipo de relacionamento com a indústria
Acácio Fernandes Cardoso	Nada a ser declarado
Adail Paixao Almeida	Nada a ser declarado
Alfredo José da Fonseca	Nada a ser declarado
Andrés R. Pérez-Riera	Nada a ser declarado
Antonio Luiz Pinho Ribeiro	Outros relacionamentos Atuação no último ano como auditor médico para empresa operadora de planos de saúde ou assemelhada: - Consultoria em Inteligência Artificial em Saúde para Unimed-BH Participação em órgãos governamentais de regulação, ou de defesa de direitos na área de cardiologia: - Atuação junto ao Ministério da Saúde em Convênios em saúde digital e apoio a Atenção Primária à Saúde
Braulio Luna Filho	Nada a ser declarado
Bruna Affonso Madaloso	Declaração financeira A - Pagamento de qualquer espécie e desde que economicamente apreciável, feito a (i) você, (ii) ao seu cônjuge/companheiro ou a qualquer outro membro que resida com você, (iii) a qualquer pessoa jurídica em que qualquer destes seja controlador, sócio, acionista ou participante, de forma direta ou indireta, recebimento por palestras, aulas, atuação como proctor de treinamentos, remunerações, honorários pagos por participações em conselhos consultivos, de investigadores, ou outros comitês, etc. provenientes da indústria farmacêutica, de órteses, próteses, equipamentos e implantes, brasileiras ou estrangeiras. - SBC - Curso de Eletrocardiografia Básica Pesquisa Clínica; empresa Immune BioSolutions Inc.
Carlos Alberto Pastore	Nada a ser declarado
Carlos Alberto Rodrigues de Oliveira	Nada a ser declarado
Cesar José Grupi	Nada a ser declarado
Claudio Pinho	Outros relacionamentos Financiamento de atividades de educação médica continuada, incluindo viagens, hospedagens e inscrições para congressos e cursos, provenientes da indústria farmacêutica, de órteses, próteses, equipamentos e implantes,brasileiras ou estrangeiras. - Bayer: Xarelto
Dalmo Antônio Ribeiro Moreira	Outros relacionamentos Financiamento de atividades de educação médica continuada, incluindo viagens, hospedagens e inscrições para congressos e cursos, provenientes da indústria farmacêutica, de órteses, próteses, equipamentos e implantes,brasileiras ou estrangeiras. - Bayer, Abbott, Libbs, Astra Zeneca, Daichy Sankio
Elisabeth Kaiser	Nada a ser declarado
Epotamenides Maria Good God	Nada a ser declarado
Francisco Faustino de Albuquerque Carneiro de França	Nada a ser declarado
Gabriela Miana de Mattos Paixão	Nada a ser declarado
Gilson Soares Feitosa Filho	Nada a ser declarado
Horacio Gomes Pereira Filho	Nada a ser declarado
João A Pimenta de Almeida	Nada a ser declarado
Jose Claudio Lupi Kruse	Nada a ser declarado
José Grindler	Nada a ser declarado
Jose Luis Aziz	Declaração financeira A - Pagamento de qualquer espécie e desde que economicamente apreciável, feito a (i) você, (ii) ao seu cônjuge/companheiro ou a qualquer outro membro que resida com você, (iii) a qualquer pessoa jurídica em que qualquer destes seja controlador, sócio, acionista ou participante, de forma direta ou indireta, recebimento por palestras, aulas, atuação como proctor de treinamentos, remunerações, honorários pagos por participações em conselhos consultivos, de investigadores, ou outros comitês, etc. provenientes da indústria farmacêutica, de órteses, próteses, equipamentos e implantes, brasileiras ou estrangeiras. - Astrazeca: hipertensão e diabetes; Daiichi Sankyo: hipertensão e fibrilação atrial
Marcelo Garcia Leal	Nada a ser declarado
Marcos Sleiman Molina	Outros relacionamentos Participação societária de qualquer natureza e qualquer valor economicamente apreciável de empresas na área de saúde, de ensino ou em empresas concorrentes ou fornecedoras da SBC: - Proprietário de clínica privada na cidade de Mogi das Cruzes
Mirella Facin	Nada a ser declarado
Nancy Maria Martins De Oliveira	Nada a ser declarado
Nelson Samesima	Nada a ser declarado
Patricia Alves de Oliveira	Nada a ser declarado
Paulo César Ribeiro Sanches	Nada a ser declarado
Ricardo Alkmim Teixeira	Declaração financeira A - Pagamento de qualquer espécie e desde que economicamente apreciável, feito a (i) você, (ii) ao seu cônjuge/companheiro ou a qualquer outro membro que resida com você, (iii) a qualquer pessoa jurídica em que qualquer destes seja controlador, sócio, acionista ou participante, de forma direta ou indireta, recebimento por palestras, aulas, atuação como proctor de treinamentos, remunerações, honorários pagos por participações em conselhos consultivos, de investigadores, ou outros comitês, etc. provenientes da indústria farmacêutica, de órteses, próteses, equipamentos e implantes, brasileiras ou estrangeiras. - Daichii-Sankyo: Lixiana; Boehringer-Ingelheim: Pradaxa, Jardiance; Biotronik/ Abbott/ Medtronic: dispositivos cardíacos eletrônicos implantáveis Outros relacionamentos Financiamento de atividades de educação médica continuada, incluindo viagens, hospedagens e inscrições para congressos e cursos, provenientes da indústria farmacêutica, de órteses, próteses, equipamentos e implantes, brasileiras ou estrangeiras. - Biomedical: bainhas de extração a laser
Severiano Atanes Netto	Nada a ser declarado

## Sumário

Introdução

1. Normatização para Análise e Emissão do

Laudo Eletrocardiográfico

1.1. Normatização para Análise Eletrocardiográfica

1.2. O Laudo Eletrocardiográfico

**1.2.1. Laudo Descritivo**

**1.2.2. Laudo Conclusivo**

**1.2.3. Laudo Automatizado**

**1.2.4. Laudo Via Internet**

2. Avaliação da Qualidade Técnica do Traçado

2.1. Critérios de Avaliação Técnica dos Traçados

**2.1.1. Calibração do Eletrocardiógrafo**

**2.1.2. Troca de Eletrodos**

**2.1.2.1. Posicionamento Trocado dos Eletrodos**

**2.1.2.1.1. Eletrodos dos MMSS Trocados entre Si**

**2.1.2.2. Eletrodo dos MMII trocado por um eletrodo de um dos MMSS**

**2.1.2.3. Troca de Eletrodos entre Braço Esquerdo e Perna Esquerda**

**2.1.2.4. Troca de Eletrodos Precordiais**

**2.1.2.5. Eletrodos V1 e V2 Mal Posicionados**

**2.1.3. Outras Interferências**

**2.1.3.1. Tremores Musculares**

**2.1.3.2. Neuroestimulação**

**2.1.3.3. Frio, Febre, Soluços, Agitação Psicomotora**

**2.1.3.4. “Grande Eletrodo” Precordial**

**2.1.3.5. Oscilação da Linha de Base**

**2.1.3.6. Outras Interferências Elétricas e Eletromagnéticas**

**2.1.3.7. Alterações Decorrentes de Funcionamento Inadequado de**

**Softwares e Sistemas de Aquisição de Sinais Eletrocardiográficos**

**Computadorizados**

3. A Análise do Ritmo Cardíaco

3.1. Análise da Onda P, Frequência Cardíaca e Ritmo

**3.1.1. Definição do Ritmo Sinusal (RS)**

**3.1.2. Frequência da Onda P Sinusal**

3.2. Análise das Alterações de Ritmo Supraventricular

**3.2.1. Definição de Arritmia Cardíaca**

**3.2.2. Arritmia Supraventricular**

**3.2.3. Presença de Onda P Sinusal**

**3.2.3.1. Arritmia Sinusal (AS)**

**3.2.3.2. Bradicardia Sinusal (BS)**

**3.2.3.3. Bloqueio Sinoatrial de Segundo Grau**

**3.2.3.4. Bloqueios Interatriais (BIA)**

**3.2.3.5. Taquicardia Sinusal (TS)**

**3.2.4. Ausência de Onda P Antes do QRS**

**3.2.4.1. Fibrilação Atrial (FA)**

**3.2.4.2. Flutter Atrial**

**3.2.4.3. Ritmo Juncional**

**3.2.4.4. Extrassístole Juncional**

**3.2.4.5. Taquicardia por Reentrada Nodal Comum (TRN)**

**3.2.4.6. Taquicardia por Reentrada Atrioventricular Ortodrômica (TRAV)**

**3.2.5. Presença da Onda P Não Sinusal Antes do QRS**

**3.2.5.1. Ritmo Atrial Ectópico (RAE)**

**3.2.5.2. Ritmo Atrial Multifocal (RAM)**

**3.2.5.3. Ritmo Juncional**

**3.2.5.4. Batimento de Escape Atrial**

**3.2.5.5. Extrassístole Atrial (EA)**

**3.2.5.6. Extrassístole Atrial Bloqueada ou Não Conduzida**

**3.2.5.7. Taquicardia Atrial (TA)**

**3.2.5.8. Taquicardia Atrial Multifocal (TAMF)**

**3.2.5.9. Taquicardia por Reentrada Nodal Incomum**

**3.2.5.10. Taquicardia de Coumel**

**3.2.6. Pausas**

**3.2.6.1. Parada Sinusal (PS)**

**3.2.6.2. Disfunção do Nó Sinusal (DNS)**

**3.2.7. Classificação de Taquicardias Supraventriculares Baseadas no**

**Intervalo RP**

**3.2.8. Arritmias Supraventriculares com Complexo QRS Alargado**

**3.2.8.1. Aberrância de Condução**

**3.2.8.2. Extrassístole Atrial com Aberrância de Condução**

**3.2.8.3. Taquicardia Supraventricular com Aberrância de Condução**

**3.2.8.4. Taquicardia por Reentrada Atrioventricular Antidrômica**

4. Condução Atrioventricular

4.1. Definição da Relação Atrioventricular (AV) Normal

**4.1.1. Atraso da Condução Atrioventricular (AV)**

**4.1.1.1. Bloqueio AV de Primeiro Grau**

**4.1.1.2. Bloqueio AV de Segundo Grau Tipo I (Mobitz I)**

**4.1.1.3. Bloqueio AV de Segundo Grau Tipo II (Mobitz II)**

**4.1.1.4. Bloqueio AV 2:1**

**4.1.1.5. Bloqueio AV Avançado ou de Alto Grau**

**4.1.1.6. Bloqueio AV do Terceiro Grau ou BAV Total (BAVT)**

**4.1.1.7. Bloqueio AV Paroxístico**

**4.1.2. Pré-Excitação Ventricular**

**4.1.3. Outros Mecanismos de Alteração da Relação AV Normal**

**4.1.3.1. Dissociação AV**

**4.1.3.2. Ativação Atrial Retrógrada**

5. Análise da Ativação Ventricular

5.1. Ativação Ventricular Normal

**5.1.1. Definição do QRS Normal**

**5.1.2. Eixo Elétrico Normal no Plano Frontal**

**5.1.3. Ativação Ventricular Normal no Plano Horizontal**

**5.1.4. Análise das Alterações de Ritmo Ventricular**

**5.1.4.1. Definição de Arritmia Cardíaca**

**5.1.4.2. Arritmia Ventricular**

**5.1.4.3. Análise das Arritmias Ventriculares**

**5.1.4.3.1. Extrassístole Ventricular (EV)**

**5.1.4.3.2. Batimento(s) de Escape Ventricular(es)**

**5.1.4.3.3. Ritmo de Escape Ventricular – Ritmo Idioventricular**

**5.1.4.3.4. Ritmo Idioventricular Acelerado (RIVA)**

**5.1.4.3.5. Taquicardia Ventricular (TV)**

**5.1.4.3.5.1. Taquicardia Ventricular Monomórfica**

**5.1.4.3.5.2. Taquicardia Ventricular Polimórfica (TVP)**

**5.1.4.3.5.3. Taquicardia Ventricular Tipo Torsade des Pointes (TdP)**

**5.1.4.3.5.4. Taquicardia Ventricular Bidirecional**

**5.1.4.3.5.5. Quanto à Duração**

**5.1.4.3.6. Batimento de Fusão**

**5.1.4.3.7. Batimento com Captura Supraventricular Durante**

**Ritmo Idioventricular**

**5.1.4.3.8. Parassístole Ventricular (PV)**

**5.1.4.3.9. Fibrilação Ventricular (FV)**

**5.1.4.4. Critérios de Diferenciação entre as Taquicardias de**

**Complexo QRS Alargado**

6. Sobrecargas das Câmaras Cardíacas

6.1. Sobrecargas Atriais

**6.1.1. Sobrecarga Atrial Esquerda (SAE)**

**6.1.2. Sobrecarga Atrial Direita (SAD)**

**6.1.3. Sobrecarga Biatrial (SBA)**

**6.1.4. Sobrecarga Ventricular Esquerda (SVE)**

**6.1.4.1. Critérios de Romhilt-Estes**

**6.1.4.2. Índice de Sokolow Lyon**

**6.1.4.3. Índice de Cornell**

**6.1.4.4. Peguero-Lo Presti**

**6.1.4.5. Alterações de Repolarização Ventricular**

**6.1.5. Sobrecarga Ventricular Direita (SVD)**

**6.1.5.1. Eixo do QRS**

**6.1.5.2. Onda R Ampla**

**6.1.5.3. Morfologia qR ou qRs**

**6.1.5.4. Morfologia rsR’**

**6.1.5.5. Repolarização Ventricular**

**6.1.5.6. Critério de SEATTLE para SVD**

**6.1.6. Sobrecarga Biventricular**

**6.1.7. Diagnóstico Diferencial do Aumento de Amplitude do QRS**

7. Análise dos Bloqueios (Retardo, Atraso de Condução)

Intraventriculares

7.1. Bloqueios Intraventriculares

**7.1.1. Bloqueio do Ramo Esquerdo (BRE)**

**7.1.1.1. Bloqueio de Ramo Esquerdo em Associação com Sobrecarga**

**Ventricular Esquerda**

**7.1.1.2. Bloqueio de Ramo Esquerdo em Associação com Sobrecarga**

**Ventricular Direita (ao Menos 2 dos 3 Critérios)**

**7.1.2. Bloqueio do Ramo Direito (BRD)**

**7.1.2.1. Atraso Final de Condução**

**7.1.3. Bloqueios Divisionais do Ramo Esquerdo**

**7.1.3.1 Bloqueio Divisional Anterossuperior Esquerdo (BDAS)**

**7.1.3.2. Bloqueio Divisional Anteromedial Esquerdo (BDAM)**

**7.1.3.3. Bloqueio Divisional Posteroinferior Esquerdo (BDPI)**

**7.1.4. Bloqueios Divisionais do Ramo Direito**

**7.1.4.1. Bloqueio Divisional Superior Direito (BDSRD)**

**7.1.4.2. Bloqueio Divisional Inferior Direito (BDIRD)**

**7.1.5. Associação de Bloqueios**

**7.1.5.1. BRE Associado ao BDAS**

**7.1.5.2. BRE Associado ao BDPI**

**7.1.5.3. BRD Associado ao BDAS**

**7.1.5.4. BRD Associado ao BDPI**

**7.1.5.5. BRD Associado ao BDAS e BDAM**

**7.1.5.6. BDAS Associado ao BDAM**

**7.1.5.7. Bloqueio de Ramo Mascarado**

**7.1.6. Situações Especiais Envolvendo a Condução Intraventricular**

**7.1.6.1. Bloqueio Peri-infarto**

**7.1.6.2. Bloqueio Peri-isquemia**

**7.1.6.3. Fragmentação do QRS (fQRS)**

**7.1.6.4. Bloqueio de Ramo Esquerdo Atípico**

**7.1.6.5. Bloqueio Intraventricular Parietal ou Purkinje/Músculo ou Focal**

8. Análise do ECG nas Coronariopatias

8.1. Critérios Diagnósticos da Presença de Isquemia Miocárdica

**8.1.1. Presença de Isquemia**

**8.1.2. Isquemia Circunferencial ou Global**

**8.1.3. Alterações Secundárias**

8.2. Critérios Diagnósticos da Presença de Lesão

8.3. Definição das Áreas Eletricamente Inativas (AEI)

8.4. Análise Topográfica da Isquemia, Lesão e Necrose

**8.4.1. Análise Topográfica das Manifestações Isquêmicas ao ECG (Meyers)**

**8.4.2. Análise topográfica das manifestações isquêmicas pelo ECG em**

**associação à ressonância magnética**

**8.4.3. Correlação Eletrocardiográfica com a Artéria Envolvida**

8.5. Infartos de Localização Especial

**8.5.1. Infarto do Miocárdio de Ventrículo Direito**

**8.5.2. Infarto Atrial**

8.6. Diagnósticos Diferenciais

**8.6.1. Isquemia Subepicárdica**

**8.6.2. Infarto Agudo do Miocárdio (IAM) com Supra de ST**

8.7. Associação de Infarto com Bloqueios de Ramo

**8.7.1. Infarto de Miocárdio na Presença de Bloqueio de**

**Ramo Direito (BRD)**

**8.7.2. Infarto do Miocárdio na Presença de Bloqueio de**

**Ramo Esquerdo (BRE)**

9. Análise da Repolarização Ventricular

9.1. Repolarização Ventricular

**9.1.1. Repolarização Ventricular Normal**

**9.1.1.1. Ponto J**

**9.1.1.2. Segmento ST**

**9.1.1.3. Onda T**

**9.1.1.4. Onda U**

**9.1.1.5. Intervalo QT (QT) e Intervalo QT Corrigido (QTc)**

**9.1.2. Variantes da Repolarização Ventricular Normal**

**9.1.2.1. Padrão de Repolarização Precoce (RP)**

10. O ECG nas Canalopatias e Demais Alterações

Genéticas

10.1. A Genética e o ECG

**10.1.1. Canalopatias**

**10.1.1.1. Síndrome do QT Longo Congênito**

**10.1.1.2. Síndrome do QT Curto**

**10.1.1.3. Síndrome de Brugada**

**10.1.1.4. Taquicardia Catecolaminérgica**

**10.1.2. Doenças Genéticas com Acometimento Primário Cardíaco**

**10.1.2.1. Cardiomiopatia (Displasia) Arritmogênica de**

**Ventrículo Direito**

**10.1.2.2. Cardiomiopatia Hipertrófica**

**10.1.3. Doenças Genéticas com Acometimento Secundário Cardíaco**

**10.1.3.1. Distrofia Muscular**

11. Caracterização das Alterações Eletrocardiográficas

em Situações Clínicas Específicas

11.1. Condições Clínicas que Alteram o ECG

**11.1.1. Ação Digitálica**

**11.1.2. Alterações de ST-T por Fármacos**

**11.1.3. Alternância Elétrica**

**11.1.4. Alternância da Onda T**

**11.1.5. Comprometimento Agudo do Sistema Nervoso Central**

**11.1.6. Comunicação Interatrial (CIA)**

**11.1.7. COVID-19**

**11.1.8. Derrame Pericárdico**

**11.1.9. Dextrocardia**

**11.1.10. Dextroposição**

**11.1.11. Distúrbios Eletrolíticos**

**11.1.11.1. Hiperpotassemia**

**11.1.11.2. Hipopotassemia**

**11.1.11.3. Hipocalcemia**

**11.1.11.4. Hipercalcemia**

**11.1.12. Doença Pulmonar Obstrutiva Crônica (DPOC)**

**11.1.13. Drogas Antiarrítmicas**

**11.1.13.1. Amiodarona**

**11.1.13.2. Propafenona**

**11.1.13.3. Sotalol**

**11.1.14. Efeito Dielétrico**

**11.1.15. Embolia Pulmonar**

**11.1.16. Fenômeno de Ashman (ou de Gounaux-Ashman)**

**11.1.17. Hipotermia**

**11.1.18. Hipotireoidismo**

**11.1.19. Insuficiência Renal Crônica**

**11.1.20. Pericardite**

**11.1.21. Quimioterápicos**

12. O ECG em Atletas

12.1. A Importância do ECG do Atleta

**12.1.1. Achados Eletrocardiográficos Normais (Grupo 1)**

**12.1.2. Achados Eletrocardiográficos Anormais (Grupo 2)**

**12.1.3. Achados Eletrocardiográficos Limítrofes (Grupo 3)**

13. O ECG em Crianças

13.1. Introdução

13.2. Aspectos Técnicos

13.3.Parâmetros Eletrocardiográficos e suas Variações

**13.3.1. Frequência Cardíaca e Ritmo Sinusal**

**13.3.1.1. Possíveis Alterações**

**13.3.1.1.1. Arritmia Sinusal**

**13.3.1.1.2. Taquicardia Sinusal**

**13.3.1.1.3. Bradicardia Sinusal**

**13.3.1.1.4. Outras Bradicardias**

**13.3.2. A onda P e a Atividade Elétrica Atrial**

**13.3.2.1. Possíveis Alterações**

**13.3.2.1.1. Sobrecargas Atriais**

**13.3.2.1.2. Ritmo Juncional**

**13.3.3. Intervalo PR e a Condução Atrioventricular**

**13.3.3.1. Possíveis Alterações**

**13.3.3.1.1. Bloqueios Atrioventriculares**

**13.3.3.1.2. Intervalo PR curto e Pré-excitação Ventricular**

**13.3.4. Atividade Elétrica Ventricular**

**13.3.4.1. Possíveis Alterações**

**13.3.4.1.1. Alterações do Eixo e da Amplitude do QRS**

**13.3.4.1.2. Alterações das Ondas Q**

**13.3.4.1.3. Distúrbios da Condução Intraventricular**

**13.3.4.1.4. Onda Épsilon e a Cardiomiopatia Arritmogênica do**

**Ventrículo Direito**

**13.3.5. Repolarização Ventricular**

**13.3.5.1. Intervalo QT**

**13.3.5.1.1. Possíveis Alterações**

**13.3.5.1.1.1. Síndrome do QT Longo**

**13.3.5.1.1.2. Síndrome do QT Curto**

**13.3.5.2. Segmento ST**

**13.3.5.2.1.1. Desnivelamentos do Segmento ST**

**13.3.5.2.1.2. Repolarização Precoce**

**13.3.5.2.1.3. Padrão eletrocardiográfico de Brugada**

**13.3.5.3. Onda T**

**13.3.5.4. Onda U**

13.4. Distúrbios do Ritmo Cardíaco

13.5. Reconhecimento do Situs, da Posição Cardíaca e da

Inversão Ventricular

14. O ECG durante Estimulação Cardíaca Artificial

14.1. Estimulação Cardíaca Artificial (ECA)

**14.1.1. Termos Básicos**

**14.1.2. Análise das Características Eletrocardiográficas dos DCEI**

15. Tele-eletrocardiografia

Referências

## Introdução

A revisão das diretrizes de eletrocardiografia deve-se ao fato do surgimento de avanços no entendimento de diversas doenças, com repercussões importantes no traçado eletrocardiográfico. Alguns podem imaginar que a interpretação do eletrocardiograma (ECG) não teve mudanças ao longo do tempo; certamente esquecem as doenças recentemente descritas e outras cujos mecanismos eletrofisiológicos foram melhor entendidos na atualidade. Alguns parâmetros eletrocardiográficos são considerados importantes marcadores prognósticos na doença de Chagas, além de ser possível identificar alterações consideradas como preditores de mortalidade na população geral (idade ao ECG - ECG-age). Uma questão crucial é: quando indicar a realização de um ECG?

O ECG é um exame simples, barato e não invasivo. Permite uma ideia da condição cardíaca do indivíduo e pode eventualmente identificar situações de risco de morte súbita. Assim, o achado de um ECG dentro dos limites da normalidade permite antecipar que a função ventricular deve estar normal ou próxima disto, fato importante no primeiro contato com o paciente.

Achamos que todas as pessoas deveriam ter um ECG em algum momento da vida, que somente fosse repetido segundo necessidade clínica. Algumas diretrizes colocam indicação IIb para a realização do ECG em indivíduos assintomáticos da população geral, e classe IIa na presença de hipertensão e ou diabetes. ^1^

A possibilidade de transmissão dos exames através da internet permitiu a difusão da tecnologia por diversas regiões carentes do nosso país e um melhor padrão de atendimento assistencial. Nos últimos anos, observou-se um aumento significativo de estudos (com milhões de ECG’s analisados) sobre inteligência artificial e sistemas de interpretação automática como ferramentas adicionais para a eletrocardiografia. Alguns resultados conseguiram demonstrar a capacidade destes novos sistemas em identificar determinadas arritmas, bem como predizer seu aparecimento, além de desfechos como acidente vascular encefálico (isquêmico).

Assim, esperamos que esta versão ajude o médico clínico e/ou cardiologista na emissão dos laudos eletrocardiográficos de maneira uniforme, permitindo fácil entendimento e padronização da linguagem.

## 1. Normatização para Análise e Emissão do Laudo Eletrocardiográfico

### 1.1. Normatização para Análise Eletrocardiográfica

Para a correta interpretação eletrocardiográfica, três características devem ser consideradas:

- Idade: as características do ECG variam com a faixa etária e acontecem no recém-nascido (RN), lactente, crianças e adolescentes até cerca dos 16 anos de idade. Nos dois primeiros grupos essas alterações são mais rápidas (Seção 13). Também os idosos podem apresentar ondas T negativas em V1, de forma isolada e às vezes em V2. ^2^
- Biotipo: Os indivíduos longilíneos tendem a ter o coração verticalizado e os eixos resultantes principalmente da onda P e do complexo QRS comumente orientados para a direita com rotação horária nas derivações do plano frontal. Já nos brevilíneos, com corações horizontalizados, esses desvios costumam ser para a esquerda (plano frontal).
- Sexo: nos adultos do sexo feminino é comum observar ondas T negativas em precordiais direitas, inclusive com QTc maiores que os do sexo masculino e as crianças.

### 1.2. O Laudo Eletrocardiográfico 1 , 3 - 5

#### 1.2.1. Laudo Descritivo

- Análise do ritmo e quantificação da frequência cardíaca;
- Análise da duração, amplitude e morfologia da onda P e duração do intervalo PR;
- Determinação do eixo elétrico de P, QRS e T;
- Análise da duração, amplitude e morfologia do QRS;
- Análise da repolarização ventricular e descrição das alterações do ST-T, QT e U, quando presentes.

#### 1.2.2. Laudo Conclusivo

Deve conter a síntese dos diagnósticos listados nesta diretriz. Abreviaturas em laudos, textos científicos, protocolos, etc., poderão ser utilizadas, entre parênteses, após a denominação, por extenso, do diagnóstico.

#### 1.2.3. Laudo Automatizado

Com o desenvolvimento tecnológico, nos últimos anos, houve uma melhora importante na acurácia das medidas automáticas dos aparelhos disponíveis, tornando a interpretação automatizada uma ferramenta auxiliar importante no laudo médico. Ainda assim, é fundamental a conferência destas métricas automáticas por uma revisão médica, já que o laudo é um ato médico. A simples utilização das aferições automáticas (métricas e vetoriais), assim como os laudos provenientes desses sistemas, sem revisão, não são recomendadas.

#### 1.2.4. Laudo Via Internet

Os sistemas de Tele-ECG, ^6 - 8^ enviam os ECGs realizados à distância para os Centros de Referência para laudo. A técnica de execução dos ECGs (Unidades executoras), bem como a interpretação e os laudos (Centros de Referência), deverão seguir as mais recentes diretrizes nacionais e internacionais. Eles são parte integrante da Telecardiologia, que também abarca outros exames da especialidade, que são executados, registrados e transmitidos de um ponto a outro para interpretação à distância, como por exemplo, monitoração de marca-passo, Holter, gravador de eventos, entre outros. Dentre os vários benefícios da telecardiologia temos:

- Pré-atendimento ao paciente em seu local de origem;
- Redução do tempo e custo dispendido pelo paciente;
- Maior rapidez na triagem por especialistas;
- Acesso a especialistas em acidentes e emergências;
- Facilita gerenciamento dos recursos de saúde;
- Na reabilitação, aumenta a segurança do paciente pós-cirúrgico;
- Cooperação e integração de pesquisadores para compartilhamento de registros clínicos;
- Acesso a programas educacionais de formação e qualificação.

Segundo vários autores, a telecardiologia foi identificada como uma atividade social e economicamente vantajosa para os prestadores de serviço, pagadores e pacientes. Reconhecidamente é uma ferramenta útil para os locais afastados dos grandes centros.

## 2. Avaliação da Qualidade Técnica do Traçado

### 2.1. Critérios de Avaliação Técnica dos Traçados

#### 2.1.1. Calibração do Eletrocardiógrafo

Nos aparelhos analógicos a verificação da calibração se faz sempre necessária. O padrão normal deve ter 1 mV (10 mm). Nos aparelhos mais modernos (computadorizados com traçados digitalizados), a verificação do padrão do calibrador é realizada automaticamente. Os filtros devem seguir as normas internacionalmente aceitas, principalmente da AHA. Para os filtros de alta frequência de, no mínimo, 150 Hz para os grupos de adultos e adolescentes. Para crianças, até 250 Hz. Filtros com essas frequências mais baixas podem interferir na captação das espículas de marcapassos. Filtro de baixa frequência utiliza-se 0,05Hz. Alguns aparelhos usam filtros de fase bidirecional. ^9^

#### 2.1.2. Troca de Eletrodos

**Figura 2.1:**
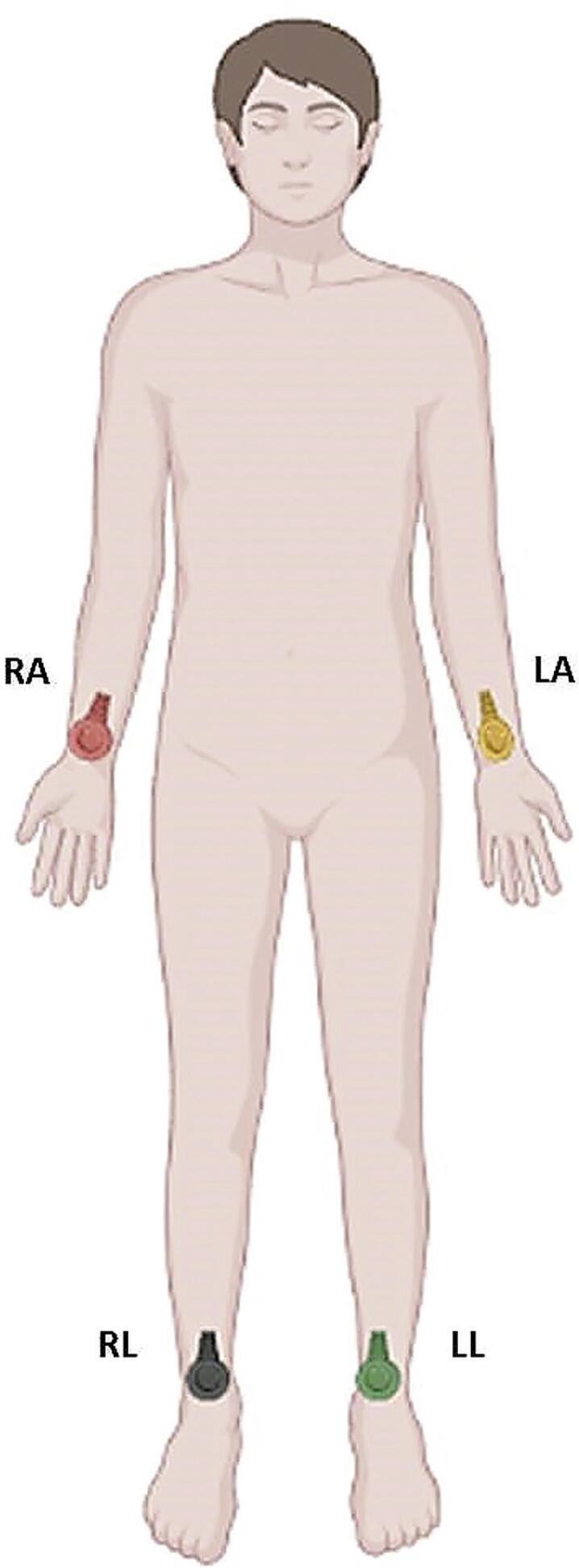
Localização dos eletrodos periféricos. RA: braço direito; LA: braço esquerdo;

RL: perna direita; LL: perna esquerda.

A Figura 2.1 mostra a posição correta dos eletrodos periféricos (braço direito (RA), braço esquerdo (LA), perna direita (RL) e perna esquerda (LL)) com suas respectivas cores vermelho, amarelo, preto e verde).

#### 2.1.2.1. Posicionamento Trocado dos Eletrodos

2.1.2.1.1. Eletrodos dos MMSS Trocados entre Si

Apresentam derivações D1 com ondas negativas e aVR com ondas positivas.

##### 2.1.2.2. Eletrodo dos MMII trocado por um eletrodo de um dos MMSS

Linha isoelétrica ou amplitude de ondas muito pequenas em D2 (braço direito) ou D3 (braço esquerdo). A troca dos eletrodos dos membros superiores com os dos inferiores mostra esse padrão em D1, pois produz uma diferença de potencial desprezível nos membros superiores.

##### 2.1.2.3. Troca de Eletrodos entre Braço Esquerdo e Perna Esquerda

É a troca de mais difícil identificação. O SÂQRS tende a desviar-se para a esquerda. Pode parecer um ECG normal, mas produz as seguintes alterações:

- onda P invertida em D3;
- D1 e D2 trocam de posição. D1 tem voltagem de QRS mais ampla e menor em D2;
- em D3 invertem-se P, QRS e T. Também são trocadas as posições de aVL com aVF. A derivação aVR não se altera.

##### 2.1.2.4. Troca de Eletrodos Precordiais

Alteração da progressão normal da onda R de V1 a V6.

##### 2.1.2.5. Eletrodos V1 e V2 Mal Posicionados

Eletrodos V1 e V2 posicionados incorretamente acima do segundo espaço intercostal podem produzir padrão rSr’ simulando atraso final de condução, ou morfologia rS de V1 a V3 e onda P negativa em V1, simulando SAE.

## 2.1.3. Outras Interferências

### 2.1.3.1. Tremores Musculares

Tremores musculares podem interferir na linha de base, mimetizando alterações eletrocardiográficas como *flutter* atrial e fibrilação ventricular ^10^ no paciente parkinsoniano.

### 2.1.3.2. Neuroestimulação

Portadores de afecções do SNC que necessitam do uso de dispositivos de estimulação elétrica artificial podem apresentar artefatos que mimetizam a espícula de marcapasso cardíaco.

### 2.1.3.3. Frio, Febre, Soluços, Agitação Psicomotora

São outras condições que produzem artefatos na linha de base e podem mimetizar arritmias como fibrilação atrial e flutter atrial.

### 2.1.3.4. “Grande Eletrodo” Precordial

A utilização de gel condutor em faixa contínua no precórdio, resultando num traçado igual de V1-V6, correspondente à média dos potenciais elétricos nestas derivações. ^3^

### 2.1.3.5. Oscilação da Linha de Base

Pode ser provocada por qualquer eletrodo mal fixado, movimentação dos membros, pela respiração ou em exames realizados com o paciente em cadeira de rodas. Nesse último caso outros artefatos também podem ser registrados.

### 2.1.3.6. Outras Interferências Elétricas e Eletromagnéticas

Esta resulta de interferências de linhas elétricas, equipamentos elétricos e telefonia celular. Para a realização do ECG deve-se solicitar ao paciente que retire todos os objetos metálicos e o telefone celular guardado na vestimenta. Os marca-passos transcutâneos podem produzir espícula, que pode ser confundida como falsa captura. O filtro utilizado também é de grande importância porque, às vezes, cria uma falsa falha de comando criando uma pausa representada por uma linha isoelétrica entre dois batimentos. ^11 - 12^

### 2.1.3.7. Alterações Decorrentes de Funcionamento Inadequado de Softwares e Sistemas de Aquisição de Sinais Eletrocardiográficos Computadorizados

A aquisição de dados por sistemas computadorizados, em alguns aparelhos eletrocardiográficos mais antigos, pode apresentar, raramente, problemas específicos e ainda não totalmente conhecidos. Como exemplo, na ausência de sinal eletrocardiográfico em um dos eletrodos, o sistema pode contrabalançar os outros sinais adquiridos e criar complexos QRS bizarros. Aparelhos eletrocardiográficos de 12 derivações simultâneas que possuem aferições automáticas de durações das ondas P e QRS podem apresentar medidas superestimadas das mesmas. Isso ocorre pois o software utiliza a onda mais precoce e a mais tardia dentre as 12 derivações para gerar tal medida.

## 3. A Análise do Ritmo Cardíaco

### 3.1. Análise da Onda P, Frequência Cardíaca e Ritmo

Estudos populacionais sobre valores de normalidade dos parâmetros eletrocardiográficos são utilizados há muitos anos como referência para nossa população, mesmo sabendo que diferenças étnicas têm influência sobre o que é considerado normal. Em 2017, dentre as diversas informações obtidas pelo estudo ELSA-Brasil, foi publicado estudo sobre valores da normalidade para a população brasileira sem doença cardíaca. ^13^

Os parâmetros que serão abordados no item 3 referem-se ao ECG de adulto. O ECG pediátrico será abordado no item 13.

#### 3.1.1. Definição do Ritmo Sinusal (RS)

Ritmo fisiológico do coração, que se origina no átrio direito alto, observado no ECG de superfície pela presença de ondas P positivas nas derivações D1, D2 e aVF, independentemente da presença ou não do complexo QRS. O eixo de P pode variar entre 0° e +90°. A onda P normal possui amplitude máxima de 2,5 mm e duração igual ou inferior a 110 ms. Podem ocorrer modificações de sua morfologia dependentes da frequência cardíaca, bem como da sua orientação (SÂP) nas derivações observadas. ^14^

#### 3.1.2. Frequência da Onda P Sinusal

A faixa de normalidade da frequência cardíaca em vigília é entre 50 bpm e 99 bpm. ^14 - 16^

## 3.2. Análise das Alterações de Ritmo Supraventricular

### 3.2.1. Definição de Arritmia Cardíaca

Alteração da formação e/ou condução do impulso elétrico através do miocárdio. ^17^ Após a definição (ou não) da presença do ritmo sinusal, busca-se a presença de arritmia cardíaca.

### 3.2.2. Arritmia Supraventricular

Ritmo que se origina acima do feixe de His. A identificação do local de origem da arritmia será usada sempre que possível. Quando não, será empregado o termo genérico supraventricular.

### 3.2.3. Presença de Onda P Sinusal

#### 3.2.3.1. Arritmia Sinusal (AS)

Geralmente fisiológica, depende do sistema nervoso autônomo, e caracteriza-se pela variação dos intervalos PP entre 160 ms e 220 ms, durante o ritmo sinusal. A variação fásica é a relacionada com a respiração (comum na criança) e a não fásica não possui essa relação.

#### 3.2.3.2. Bradicardia Sinusal (BS)

Refere-se ao ritmo sinusal com frequência inferior a 50 bpm.

#### 3.2.3.3. Bloqueio Sinoatrial de Segundo Grau

O bloqueio de saída de segundo grau da despolarização sinusal faz com que ocorra a ausência de inscrição da onda P em um ciclo. O bloqueio sinoatrial do tipo I (BSAI) se caracteriza por ciclos PP progressivamente mais curtos até que ocorra o bloqueio. O bloqueio sinoatrial tipo II (BSA II) não apresenta diferença entre os ciclos PP e a pausa corresponde a 2 ciclos PP prévios. Os bloqueios sinoatriais de primeiro grau não são visíveis ao ECG convencional. Os bloqueios de terceiro grau serão observados na forma de ritmo de escape atrial ou juncional.

#### 3.2.3.4. Bloqueios Interatriais (BIA)

Retardo da condução entre o átrio direito e o esquerdo, que pode ser classificado em primeiro grau (duração da onda P maior ou igual a 120 ms), segundo grau (padrão transitório) e terceiro grau ou avançado (onda P com duração maior ou igual a 120 ms, bifásica ou “plus-minus” em parede inferior, relacionado a arritmias supraventriculares e síndrome de Bayés). ^18 , 19^

#### 3.2.3.5. Taquicardia Sinusal (TS)

Refere-se ao ritmo sinusal com frequência superior (ou igual) a 100 bpm.

## 3.2.4. Ausência de Onda P Antes do QRS

### 3.2.4.1. Fibrilação Atrial (FA)

A atividade elétrica atrial desorganizada, com frequência atrial entre 450 e 700 ciclos por minuto e resposta ventricular variável. A linha de base pode se apresentar isoelétrica, com irregularidades finas, grosseiras ou por um misto destas alterações (ondas “ *f* ”). A ocorrência de intervalos RR regulares indica a existência de dissociação atrioventricular. Para a denominação da resposta ventricular, num ECG com FA, deve-se calcular a FC (bpm) a partir de um traçado de 6 s (número de QRS neste período multiplicado por 10). Assim, teremos as seguintes possibilidades de resposta ventricular:

1. Ritmo de FA com baixa resposta ventricular, quando a FC estiver menor ou igual a 50 bpm;
2. Ritmo de FA com controle adequado da FC (em repouso), quando a resposta ventricular estiver entre 60 e 80 bpm;
3. Ritmo de FA com controle leniente (ou inadequado) da FC (em repouso), quando a resposta ventricular estiver entre 90 e 110 bpm;
4. Ritmo de FA com resposta ventricular elevada, quando a FC estiver maior a 110 bpm.

### 3.2.4.2. Flutter Atrial

Atividade elétrica atrial organizada (macrorreentrante) que utiliza extensa região do átrio direito, sendo uma delas o istmo cavotricuspídeo (ICT). O ICT pode ser utilizado tanto no sentido anti-horário (90% dos casos) como no sentido horário (10%). Em ambas as situações, denomina-se flutter atrial comum (por utilizar o ICT). Quando no sentido horário, é chamado de comum reverso. No flutter atrial comum, as conhecidas ondas “F” apresentam frequência entre 240 e 340 bpm, bem como um padrão característico das mesmas: aspecto em dentes de serrote, negativas nas derivações inferiores e, geralmente, positivas em V1. Graus variados da condução AV podem ocorrer, sendo que quando superiores a 2:1 facilitam a observação das ondas “F”. Já no flutter atrial reverso, as ondas “F” possuem frequências mais elevadas entre 340 e 430 bpm. As ondas “F” são, além de positivas nas derivações inferiores, mais alargadas. Ao ECG, não é possível a diferenciação entre o flutter atrial comum reverso e uma taquicardia atrial esquerda (com origem na veia pulmonar superior direita). O chamado Flutter atrial incomum é aquele que não utiliza o ICT, portanto, está incluída nessa classificação a taquicardia atrial cicatricial, a taquicardia da veia cava inferior e a taquicardia por reentrada no anel mitral (todas são muito difíceis de serem diagnosticadas pelo ECG (recebem o nome genérico de taquicardia atrial).

### 3.2.4.3. Ritmo Juncional

Trata-se de ritmo de suplência ou de substituição originado na junção AV, com QRS iguais ou ligeiramente diferentes aos de origem sinusal. Trata-se de aberrância pela origem diferente do estímulo e não aberrância fásica, que depende do estímulo ser alterado pela fase 3 (precoce) ou 4 (tardio) do potencial de ação. Pode apresentar-se sem onda P visível ao ECG. Estas “posições” da onda P devem-se às velocidades de condução do estímulo elétrico aos átrios e aos ventrículos. Ao chegar antes aos ventrículos, e depois aos átrios, a onda P fica localizada dentro ou após o complexo QRS. Quando a frequência for inferior a 50 bpm é designado ritmo juncional de escape. Quando a frequência for superior a 50 bpm é chamado de ritmo juncional ativo e, se acima de 100 bpm, é chamado de taquicardia juncional.

### 3.2.4.4. Extrassístole Juncional

Batimento ectópico precoce originado na junção AV. São três as possíveis apresentações eletrocardiográficas:

- Onda P negativa nas derivações inferiores com intervalo PR curto;
- Ausência de atividade atrial pregressa ao QRS (onda P dentro do QRS);
- Onda P negativa nas derivações inferiores após o complexo QRS.

O complexo QRS apresenta-se de morfologia e duração similar ao do ritmo basal, embora aberrâncias de condução possam ocorrer (ver itens 3.2.8.1 e 3.2.8.2).

### 3.2.4.5. Taquicardia por Reentrada Nodal Comum (TRN) ^20^

Esta taquicardia utiliza a estrutura do nó atrioventricular; e tem como mecanismo eletrofisiológico a reentrada nodal. Um circuito utiliza a via rápida, no sentido ascendente, e o outro utiliza a via lenta, no sentido descendente. Se o QRS basal for normal estreito, durante a taquicardia poderemos notar pseudo-ondas “s” em parede inferior e morfologia rSr’ (pseudo r’) em V1, que refletem a ativação atrial no sentido nó AV / nó sinusal. Essa ativação retrógrada atrial, em sua maioria, ocorre em até 80 ms após o início do QRS (RP<80ms). Muitas vezes a onda de ativação atrial está dentro do QRS e, dessa forma, não é observada no ECG. A TRN comum é muito semelhante, ao ECG, com a TAV ortodrômica que será detalhada a seguir. Utiliza-se o intervalo RP para se fazer essa distinção entre elas. Nos casos de TRN com QRS alargado, faz-se necessário o diagnóstico diferencial com taquicardias de origem ventricular.

### 3.2.4.6. Taquicardia por Reentrada Atrioventricular Ortodrômica (TRAV)

Esta taquicardia por reentrada utiliza o sistema de condução normal no sentido anterógrado e uma via anômala no sentido retrógrado. O QRS da taquicardia geralmente é estreito e a onda P retrógrada, geralmente localizada no segmento ST, pode apresentar-se com morfologia diversa, dependendo da localização da via acessória. O intervalo RP é superior a 80 ms.

## 3.2.5. Presença da Onda P Não Sinusal Antes do QRS

### 3.2.5.1. Ritmo Atrial Ectópico (RAE)

O ritmo atrial ectópico corresponde a uma atividade atrial em localização diversa da região anatômica do nó sinusal. Desta forma, a onda P apresenta-se com morfologia (polaridade) diferente daquela que caracteriza o ritmo sinusal.

### 3.2.5.2. Ritmo Atrial Multifocal (RAM)

Ritmo originado em focos atriais múltiplos, com frequência cardíaca inferior a 60 bpm, reconhecido eletrocardiograficamente pela presença de, pelo menos, 3 morfologias de ondas P e 3 diferentes intervalos PR. Os intervalos PP e PR, frequentemente, são variáveis, habitualmente observa-se uma P para um QRS, podendo ocorrer ondas P bloqueadas.

### 3.2.5.3. Ritmo Juncional

Mencionado no item 3.2.4.3, caracteriza-se pelas ondas P negativas nas derivações DII, DIII e aVF, além do intervalo PR curto. Quando a frequência for inferior a 50 bpm é designado ritmo juncional de escape. Quando a frequência for superior a 50 bpm é chamado de ritmo juncional ativo e, se acima de 100 bpm, é chamado de taquicardia juncional.

### 3.2.5.4. Batimento de Escape Atrial

Durante uma interrupção temporária do automatismo sinusal normal, é possível observar um batimento “de suplência”, de origem atrial, consequente a esta inibição do nó sinusal. Caracteriza-se por ser um batimento tardio, de origem atrial, portanto com onda P de morfologia diferente da sinusal.

### 3.2.5.5. Extrassístole Atrial (EA)

Batimento ectópico atrial precoce. Pode reciclar o ciclo PP basal. Usa-se a sigla ESV para extrassístole supraventricular.

### 3.2.5.6. Extrassístole Atrial Bloqueada ou Não Conduzida

Batimento ectópico de origem atrial que não consegue ser conduzido ao ventrículo, não gerando, portanto, complexo QRS. A não condução pode ser devida à precocidade acentuada da EA, que encontra o sistema de condução intraventricular em período refratário, ou devido a doença do sistema de condução His-Purkinje. Estas EA bigeminadas, não conduzidas, podem gerar bradicardia.

### 3.2.5.7. Taquicardia Atrial (TA)

Ritmo atrial originado em região diversa do nó sinusal, caracterizado pela presença de onda P distinta da sinusal com frequência atrial superior a 100 bpm. É comum a ocorrência de condução AV variável.

### 3.2.5.8. Taquicardia Atrial Multifocal (TAMF)

Apresenta as mesmas características do ritmo atrial multifocal, com frequência atrial superior a 100 bpm.

### 3.2.5.9. Taquicardia por Reentrada Nodal Incomum

O local de origem e o circuito são similares à TRN comum (3.2.4.5), mas o sentido de ativação atrial e ventricular, pelas vias lenta e rápida, é inverso, motivo pelo qual a ativação atrial retrógrada se faz temporalmente mais tarde, com o característico intervalo RP maior que o PR. Desta forma, a TRN incomum não é um diagnóstico diferencial com a TRN comum, nem com a TAV ortodrômica.

### 3.2.5.10. Taquicardia de Coumel

Taquicardia supraventricular mediada por uma via anômala com condução retrógrada exclusiva e decremental. Caracteriza-se por uma taquicardia com intervalo RP longo e é diagnóstico diferencial com as descritas nos itens 3.2.5.7 e 3.2.5.9.

## 3.2.6. Pausas

Define-se pausa pela ausência de onda P e complexo QRS em um intervalo superior a 1,5 s e passa a apresentar importância clínica quando maior que 2,0 s. A ocorrência de pausas no traçado pode relacionar-se à presença de parada sinusal, extrassístole atrial não conduzida, bloqueio sinoatrial e bloqueio atrioventricular.

### 3.2.6.1. Parada Sinusal (PS)

Corresponde a uma pausa na atividade sinusal superior a 1,5 vezes o ciclo PP básico.

### 3.2.6.2. Disfunção do Nó Sinusal (DNS)

A incapacidade do nódulo sinusal em manter uma frequência cardíaca superior às necessidades fisiológicas para a situação do momento denomina-se disfunção do nó sinusal. Ao ECG, essa anormalidade (ou disfunção) do nó sinusal é entidade que engloba a pausa sinusal, o bloqueio sinoatrial, a bradicardia sinusal, ritmos de substituição, a fibrilação atrial, o flutter atrial, a síndrome bradi-taqui, etc. ^21^

## 3.2.7. Classificação de Taquicardias Supraventriculares Baseadas no Intervalo RP

Intervalo RP é uma medida comumente realizada para caracterizar uma taquicardia supraventricular. A mensuração é feita a partir do complexo QRS até a onda P seguinte (RP). A depender da posição desta onda P, podemos ter um RP curto (onda P encontra-se antes da metade de dois QRS) ou um RP longo (onda P encontra-se após a metade de dois QRS). Assim, as taquicardias paroxísticas supraventriculares podem ser divididas em:

- Taquicardia com RP’ curto (habitualmente até 120-140ms), como observado na taquicardia por reentrada nodal comum e na taquicardia por reentrada via feixe anômalo;
- Taquicardia com RP’ longo, como observado na taquicardia atrial, na taquicardia por reentrada nodal incomum e na taquicardia de Coumel (reentrada por feixe anômalo de condução retrógrada exclusiva e decremental). ^22^

## 3.2.8. Arritmias Supraventriculares com Complexo QRS Alargado

### 3.2.8.1. Aberrância de Condução

Um estímulo supraventricular que encontra dificuldade de propagação regional no sistema de condução, gerando um QRS com morfologia diferente, em comparação ao complexo QRS de base, o qual pode apresentar padrão de bloqueio de ramo, de bloqueio divisional ou associação de ambos.

### 3.2.8.2. Extrassístole Atrial com Aberrância de Condução

Batimento atrial reconhecido eletrocardiograficamente por apresentar onda P precoce seguida de QRS com morfologia de bloqueio de ramo, bloqueio divisional ou associação de ambos.

### 3.2.8.3. Taquicardia Supraventricular com Aberrância de Condução

Denominação genérica para as taquicardias supracitadas que se expressem com condução aberrante.

### 3.2.8.4. Taquicardia por Reentrada Atrioventricular Antidrômica

A taquicardia por reentrada utiliza uma via acessória no sentido anterógrado e o sistema de condução no sentido retrógrado. O QRS é aberrante e caracteriza-se pela presença de pré-excitação ventricular. O diagnóstico diferencial deve ser feito com taquicardia ventricular. A observação da despolarização atrial retrógrada 1:1 é importante para o diagnóstico da via anômala, e a dissociação AV para o de taquicardia ventricular.

## 4. Condução Atrioventricular

### 4.1. Definição da Relação Atrioventricular (AV) Normal

O período do início da onda P ao início do QRS determina o intervalo PR, tempo em que ocorre a ativação atrial e o retardo fisiológico na junção atrioventricular (AV) e/ou sistema His-Purkinje, cuja duração é de 120 a 200 ms, considerando FC de até 90 bpm. O intervalo PR varia de acordo com a FC e a idade, existindo quadros de correção.

#### 4.1.1. Atraso da Condução Atrioventricular (AV) 23 - 26

Ao estudarmos os atrasos, é importante lembrar da característica eletrofisiológica normal do nódulo AV, denominada condução decremental. Essa propriedade refere-se à redução da velocidade de condução do estímulo elétrico no nó AV e pode ser estimada por meio do intervalo PR no ECG convencional. Esse intervalo é considerado normal no adulto quando se encontra entre 120 a 200 ms e depende muito da idade e da frequência cardíaca.

Os atrasos da condução atrioventricular (AV) ocorrem quando os impulsos atriais sofrem retardo ou falham em atingir os ventrículos.

Anatomicamente, esses atrasos podem estar localizados no próprio nódulo AV (bloqueio nodal), no tronco His–Purkinje (bloqueio intra-His) ou abaixo dele (bloqueio infra-His). Geralmente os atrasos nodais apresentam-se com complexos QRS estreitos (< 120 ms) e possuem bom prognóstico, e podem ser expressos pelo aumento do intervalo PR. Por outro lado, é comum que os atrasos intra e infra-His cursem com complexos QRS alargados e pior evolução. Não é comum, nesses casos, a presença de intervalo PR normal.

Salientamos que o nó AV sofre importante influência do sistema nervoso autônomo, portanto, nas situações em que haja predominância do tônus parassimpático (durante sono, atletas), pode-se observar bloqueio AV de 1º grau e/ou bloqueio AV de 2º grau tipo I, sem haver lesão do nó AV.

##### 4.1.1.1. Bloqueio AV de Primeiro Grau

Nesta situação, o intervalo PR é superior a 200 ms em adultos, para FC entre 50 a 90 bpm.

##### 4.1.1.2. Bloqueio AV de Segundo Grau Tipo I (Mobitz I)

Nesta situação, o alentecimento da condução AV é gradativo (fenômeno de Wenckebach). Tipicamente, existe aumento progressivo do intervalo PR, sendo tais acréscimos gradativamente menores, até que a condução AV fique bloqueada e um batimento sinusal não consiga ser conduzido. Há, portanto, um gradual aumento do intervalo PR com concomitante encurtamento dos intervalos RR até uma onda P ser bloqueada. Pode ocorrer repetição desse ciclo por períodos variáveis, quando é possível notar que o intervalo PR após o batimento bloqueado será o menor dentre todos, e o que o sucede terá o maior incremento percentual em relação aos posteriores. A frequência de bloqueio pode ser variável, por exemplo, 5:4, 4:3, 3:2.

##### 4.1.1.3. Bloqueio AV de Segundo Grau Tipo II (Mobitz II)

Nesta situação, existe uma interrupção súbita da condução AV. Nota-se condução AV 1:1 com intervalo PR fixo e, repentinamente, uma onda P bloqueada, seguida por nova condução AV 1:1 com PR semelhante aos anteriores. A localização desse bloqueio localiza-se na região intra/infra His-Purkinje.

##### 4.1.1.4. Bloqueio AV 2:1

Caracteriza-se pela alternância de uma onda P conduzida e outra bloqueada de origem sinusal. A maior parte desse bloqueio localiza-se na região intra/infra His-Purkinje. Deve-se excluir o diagnóstico de extrassístoles atriais não conduzidas.

##### 4.1.1.5. Bloqueio AV Avançado ou de Alto Grau

Nesta situação, existe condução AV em menos de 50% dos batimentos sinusais, sendo em proporção 3:1, 4:1 ou maior. Geralmente, a presença de condução AV é notada pelo intervalo PR constante em cada batimento seguido de um QRS. A maior parte desse bloqueio localiza-se na região intra/infra His-Purkinje. Podem acontecer escapes juncionais.

##### 4.1.1.6. Bloqueio AV do Terceiro Grau ou BAV Total (BAVT)

Neste caso, os estímulos de origem sinusal não conseguem chegar aos ventrículos e despolarizá-los, fazendo com que um foco abaixo da região de bloqueio assuma o comando ventricular. Não existe, assim, correlação entre a atividade elétrica atrial e ventricular (dissociação atrioventricular), o que se traduz no ECG por ondas P não relacionadas ao QRS. A frequência do ritmo sinusal é maior que a do ritmo de escape. O bloqueio AV do terceiro grau pode ser intermitente ou permanente. Bloqueios com origem supra-hissiana podem apresentar-se com escapes de morfologia semelhantes ao do ECG basal, enquanto que a origem infra-hissiana evidencia complexos QRS largos como escapes.

##### 4.1.1.7. Bloqueio AV Paroxístico

É a ocorrência, de forma súbita e inesperada, de uma sucessão de ondas P bloqueadas.

## 4.1.2. Pré-Excitação Ventricular 27 - 30

**Figura 4.1:**
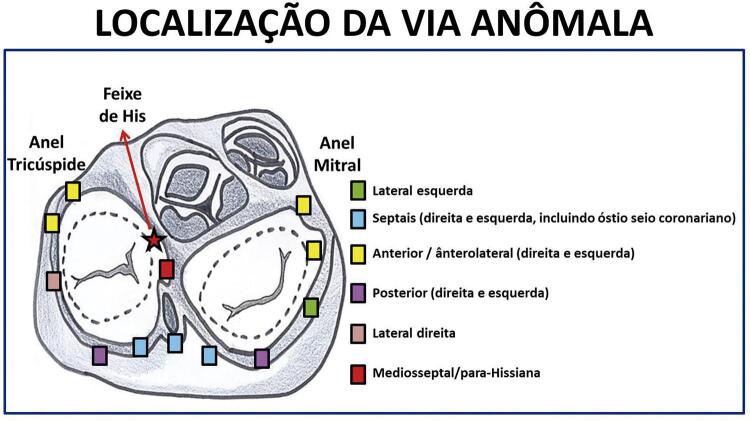
Possíveis localizações das vias anômalas nos anéis tricúspide e mitral.

Em pacientes com pré-excitação, feixes musculares persistem, de permeio ao tecido fibroso, servindo como vias acessórias da condução do estímulo elétrico entre os átrios e os ventrículos. Essas vias extras podem estar em qualquer parte do anel atrioventricular ( Figura 4.1 ). São características do padrão clássico: intervalo PR menor que 120 ms durante o ritmo sinusal em adultos e menor que 90 ms em crianças (variando com a idade e a frequência cardíaca); entalhe da porção inicial do complexo QRS (onda delta), que interrompe a onda P ou surge imediatamente após seu término; duração do QRS maior que 120 ms em adultos e maior que 90 ms em crianças; alterações secundárias de ST e T. Na presença desses achados eletrocardiográficos, a presença de taquicardia paroxística supraventricular sintomática configura a Síndrome de Wolff-Parkinson-White (WPW). A via acessória pode ser localizada anatomicamente pelo ECG. As vias laterais esquerdas são as mais comuns (50% dos casos), seguidas das posterosseptais (25%), laterais direitas (15%) e anterosseptais (10%). As regiões anteriores do anel atrioventricular são superiores, assim, vias acessórias nessa localização determinam ativação no sentido supero-inferior, com positividade da onda delta nas derivações inferiores. Já a região basal posterior é inferior, dessa maneira vias acessórias aí situadas geram ativação anômala que foge dessa região, com consequente negatividade da onda delta nas derivações inferiores. Mais raramente, deve-se fazer o diagnóstico diferencial com a situação de PR curto sem onda delta, presente na síndrome de Lown-Ganong-Levine, ^31^ e o PR normal com pré-excitação ventricular, presentes nas vias fascículo ventriculares, como na variante de Mahaim. ^32^

As vias anômalas podem ser categorizadas quando o complexo QRS é predominantemente positivo (R) em V1 e V2, o que indica uma via acessória à esquerda, e quando o QRS é negativo (QS ou rS), a via encontra-se à direita. As vias laterais esquerdas manifestam-se no ECG através de onda delta negativa nas derivações D1 e/ou aVL, positiva nas derivações D2, D3 e aVF, e em V1 e V2. As vias anômalas direitas apresentam onda delta positiva nas derivações D1, D2 e aVL e, geralmente, negativa nas derivações D3 e aVF, assim como em V1. O eixo elétrico do QRS no plano frontal é desviado para a esquerda. Já as vias posterosseptais apresentam ao ECG onda delta negativa em D2, D3 e aVF. A importância do reconhecimento das localizações das vias anterosseptais e mediosseptais está relacionada à sua proximidade ao feixe de His, trazendo maior risco durante a ablação com cateter. Em ambas as localizações, a onda delta é positiva nas derivações D1, D2 e aVL, além de negativa em D3 e aVR e positiva/isoelétrica em aVF, com eixo elétrico do QRS normal. Em 80%, a transição R/S ocorre em V2. ^32^

A análise dos complexos QRS em V1 e V2 fará a diferenciação se estão à direita ou à esquerda. ^33^

Existem vários algoritmos para a localização da via acessória, baseados na polaridade do QRS ou da via anômala. ^34 - 36^

Devemos lembrar que entidades podem simular a presença de pré-excitação (falsos WPW) como a cardiomiopatia hipertrófica e formas familiares de depósito septal de glicogênio (doença de Fabry).

## 4.1.3. Outros Mecanismos de Alteração da Relação AV Normal

### 4.1.3.1. Dissociação AV

A dissociação AV tem como causas os seguintes mecanismos: substituição, interferência, bloqueio atrioventricular e dissociação por arritmia. ^37^ Ocorrem dois ritmos dissociados, sendo um atrial, geralmente sinusal, com PP regular, e outro de origem juncional ou ventricular, também com RR regular. A frequência destes focos pode ser similar (dissociação isorritmica). O ritmo ventricular pode ser hiperautomático.

### 4.1.3.2. Ativação Atrial Retrógrada

A ativação do átrio origina-se a partir de um estímulo juncional ou ventricular, com condução retrógrada, geralmente pelo nó AV ou por uma via anômala. Observa-se QRS seguido de onda P negativa nas derivações inferiores.

## 5. Análise da Ativação Ventricular

### 5.1. Ativação Ventricular Normal

#### 5.1.1. Definição do QRS Normal

O complexo QRS é dito normal quando a duração for inferior a 120 ms em todas as derivações e amplitude entre 5 e 20 mm nas derivações do plano frontal e entre 10 e 30 mm nas derivações precordiais, com orientação normal do eixo elétrico. ^38 , 39^

#### 5.1.2. Eixo Elétrico Normal no Plano Frontal

Os limites normais do eixo elétrico do QRS no plano frontal situam-se habitualmente entre -30° e +90°.

#### 5.1.3. Ativação Ventricular Normal no Plano Horizontal

Tem como característica a transição da morfologia rS, característica de V1, para o padrão qR típico do V6, onde observa-se um progressivo aumento da amplitude da onda r e, concomitantemente, uma gradual redução da onda de V2 até V6. Os padrões intermediários de RS (zona de transição) habitualmente ocorrem em V3 e V4. ^16^

#### 5.1.4. Análise das Alterações de Ritmo Ventricular

##### 5.1.4.1. Definição de Arritmia Cardíaca

Arritmia cardíaca pode ser definida como uma alteração da frequência, formação e/ou condução do impulso elétrico através do miocárdio. ^17^

##### 5.1.4.2. Arritmia Ventricular

Arritmia ventricular é uma arritmia de origem abaixo da bifurcação do feixe de His, habitualmente expressa por QRS alargado.

##### 5.1.4.3. Análise das Arritmias Ventriculares

**5.1.4.3.1. Extrassístole Ventricular (EV) ^40^**

Apresenta-se como batimento originado precocemente no ventrículo, geralmente com pausa pós extrassistólica, quando recicla o intervalo RR. Na ausência de pausa, é chamada de extrassístole ventricular interpolada. As EV geralmente apresentam duração do QRS superior a 120ms. Excepcionalmente (EVs com origem no septo ventricular ou próximas do sistema de condução) podem apresentar-se com duração inferior a 120ms. Em relação à forma, podem ser classificadas em monormórficas (quando apresentam a mesma morfologia) ou polimórficas (apresentam mais de uma morfologia) e de acordo com sua inter-relação podem ser denominadas de isoladas, pareadas, em salvas, bigeminadas, trigeminadas, quadrigeminadas ou ocultas.

**5.1.4.3.2. Batimento(s) de Escape Ventricular(es)**

Batimento(s) de origem ventricular, tardio(s) por ser(em) de suplência. Surge(m) em consequência da inibição temporária de ritmos anatomicamente mais altos.

**5.1.4.3.3. Ritmo de Escape Ventricular – Ritmo Idioventricular**

Trata-se de ritmo com origem nos ventrículos, com FC inferior a 40 bpm, ocorrendo em substituição a ritmos anatomicamente mais altos que foram inibidos ou bloqueados.

**5.1.4.3.4. Ritmo Idioventricular Acelerado (RIVA)**

Este ritmo origina-se no ventrículo (QRS alargado), tendo FC superior a 40 bpm (entre 50 e 130 bpm, mais usualmente entre 70 e 85 bpm), em consequência de automatismo aumentado. Não é ritmo de suplência, competindo com o ritmo basal do coração. Costuma ser autolimitado e está relacionado à doença isquêmica miocárdica (reperfusão/isquemia). ^41^

**5.1.4.3.5. Taquicardia Ventricular (TV)**

A taquicardia ventricular (TV) é um ritmo ventricular que se apresenta com três ou mais batimentos sucessivos com frequência cardíaca acima de 100 bpm.

**5.1.4.3.5.1. Taquicardia Ventricular Monomórfica**

Caracteriza-se por uma TV com morfologia uniforme na mesma derivação.

**5.1.4.3.5.2. Taquicardia Ventricular Polimórfica (TVP)**

Ritmo de origem ventricular, rápido, com QRS de três ou mais morfologias. Apresenta 2 padrões característicos: *Torsade des Pointes* (TdP) e a chamada taquicardia ventricular polimórfica verdadeira. ^42^

**5.1.4.3.5.3. Taquicardia Ventricular Tipo Torsade des Pointes (TdP)**

Trata-se de taquicardia com QRS largo, polimórfica, geralmente autolimitada, com QRS “girando” em torno da linha de base (torção das pontas). Normalmente, é precedida por ciclos longo-curto (extrassístole - batimento sinusal – extrassístole) e observa-se, durante o ritmo sinusal, intervalo QT longo, o qual pode ser congênito ou secundário a fármacos, distúrbios eletrolíticos ou determinadas doenças cardíacas. ^43^

**5.1.4.3.5.4. Taquicardia Ventricular Bidirecional ^44^**

Trata-se de taquicardia de origem ventricular que, ao conduzir-se para o ventrículo, apresenta-se com o ramo direito bloqueado constantemente (raramente BRE) e as divisões anterossuperior e posteroinferior do ramo esquerdo bloqueadas alternadamente, batimento a batimento. No plano frontal do ECG alternam-se um batimento com QRS positivo, seguido de outro com QRS negativo, sucessivamente (gerando o aspecto bidirecional). Esta arritmia está relacionada a quadros de intoxicação digitálica, doença miocárdica grave por cardiomiopatia avançada e casos sem cardiopatia estrutural, como a taquicardia catecolaminérgica familiar, sendo prenúncio de taquicardia ventricular polimórfica nestes indivíduos.

**5.1.4.3.5.5. Quanto à Duração**

Classificação de acordo com sua duração em segundos: taquicardia sustentada (TVS) ou não sustentada (TVNS), se o período da arritmia for ou não superior a 30 s e/ou sem sintomas de instabilidade hemodinâmica.

**5.1.4.3.6. Batimento de Fusão**

Corresponde a batimento originado nos ventrículos que se funde com o batimento supraventricular. Ao ECG, apresenta onda P seguida de QRS alargado, que é a soma elétrica do batimento supraventricular com a extrassístole ventricular (morfologia híbrida entre o batimento supraventricular e o de origem ventricular). Os batimentos de fusão são encontrados nas seguintes situações: pré-excitação ventricular, taquicardia ventricular, parassistolia e extrassistolia ventricular.

**5.1.4.3.7. Batimento com Captura Supraventricular Durante Ritmo Idioventricular**

Trata-se de batimento originado no átrio que consegue ultrapassar o bloqueio de condução (anatômico ou funcional) existente na junção AV e despolarizar o ventrículo totalmente ou parcialmente, gerando no último caso um batimento de fusão.

**5.1.4.3.8. Parassístole Ventricular (PV)**

Corresponde ao batimento originado no ventrículo em foco que compete com o ritmo sinusal do coração (marca-passo paralelo que apresenta bloqueio de entrada permanente e de saída ocasional), sendo visível eletrocardiograficamente por apresentar frequência própria, batimentos de fusão e períodos inter-ectópicos com um múltiplo comum e períodos de acoplamento variável. ^45^

**5.1.4.3.9. Fibrilação Ventricular (FV)**

Caracteriza-se por ondas bizarras, caóticas, de amplitude e frequência variáveis. Clinicamente, corresponde a uma das formas de apresentação da parada cardiorrespiratória. Este ritmo pode ser precedido de taquicardia ventricular ou *Torsade des Pointes* , que degeneraram em fibrilação ventricular.

##### 5.1.4.4. Critérios de Diferenciação entre as Taquicardias de Complexo QRS Alargado 46-57 

**Figura 5.1:**
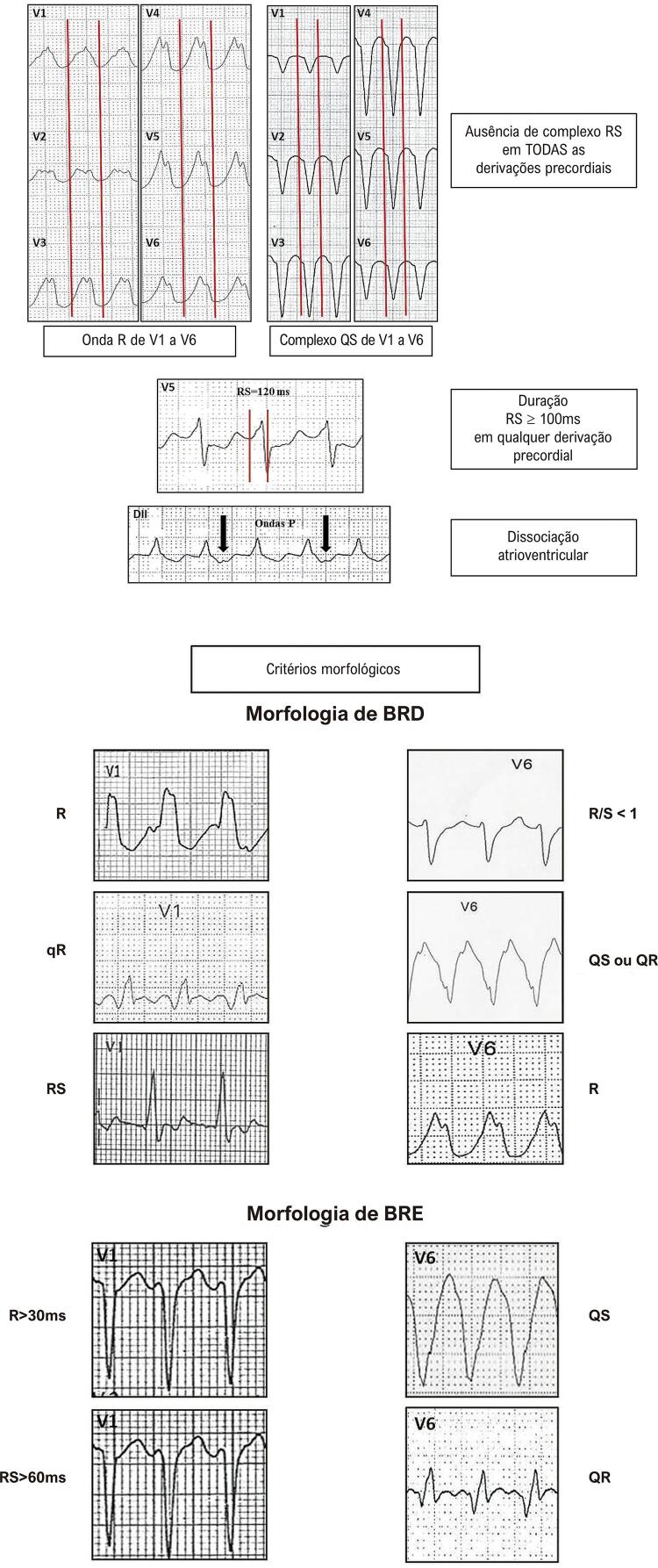
Exemplos dos quatro critérios de Brugada para o diagnóstico de taquicardia ventricular.

A maioria das taquicardias com complexo QRS largo (80%) é de origem ventricular. A presença de cardiopatia estrutural reforça esta possibilidade. Os achados de dissociação AV (frequência ventricular maior que a atrial), a presença de batimentos de fusão e/ou captura ventricular (com QRS diferente) sugerem fortemente o diagnóstico de TV. Existem algoritmos, como os de Brugada e de Vereckei ^48^ (mais utilizados), que auxiliam essa diferenciação na ausência desses sinais ( Tabela 5.1 ). ^49 - 54^ ECG’s com os achados dos critérios de Brugada e Steuer, para o diagnóstico de TV, são exemplificados nas Figuras 5.1 e 5.2, respectivamente.

**Tabela 5.1:** Critérios eletrocardiográficos para diferenciação entre taquicardia supraventricular com aberrância e taquicardia ventricular

Autor	Wellens ^49^ (1978)	Brugada ^46^ (1991)	Steuer ^51^ (1994)	Vereckei ^54^ (2008)	Pava ^55^ (2010)	Jastrzebski ^56^ ou Escore TV (2016)	Santos Neto ^57^ (2021)
Achados e Etapas da Analise para cada Algoritmo	Dissociação AV	Ausência RS precordiais	Complexos QRS predominantemente negativos de V4 a V6	R inicial aVR	Duração ^3^ 50ms início QRS até pico R em DII	Onda R dominante em V1	Polaridade predominantemente negativa nas 4 derivações: DI, DII, V1, V6
	QRS > 140 ms (BRD)	RS ^3^ 100ms	Complexo QS em uma ou mais derivações de V2 a V6	r ou q inicial > 40ms		r inicial > 40ms em V1 ou V2	Polaridade predominantemente negativa em 3 das 4 derivações
	QRS > 160 ms (BRE)	Dissociação AV	Dissociação AV	Entalhe descendente em QRS predominantemente negativo		Entalhe onda S em V1	Polaridade predominantemente negativa em 2 das 4 derivações
	Eixo QRS além de -30 ^o^	Critérios morfológicos		Relação Vi/Vt ≤ 1		R inicial aVR	
	QRS mono ou bifásico em V1 (BRD)					Duração ≥50ms início QRS até pico R em DII	
	QR ou QS em V6 (BRE)					Ausência RS precordiais	
						Dissociação AV	

**Figura 5.2:**
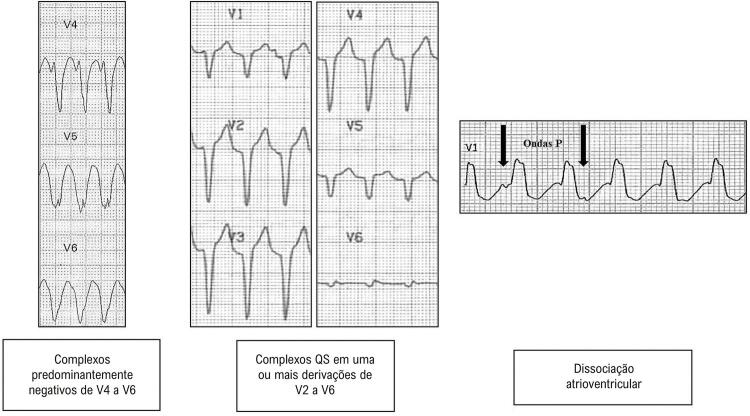
Critérios de Steuer para o diagnóstico de taquicardia ventricular.

## 6. Sobrecargas das Câmaras Cardíacas

### 6.1. Sobrecargas Atriais

#### 6.1.1. Sobrecarga Atrial Esquerda (SAE)

Aumento da duração da onda P igual ou superior a 120 ms, na derivação D2, com intervalo entre os componentes atriais direito e esquerdo maior ou igual a 40 ms. Onda P com componente negativo aumentado (final lento e profundo) na derivação V1. A área da fase negativa de pelo menos 0,04 mm/s, ou igual ou superior a 1 mm ^2^ , constitui o Índice de Morris, que apresenta melhor sensibilidade que o critério isolado de duração aumentada.

#### 6.1.2. Sobrecarga Atrial Direita (SAD)

A onda P apresenta-se apiculada com amplitude acima de 0,25 mV ou 2,5 mm. Na derivação V1 apresenta porção inicial positiva > 0,15 mV ou 1,5 mm. São sinais acessórios e indiretos de SAD: Peñaloza-Tranchesi (complexo QRS de baixa voltagem em V1 e que aumenta de amplitude significativamente em V2) e Sodi-Pallares (complexos QR, Qr, qR ou qRS em V1). Raramente isolada, frequentemente é associada à SVD.

#### 6.1.3. Sobrecarga Biatrial (SBA)

Associação dos critérios SAE e SAD.

#### 6.1.4. Sobrecarga Ventricular Esquerda (SVE) 58 - 68

Apesar do ecocardiograma apresentar elevada acurácia na identificação da SVE, o ECG, quando alterado, tem importante significado prognóstico. Dentre os critérios existentes, temos:

##### 6.1.4.1. Critérios de Romhilt-Estes ^66^

Por este critério existe SVE quando se atinge 5 pontos ou mais no escore que se segue. Dentre as limitações para a utilização deste escore temos a presença de bloqueio de ramo esquerdo e/ou fibrilação atrial, taquicardia atrial, flutter atrial e bloqueio de ramo direito.

- *Critérios de 3 pontos* – aumento de amplitude do QRS (maior ou igual a 20 mm no plano frontal e/ou maior ou igual a 30 mm no plano horizontal); padrão de *strain* na ausência de ação digitálica; e índice de Morris;
- *Critério de 2 pontos* – desvio do eixo elétrico do QRS além de -30º;
- *Critérios de 1 ponto* – aumento do tempo de ativação ventricular (TAV) ou deflexão intrinsecoide além de 40 ms; aumento da duração do QRS (>90 ms) em V5 e V6; e padrão “ *strain”* sob ação do digital.

##### 6.1.4.2. Índice de Sokolow Lyon ^60^

É considerado positivo quando a soma da amplitude da onda S na derivação V1 com a amplitude da onda R da derivação V5/V6 for >35 mm. Nos jovens, este limite pode ser de 40 mm. Não deve ser utilizado em atletas.

##### 6.1.4.3. Índice de Cornell ^58^

Quando a soma da amplitude da onda R na derivação aVL, com a amplitude da onda S de V3 for >28 mm em homens e 20 mm em mulheres.

##### 6.1.4.4. Peguero-Lo Presti 67 , 68

Este critério é considerado positivo quando a soma da amplitude da maior onda S das 12 derivações com a onda S de V4 é ≥ 28 mm em homens e ≥ 23 mm em mulheres.

##### 6.1.4.5. Alterações de Repolarização Ventricular

Onda T achatada nas derivações esquerdas (D1, aVL, V5 e V6) ou padrão tipo *strain* (infradesnivelamento do ST ≥ 0,5 mm e onda T negativa e assimétrica).

## 6.1.5. Sobrecarga Ventricular Direita (SVD) 69 - 72

### 6.1.5.1. Eixo do QRS

Eixo elétrico de QRS no plano frontal, localizado à direita de +110º no adulto.

### 6.1.5.2. Onda R Ampla

Presença de onda R de alta voltagem em V1 e V2 e ondas S profundas nas derivações opostas (V5 e V6).

### 6.1.5.3. Morfologia qR ou qRs

A morfologia qR ou qRs em V1 (ou V1 e V2) é um dos sinais mais específicos de SVD e apontam sobrecarga ventricular direita sistólica com aumento da pressão intraventricular.

### 6.1.5.4. Morfologia rsR’

Padrão trifásico (rsR’), com onda R‘ proeminente nas precordiais direitas V1 e V2 e sugere sobrecarga ventricular diastólica com aumento do volume da câmara.

### 6.1.5.5. Repolarização Ventricular

Padrão *strain* de repolarização nas precordiais direitas (V1, V2 e, às vezes, V3) (infradesnivelamento do segmento ST acompanhado da onda T negativa).

### 6.1.5.6. Critério de SEATTLE para SVD

Soma de R de V1 + S V5-V6 >10,5 mm (e desvio de eixo à direita >120°).

## 6.1.6. Sobrecarga Biventricular

- Eixo elétrico de QRS no plano frontal desviado para a direita, associado a critérios de voltagem para SVE;
- ECG típico de SVD, associado a um ou mais dos seguintes elementos:
  b.1) Ondas Q profundas em V5 e V6 e nas derivações inferiores;
  b.2) R de voltagem aumentada em V5 e V6;
  b.3) S de V1 e V2 + R de V5 e V6 com critério positivo de Sokolow;
  b.4) Deflexão intrinsecoide em V6 igual ou maior que 40 ms;
- Complexos QRS isodifásicos amplos, de tipo R/S > 50 mm, nas precordiais intermediárias de V2 a V4 (fenômeno de Katz-Wachtel).

## 6.1.7. Diagnóstico Diferencial do Aumento de Amplitude do QRS ^73^

A sobrecarga ventricular é a situação onde mais comumente ocorre o aumento da amplitude do QRS. No entanto, o QRS pode estar aumentado em indivíduos normais nas seguintes situações:

- Crianças, adolescentes e adultos jovens;
- Longilíneos;
- Atletas;
- Mulheres mastectomizadas;
- Vagotonia.

## 7. Análise dos Bloqueios (Retardo, Atraso de Condução) Intraventriculares

### 7.1. Bloqueios Intraventriculares 74 , 75

Embora a denominação “bloqueio de ramo” esteja bem estabelecida na literatura, o que ocorre são diversos graus de atrasos na propagação intraventricular dos impulsos elétricos, determinando mudanças na forma e na duração do complexo QRS. Essas mudanças na condução intraventricular podem ser fixas ou intermitentes, frequência-dependentes. Os bloqueios podem ser causados por alterações estruturais do sistema de condução His-Purkinje ou do miocárdio ventricular (necrose, fibrose, calcificação, lesões infiltrativas ou pela insuficiência vascular), ou funcionais, devido ao período refratário relativo de parte do sistema de condução gerando a aberrância da condução intraventricular.

#### 7.1.1. Bloqueio do Ramo Esquerdo (BRE) 76 , 77

a) QRS alargados com duração ≥120 ms como condição fundamental (as manifestações clássicas do BRE, contudo, expressam-se em durações iguais ou superiores a 130 ms para mulheres e iguais ou superiores a 140 ms para homens);

- Ausência de “q” em D1, aVL, V5 e V6; variantes podem ter onda “q” apenas em aVL;
- Ondas R alargadas e com entalhes e/ou empastamentos médio-terminais em D1, aVL, V5 e V6;
- Onda “r” com crescimento lento de V1 a V3, podendo ocorrer QS;
- Deflexão intrinsecoide em V5 e V6 ≥50 ms;
- Eixo elétrico de QRS entre -30° e +60°;
- Depressão de ST e T assimétrica em oposição ao retardo médio-terminal.

##### 7.1.1.1. Bloqueio de Ramo Esquerdo em Associação com Sobrecarga Ventricular Esquerda 78 - 79

O diagnóstico eletrocardiográfico de sobrecarga ventricular esquerda, em associação ao bloqueio de ramo esquerdo, não é simples devido às modificações do complexo QRS inerentes ao BRE. Os estudos mostram resultados variáveis sobre a acurácia dos critérios eletrocardiográficos para SVE.

- Sobrecarga atrial esquerda;
- Duração do QRS >150 ms;
- Onda R em aVL >11 mm;
- Ondas S em V2 >30 mm e em V3 >25 mm;
- SÂQRS além de -40° graus;
- Presença de Índice de Sokolow-Lyon ≥35 mm.

##### 7.1.1.2. Bloqueio de Ramo Esquerdo em Associação com Sobrecarga Ventricular Direita 80 (ao Menos 2 dos 3 Critérios)

- Baixa voltagem nas derivações precordiais;
- Onda R proeminente terminal em aVR;
- Relação R/S em V5 menor que 1.

## 7.1.2. Bloqueio do Ramo Direito (BRD) 81 , 82

- QRS alargados com duração ≥120 ms como condição fundamental;
- Ondas S empastadas em D1, aVL, V5 e V6;
- Ondas qR em aVR com R empastada;
- rSR’ ou rsR’ em V1 com R’ espessado;
- Eixo elétrico de QRS variável, tendendo para a direita no plano frontal;
- Onda T assimétrica em oposição ao retardo final de QRS.

### 7.1.2.1. Atraso Final de Condução

A expressão atraso final de condução poderá ser usada quando o distúrbio de condução no ramo direito for muito discreto. Pode ser uma variante dos padrões de normalidade.

## 7.1.3. Bloqueios Divisionais do Ramo Esquerdo 83 - 92

A presença de atraso que acomete, além do ramo esquerdo (tronco), as divisões deste, podem gerar desvios do SÂQRS para cima/esquerda (BDAS) ou para a baixo/direita (BDPI).

### 7.1.3.1 Bloqueio Divisional Anterossuperior Esquerdo (BDAS) 83 - 87

- Eixo elétrico de QRS ≥ -45°;
- rS em D2, D3 e aVF com S3 maior que S2; QRS com duração <120 ms;
- Onda S de D3 com amplitude maior ou igual a 15 mm;
- qR em D1 e aVL com tempo da deflexão intrinsecoide ≥ 50 ms ou qRs com “s” mínima em D1;
- qR em aVL com R empastado;
- Progressão lenta da onda r de V1 até V3;
- Presença de S de V4 a V6.

### 7.1.3.2. Bloqueio Divisional Anteromedial Esquerdo (BDAM) 88 - 90

- Morfologia qR em V1 a V4;
- Onda R ≥15 mm em V2 e V3 ou desde V1, crescendo para as derivações precordiais intermediárias e diminuindo de V5 para V6;
- Salto de crescimento súbito da onda “r” de V1 para V2 (“rS” em V1 para R em V2);
- Duração do QRS <120 ms;
- Ausência de desvio do eixo elétrico de QRS no plano frontal;
- Ondas T, em geral negativas nas derivações precordiais direitas.

Todos esses critérios são válidos na ausência de SVD, hipertrofia septal ou infarto lateral.

### 7.1.3.3. Bloqueio Divisional Posteroinferior Esquerdo (BDPI) 83 - 85 , 91 , 92

- Eixo elétrico de QRS no plano frontal orientado para a direita >+90°;
- qR em D2, D3 e aVF com R3>R2 e deflexão intrinsecoide >50 ms;
- Onda R em D3 >15 mm (ou área equivalente);
- Tempo de deflexão intrinsecoide aumentado em aVF, V5-V6 maior ou igual a 50 ms;
- rS em D1 com duração <120 ms; podendo ocorrer progressão mais lenta de “r” de V1 – V3;
- Onda S de V2 a V6.

Todos esses critérios são validos na ausência de tipo constitucional longilíneo, SVD e área eletricamente inativa lateral. ^80 , 91^

## 7.1.4. Bloqueios Divisionais do Ramo Direito ^82^

### 7.1.4.1. Bloqueio Divisional Superior Direito (BDSRD)

- rS em D2, D3 e aVF com S2>S3 (o que diferencia do BDAS do ramo esquerdo);
- Rs em D1 com onda s>2mm, rS em D1 ou D1, D2 e D3 (S1,S2,S3) com duração <120 ms;
- S empastado em V1- V2 / V5 – V6 ou, eventualmente, rSr’ em V1 e V2;
- qR em avR com R empastado.

### 7.1.4.2. Bloqueio Divisional Inferior Direito (BDIRD)

- Onda R em D2 > onda R de D3;
- rS em D1 com duração <120 ms;
- Eixo elétrico de QRS no plano frontal orientado para a direita >+90°;
- S empastado em V1 - V2 / V5 - V6 ou, eventualmente, rSr’ em V1 e V2;
- qR em aVR com R empastado.

Na dificuldade de reconhecimentos dos bloqueios divisionais direitos, pode ser utilizado o termo “atraso final da condução intraventricular”.

## 7.1.5. Associação de Bloqueios ^93^

### 7.1.5.1. BRE Associado ao BDAS

Bloqueio do ramo esquerdo com eixo elétrico de QRS no plano frontal orientado para esquerda, além de -30°, sugere a presença de BDAS.

### 7.1.5.2. BRE Associado ao BDPI

Bloqueio do ramo esquerdo com eixo elétrico de QRS desviado para a direita e para baixo, além de + 60°, sugere associação com BDPI, ou SVD, ou cardiopatia congênita.

### 7.1.5.3. BRD Associado ao BDAS

Bloqueio do ramo direito associado ao bloqueio divisional anterossuperior do ramo esquerdo - padrões comuns aos bloqueios descritos individualmente. ^94 , 95^

### 7.1.5.4. BRD Associado ao BDPI

Bloqueio do ramo direito associado ao bloqueio divisional posteroinferior do ramo esquerdo – padrões comuns aos bloqueios descritos individualmente; suspeita-se desta associação quando o SÂQRS encontra-se a + 120° ou mais para a direita.

### 7.1.5.5. BRD Associado ao BDAS e BDAM

Bloqueio de ramo direito associado ao bloqueio divisional anteromedial e anterossuperior – os padrões para estas associações seguem os mesmos critérios para os bloqueios individualmente.

### 7.1.5.6. BDAS Associado ao BDAM

Esta associação segue os mesmos critérios para os bloqueios individualmente.

### 7.1.5.7. Bloqueio de Ramo Mascarado 96 , 97

Bloqueio de ramo direito mais comumente com morfologia de R ou rR´ em V1 associado à morfologia de bloqueio de ramo esquerdo com BDAS esquerdo nas derivações do plano frontal. A onda s de D1 habitualmente está ausente ou não é maior que 1 mm.

Na presença das associações acima descritas, observa-se habitualmente acentuação nos desvios dos eixos.

## 7.1.6. Situações Especiais Envolvendo a Condução Intraventricular

### 7.1.6.1. Bloqueio Peri-infarto ^98^

Aumento da duração do complexo QRS na presença de uma onda Q anormal devido ao infarto do miocárdio nas derivações inferiores ou laterais, com aumento da porção final do complexo QRS e de oposição à onda Q (isto é, complexo QR).

### 7.1.6.2. Bloqueio Peri-isquemia 98 , 99

Quando há um aumento transitório na duração do complexo QRS acompanhado do desvio do segmento ST visto na fase aguda.

### 7.1.6.3. Fragmentação do QRS (fQRS) 99 , 100

Presença de entalhes na onda R ou S em 2 derivações contíguas na ausência de bloqueio de ramo, ou quando na presença deste, o encontro de mais de 2 entalhes. Na presença de QRS estreito é melhor visualizada nas derivações inferiores principalmente D3 e aVF. Este diagnóstico deve ser muito bem avaliado quando aparece na onda S em V1 e V2 (deve ser diferenciado dos atrasos finais de condução). Quanto maior o número de derivações com fragmentação, pior o prognóstico.

### 7.1.6.4. Bloqueio de Ramo Esquerdo Atípico ^101^

Quando da ocorrência de infarto em paciente com bloqueio de ramo esquerdo prévio, temos a presença de ondas Q profundas e largas, padrão QS em V1-V4 e QR em V5-V6, com fragmentação do QRS.

### 7.1.6.5. Bloqueio Intraventricular Parietal ou Purkinje/Músculo ou Focal ^102^

Quando o distúrbio dromotrópico localiza-se entre as fibras de Purkinje e músculo, observado em grandes hipertrofias e cardiomiopatias. Pode associar-se ao BDAS esquerdo ou SVE e a duração do QRS ≥120 ms sem apresentar morfologia de BRE ou BRE com BDAS esquerdo.

## 8. Análise do ECG nas Coronariopatias

Importante salientar que o ECG normal não exclui a presença de evento coronário, devendo-se seguir a orientação clínica específica para síndromes coronarianas agudas. ^103 , 104^

### 8.1. Critérios Diagnósticos da Presença de Isquemia Miocárdica ^105^

#### 8.1.1. Presença de Isquemia

- Fase hiperaguda – onda T apiculada e simétrica como apresentação inicial;
- *Isquemia subendocárdica* – Presença de onda T positiva, simétrica e pontiaguda;
- *Isquemia subepicárdica* – Presença de onda T negativa, simétrica e pontiaguda; atualmente atribui-se a esta alteração um padrão de reperfusão ou edema e não mais correspondendo a uma isquemia real da região subepicárdica. ^106^

#### 8.1.2. Isquemia Circunferencial ou Global 107 , 108

Situação peculiar durante episódio de angina com infradesnível do segmento ST em seis ou mais derivações, com maior intensidade em V4 a V6 acompanhado de ondas T negativas, em associação a supradesnivelamento ST > 0,5mm em aVR.

#### 8.1.3. Alterações Secundárias

São chamadas de alterações secundárias da onda T aquelas que não se enquadram na definição de ondas isquêmicas em especial pela assimetria e pela presença de outras características diagnósticas como as das sobrecargas cavitárias ou bloqueios intraventriculares.

## 8.2. Critérios Diagnósticos da Presença de Lesão

- *lesão subepicárdica* – elevação do ponto J e do segmento ST, com concavidade ou convexidade (mais específica) superior deste segmento em 2 derivações contíguas que exploram a região envolvida, de pelo menos 1 mm no plano frontal e precordiais esquerdas. Para as derivações precordias V1 a V3, considerar em mulheres ≥1,5 mm, em homens acima de 40 anos ≥2,0 mm e abaixo de 40 anos ≥2,5 mm de supradesnivelamento ST; ^109^
- *lesão subendocárdica* ^109^ – depressão do ponto J e do segmento ST, horizontal ou descendente ≥0,5 mm em 2 derivações contíguas que exploram as regiões envolvidas, aferido 60 ms após o ponto J.

Observação: o diagnóstico da corrente de lesão leva em consideração a presença concomitante de alterações da onda T e do segmento ST reconhecidas em pelo menos duas derivações concordantes.

## 8.3. Definição das Áreas Eletricamente Inativas (AEI)

Considera-se área eletricamente inativa aquela onde não existe ativação ventricular da forma esperada, sem configurar distúrbio de condução intraventricular. É caracterizada pela presença de ondas Q patológicas em duas derivações contíguas, com duração igual ou superior a 40 ms, associadas ou não à amplitude > 25% de todo QRS ou redução da onda R em área onde a mesma é esperada e deveria estar presente.

## 8.4. Análise Topográfica da Isquemia, Lesão e Necrose

### 8.4.1. Análise Topográfica das Manifestações Isquêmicas ao ECG (Meyers)

- Parede anterosseptal – Derivações V1, V2, V3;
- Parede anterior – Derivações V1, V2, V3 e V4;
- Parede anterior localizada – Derivações V3, V4 ou V3-V5;
- Parede anterolateral – Derivações V4 a V5, V6, D1 e aVL;
- Parede anterior extensa – V1 a V6 , D1 e aVL;
- Parede lateral – Derivações V5 e V6.
- Parede lateral alta – D1 e aVL;
- Parede inferior – D2, D3 e aVF.

Obs.: Os termos “parede posterior” e “dorsal” não deverão mais ser utilizados, em vista das evidências atuais de que o registro obtido por V7 a V9 refere-se à parede lateral. ^110^

### 8.4.2. Análise topográfica das manifestações isquêmicas pelo ECG em associação à ressonância magnética ^111^

- Parede septal – Q em V1 e V2;
- Parede anteroapical – Q em V1, V2 até V3-V6;
- Parede anterior média (anteromedial) – Q (qs ou r) em D1, aVL e, às vezes, V2 e V3;
- Parede lateral – Q (qr ou r) em D1, aVL, V5-V6 e/ou RS em V1;
- Parede inferior – Q em D2, D3 e aVF.

Essas localizações apresentam melhor correlação anatômica nas síndromes coronárias agudas com supradesnivelamento do segmento ST e na necrose, quando presente. As localizações topográficas, descritas acima, podem apresentar variações em virtude de cardiomegalia ou alterações estruturais importantes.

### 8.4.3. Correlação Eletrocardiográfica com a Artéria Envolvida ( Tabela 8.1 ) ^112^

**Tabela 8.1:** – Correlação entre derivações eletrocardiográficas e artéria culpada

		Supra ST			Infra ST
**Tronco coronária esquerda**		aVR			V2-V6; I,L
**Descendente anterior**	antes da 1 ^a^ septal	V1 - V4		I, L	II, III, F
**Descendente anterior**	entre septal e diagonal	V1 - V6		I, L	
**Descendente anterior longa (após crux cordis)**	após septal e diagonal	V2 - V6		I, L	V2-V6; I,L
**Coronária direita proximal**		V4 - V6	II < III, F		I, L, V1 - V3
**Coronária direita médio/distal**			II < III, F	I, L	I, L, V1 - V3
**Coronária direita distal**			II < III, F		I, L
**Coronária direita (ventrículo direito)**		V1, V3R, V4R	II < III, F		
**Circunflexa**		V4 - V6	II > III, F	I, L	V1 - V3
**Circunflexa (ventrículo direito)**		V1, V3R, V4R; V4 - V6	II > III, F	I, L	

**Figura 8.1:**
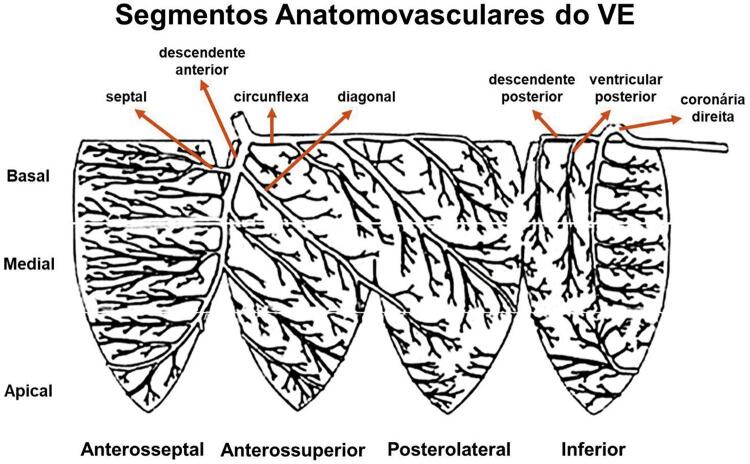
Correlação entre artéria envolvida e parede/segmento ventricular (modificado de Selvester RH et al.) ^112^

Na Figura 8.1 , encontramos a correlação entre a artéria culpada e o segmento/parede ventricular envolvido.

## 8.5. Infartos de Localização Especial

### 8.5.1. Infarto do Miocárdio de Ventrículo Direito

Elevação do segmento ST em derivações precordiais direitas (V1, V3R, V4R, V5R e V6R), particularmente com elevação do segmento ST superior a >1 mm em V4R. A elevação do segmento ST nos infartos do VD aparece por um curto espaço de tempo devido ao baixo consumo de oxigênio da musculatura do VD. Geralmente, este infarto associa-se ao infarto da parede inferior e/ou lateral do ventrículo esquerdo. ^113^

### 8.5.2. Infarto Atrial

Visível pela presença de desnivelamentos do segmento PR maiores que 0,5 mm. Pode associar-se a arritmias atriais. ^114^

## 8.6. Diagnósticos Diferenciais ^115^

### 8.6.1. Isquemia Subepicárdica

Isquemia subepicárdica deve ser diferenciada das alterações secundárias da repolarização ventricular em SVE ou bloqueios de ramos (aspecto assimétrico da onda T).

### 8.6.2. Infarto Agudo do Miocárdio (IAM) com Supra de ST

O infarto agudo do miocárdio (IAM) com supra de ST deve ser diferenciado das seguintes situações:

-  repolarização precoce;
- pericardite e miocardite;
- IAM antigo com área discinética e supradesnível persistente (aneurisma do ventrículo esquerdo);
- alguns quadros abdominais agudos como pancreatite;
- hiperpotassemia;
- síndromes catecolaminérgicas;
- síndrome de Brugada.

## 8.7. Associação de Infarto com Bloqueios de Ramo

### 8.7.1. Infarto de Miocárdio na Presença de Bloqueio de Ramo Direito (BRD *)*

O reconhecimento eletrocardiográfico de infarto do miocárdio não é dificultado na presença de BRD.

### 8.7.2. Infarto do Miocárdio na Presença de Bloqueio de Ramo Esquerdo (BRE)

A presença de BRE dificulta o reconhecimento de infarto do miocárdio associado. No BRE o atraso da condução inicia com o desaparecimento do primeiro vetor e é médio final. No infarto septal vamos observar onda R mais ampla e duradoura que a habitual (pequena ou ausente) do BRE em V1 e/ou V2, associada à onda q de V5 e V6. Nos infartos laterais teremos ondas S empastadas ou com degrau na sua fase ascendente. Nos infartos inferiores também surgirão ondas S empastadas ou entalhadas em D2, D3 e aVF. ^116^

Os desnivelamentos do segmento ST podem permitir a identificação de infarto do miocárdio recente, de acordo com os critérios definidos por Sgarbossa e cols. A partir de 5 pontos observa-se elevada acurácia no reconhecimento do infarto do miocárdio com supradesnivelamento do segmento ST. ^117^

- 5 pontos: elevação do segmento ST ≥1,0 mm em concordância com o QRS/T;
- 3 pontos: depressão do segmento ST ≥1,0 mm em V1, V2 e V3;
- 2 pontos: elevação do segmento ST ≥5,0 mm em discordância com o QRS/T.

## 9. Análise da Repolarização Ventricular

### 9.1. Repolarização Ventricular

A análise da repolarização ventricular através do ECG é extremamente complexa, pois esta representa a interação de vários sistemas capazes de se expressarem nos segmentos e ondas elétricas. O fenômeno da repolarização ganhou maior notoriedade ao trazer contribuições para a estratificação de risco de eventos arrítmicos graves e morte súbita.

#### 9.1.1. Repolarização Ventricular Normal

Período entre o final do QRS e o final da onda T ou da onda U, quando presente. Dentro deste período, os seguintes elementos devem ser analisados:

##### 9.1.1.1. Ponto J

É o ponto final da inscrição do QRS em sua interseção com o segmento ST. É útil para o diagnóstico dos desníveis do segmento ST.

##### 9.1.1.2. Segmento ST

Porção do ECG que está entre o complexo QRS e a onda T, nivelado em relação à linha de base, determinada pelo segmento PR. Geralmente, nos indivíduos normais, o ST é ligeiramente ascendente em direção à onda T (quando esta é positiva) e, ligeiramente descendente, quando a T é negativa.

##### 9.1.1.3. Onda T

A Onda T normal é assimétrica de início mais lento e final mais rápido, positiva em quase todas as derivações, habitualmente com polaridade semelhante à do QRS e de amplitude equivalente a cerca de 10% a 30% do QRS. Sempre negativa em aVR. Pode apresentar-se isoladamente negativa em V1 e/ou DIII.

##### 9.1.1.4. Onda U

Última e menor deflexão do ECG que, quando presente, inscreve-se logo após a onda T e antes da P do ciclo seguinte, de igual polaridade à T precedente e de amplitude entre 5% e 25% da mesma, na maioria das vezes. Geralmente visível apenas em frequências cardíacas baixas, tem sua gênese atribuída a:

- repolarização tardia das fibras de Purkinje;
- repolarização demorada dos músculos papilares;
- Potenciais residuais tardios do septo;
- acoplamento eletromecânico;
- atividade das células M;
- pós-potenciais de atividade gatilho (“triggered activity”).

##### 9.1.1.5. Intervalo QT (QT) e Intervalo QT Corrigido (QTc)

- *QT –* É a medida do início do QRS ao término da onda T, portanto representa a duração total da atividade elétrica ventricular;
- *QTc –* Como o QT é variável de acordo com a frequência cardíaca, habitualmente é corrigido (QTc) pela fórmula de Bazzet, onde:

$$QTc=\frac{QT^{*}}{\sqrt{RR}}$$

^*^ QT medido em milissegundos e distância RR em segundos.

A fórmula de Bazzet, ^118^ amplamente utilizada para o cálculo do QTc, apresenta, no entanto, limitações para frequências cardíacas menores que 60 bpm ou superiores a 90 bpm, devendo-se nesses casos utilizar fórmulas lineares como as de Framingham ^119^ e Hodges. ^120^

Os valores do QT e QTc não precisam ser registrados no laudo, mas sempre devem ter sua normalidade verificada. Os valores para o QTc variam com o sexo e são aceitos como normais até o máximo de 450 ms para homens e 470 ms para mulheres. Para crianças, o limite superior do normal é de 460 ms, ^121^ sendo em contrapartida considerado como QT curto os valores menores que 340 ms. ^122^

A medida do intervalo QT nos bloqueios de ramo é controversa, destacando-se recentemente a correção simplificada proposta por Bogossian: QTmBR = QTm - 0,5QRS. ^123^

## 9.1.2. Variantes da Repolarização Ventricular Normal

### 9.1.2.1. Padrão de Repolarização Precoce (RP)

Historicamente, o achado eletrocardiográfico repolarização precoce sempre foi considerado normal. Algumas publicações correlacionando a presença de um espessamento ou entalhe da porção final do QRS (também denominado de repolarização precoce) com maior mortalidade causaram um alvoroço científico sobre a benignidade desta condição. A RP caracteriza-se pela presença obrigatória de um entalhe ou espessamento no final do complexo QRS, podendo ou não haver elevação do ponto J. ^124^

A presença de onda J (espessamento ou entalhe da porção final do QRS) com aspecto retificado do ST em derivações inferiores (isoladamente ou em associação às derivações laterais) pode ser marcador de risco elétrico para o desenvolvimento de taquiarritmias ventriculares. ^125 - 129^

Nas últimas décadas, grandes avanços ocorreram relacionados à repolarização ventricular. Dentre eles temos a dispersão da repolarização ventricular como marcador da recuperação não uniforme da excitabilidade miocárdica e o reconhecimento da macro ou da microalternância cíclica da onda T. Deve-se considerar como alterações da repolarização ventricular as modificações significativas na polaridade, na duração, na morfologia dos fenômenos elétricos acima descritos.

## 10. O ECG nas Canalopatias e Demais Alterações Genéticas

### 10.1. A Genética e o ECG

O aprimoramento das técnicas de mapeamento genético possibilitou, nos últimos anos, um melhor entendimento e diferenciação de algumas entidades clínicas, potencialmente fatais, que apresentam padrão eletrocardiográfico característico. Dentro deste grupo de doenças destacam-se aquelas com coração estruturalmente normal como as canalopatias e outras afecções que cursam com acometimento miocárdico, como a cardiomiopatia hipertrófica e a displasia arritmogênica do ventrículo direito.

#### 10.1.1. Canalopatias

As canalopatias cardíacas são decorrentes de mutações genéticas ou de um mau funcionamento dos canais iônicos que culminam com alterações das fases do potencial de ação celular. Achados eletrocardiográficos específicos, com associação de síncope (em repouso ou desencadeada pelo exercício) e presença de arritmias ventriculares em coração estruturalmente normal, devem levantar a hipótese de canalopatias.

#### 10.1.1.1. Síndrome do QT Longo Congênito 129 , 130

A síndrome do QT longo congênito foi a primeira canalopatia a ser descrita e, portanto, com maior número de estudos. Esses estudos permitiram o entendimento da relação entre biologia molecular e genética e a associação com as manifestações clínicas, estratificação de risco e tratamento. Representa a principal causa de autópsia negativa em casos de morte súbita em jovens.

Sua principal característica é o prolongamento do intervalo QT corrigido ao ECG, com valores acima de 460 ms. Clinicamente, a presença de síncope ou parada cardiorrespiratória desencadeada por estresse emocional e físico deve aventar a hipótese da síndrome do QT longo. Os portadores desta síndrome apresentam risco elevado de apresentar uma taquicardia ventricular polimórfica (TVP), síncope e morte súbita (quando há degeneração desta TVP para uma fibrilação ventricular). A TVP que acontece nos indivíduas com QT longo recebe o nome de *Torsades des Pointes.* Apesar de 16 genes terem sido identificados como responsáveis pelas mutações associadas à Síndrome do QT longo (LQT), são três que respondem por 75% dos diagnósticos: KCNQ1 (LQT1), KCNH2 (LQT2) e SCN5A (LQT3). Os gatilhos para o desencadeamento das arritmias são gene-específicos, sendo o exercício mais relacionado à LQT1, emoção à LQT2 e a bradicardia à LQT3. São características ao ECG:

- LQT1: Onda T de base larga e início tardio;
- LQT2: Onda T de baixa amplitude, geralmente com entalhe;
- LQT3: Onda T tardia, após longo e retificado ST.

#### 10.1.1.2. Síndrome do QT Curto 131 - 133

Entidade descrita em 2000, caracteriza-se pelo achado de intervalo QT curto associado à fibrilação atrial e morte súbita cardíaca. O defeito genético desta condição é o aumento da função dos canais de potássio que atuam na fase 3 do potencial de ação, levando ao encurtamento do intervalo QT. Os genes relacionados a essa síndrome são o KCNH2, KCNQ1 e KCNJ2. Quando o ECG evidencia intervalos QT corrigidos curtos, com valores abaixo de 370 ms e a distância entre ponto J e o pico da onda T inferior a 120 ms, suspeita-se do diagnóstico de QT curto. A probabilidade diagnóstica aumenta com intervalos QTc menores que 340 ms.

#### 10.1.1.3. Síndrome de Brugada 134 - 137

Canalopatia causada por um defeito dos canais de sódio no epicárdio do VD, acometendo predominantemente o sexo masculino. Alguns indivíduos referem história de morte súbita familiar. Uma das características da síndrome de Brugada é a tendência a desenvolver síncopes e/ou parada cardíaca decorrente de fibrilação ventricular. Frequentemente, esses eventos ocorrem durante o repouso e sono, podendo também ser desencadeados por hipertermia e determinadas medicações, culminando com morte súbita.

Sua transmissão é autossômica dominante e é responsável por 20% das mortes súbitas com coração normal à autópsia. É geneticamente heterogênea, com envolvimento em pelo menos 13 genes. Apesar de mais de 200 mutações já terem sido descritas, a maior parte delas ocorre em genes com impacto na função dos canais de Na+ (SCN5A), responsável pelos indivíduos afetados em 20% a 25% dos casos.

A elevação de 2 mm ou mais do ponto J nas derivações V1 e V2, seguido de um descenso lento do segmento ST com convexidade superior e terminando com a inversão da onda T, caracteriza o padrão Tipo 1. O diagnóstico da síndrome de Brugada é feito pelo achado eletrocardiográfico Tipo 1 associado a sintomas.

O Tipo 2 caracteriza-se pela elevação do ponto J em V1 e V2 com menos de 2 mm, com morfologia em sela do segmento ST. Este padrão é altamente suspeito, mas; não fecha o diagnóstico. Seu caráter transitório dificulta o diagnóstico e, nos casos de dúvida, deve-se fazer o registro com os eletrodos de V1 e V2 nas derivações precordiais superiores. Os eletrodos são posicionados no 3º e 2º espaços intercostais direito e esquerdo. Este posicionamento permite melhor avaliação da via de saída do VD e aumenta a sensibilidade do ECG para o diagnóstico do padrão Tipo 1. ^138^

O achado eletrocardiográfico é denominado de fenótipo de Brugada quando não se sabe ou não há história prévia de morte súbita abortada, episódios sincopais e/ou familiares de primeiro grau com morte súbita.

Fenocópia de Brugada é uma entidade caracterizada pelo padrão eletrocardiográfico presumidamente idêntico ao da síndrome, entretanto, provocado por várias outras condições. Na fenocópia há um gradiente transmural decorrente de um acentuado entalhe no potencial de ação no epicárdio, mediado pelos canais I _to_ e perda do domo do potencial de ação,mas; não no endocárdio. Dentre as situações descritas temos: alterações metabólicas, compressão mecânica extra cardíaca, isquemia, doença miocárdica/pericárdica e determinadas medicações. ^139^

#### 10.1.1.4. Taquicardia Catecolaminérgica 140 , 141

A taquicardia catecolaminérgica acomete indivíduos durante a infância e adolescência que relatam quadros sincopais, além de história de morte súbita na família. As mutações hereditárias ou esporádicas nos canais de rianodina, responsáveis por regular o cálcio intracelular, são responsáveis por 50% a 60% dos casos de taquicardia ventricular polimórfica catecolaminérgica. O ECG de repouso pode apresentar-se dentro dos limites normais, podendo apresentar bradicardia sinusal e ondas U, sendo característica a indução de arritmia ventricular bidirecional desencadeada pelo teste ergométrico ou infusão de isoproterenol.

Achado frequente são extrassístoles ventriculares, geralmente isoladas, intermitentes, bigeminadas e pareadas que aumentam sua densidade ao exercício.

## 10.1.2. Doenças Genéticas com Acometimento Primário Cardíaco

### 10.1.2.1. Cardiomiopatia (Displasia) Arritmogênica de Ventrículo Direito 142 - 144

Doença genética que leva ao acometimento primário do VD, com substituição dos miócitos por tecido fibrogorduroso, associada a arritmias, insuficiência cardíaca e morte súbita. Ao ECG, caracteriza-se pela presença de atraso final da condução do QRS (duração >110 ms) com baixa voltagem e maior duração em V1/V2 (onda épsilon, presente em 30% dos casos), associado a ondas T negativas de V1 a V4, arredondadas e assimétricas. Associação com extrassístoles de origem no VD (que se apresentam com morfologia de BRE), podendo ter orientação superior ou inferior. O achado de ondas T negativas até V6 sugere o comprometimento do ventrículo esquerdo.

### 10.1.2.2. Cardiomiopatia Hipertrófica 145 , 146

Doença primária do coração, de base genética com herança autossômica dominante (doença genética cardíaca mais comum: 1:500 nascidos vivos), com várias mutações genéticas descritas. Ocorre hipertrofia ventricular acentuada, segmentar ou difusa. O ECG é alterado em pelo menos 75% dos pacientes, com boa sensibilidade para a faixa pediátrica. ^147^ Caracteriza-se pelo encontro de ondas Q rápidas e profundas em derivações inferiores e/ou precordiais, em geral associadas a sinais clássicos de sobrecarga ventricular esquerda e acompanhadas de alterações de ST-T características (importante infradesnivelamento do segmento ST com profundas ondas T negativas).

## 10.1.3. Doenças Genéticas com Acometimento Secundário Cardíaco

### 10.1.3.1. Distrofia Muscular ^148^

Conjunto de doenças que acometem os músculos voluntários prioritariamente e em algumas delas, ocorre acometimento dos músculos respiratórios e do coração. No ECG, os achados mais comuns são a presença de onda R ampla (relação R/S >1) em V1 e V2, onda Q profunda em V6, DI e aVL, atraso de condução pelo ramo direito, complexos QS em I, aVL, D1, D2 e D3 e alterações da repolarização ventricular.

## 11. Caracterização das Alterações Eletrocardiográficas em Situações Clínicas Específicas

### 11.1. Condições Clínicas que Alteram o ECG

Há uma miscelânea de condições em que o ECG apresenta alterações peculiares, não só nas cardiopatias como também em doenças sistêmicas, em distúrbios metabólicos e na ação de medicamentos. Em algumas delas, como nas síndromes do QT longo, de Wolff-Parkinson-White e de Brugada, o ECG é o exame mais sensível e específico para o diagnóstico. ^149^ Em outras, como no infarto do miocárdio, na pericardite e na intoxicação digitálica, o ECG é um pouco menos sensível, mas continua sendo um dos principais métodos diagnósticos. O infarto do miocárdio e a síndrome de Wolff-Parkinson-White, em razão da prevalência e da importância, são analisados em capítulos separados desta diretriz. As demais situações foram agrupadas nesta seção.

Nas condições abaixo relacionadas em ordem alfabética, analisaremos os parâmetros altamente específicos para o diagnóstico. Recomendamos, entretanto, que na conclusão dos relatórios sejam mencionadas as frases “ECG sugestivo de”, ou “ECG compatível com”.

#### 11.1.1. Ação Digitálica

Depressão de ST-T de concavidade superior (onda T “em colher”); diminuição do intervalo QTc. Na intoxicação digitálica podem ocorrer várias arritmias, predominando a extrassistolia ventricular. A presença das arritmias taquicardia bidirecional e taquicardia atrial com condução AV variável é altamente sugestiva da presença de intoxicação digitálica, bem como as bradiarritmias (bloqueios atrioventriculares de 1º grau e 2º grau tipo 1).

#### 11.1.2. Alterações de ST-T por Fármacos

Aumento do intervalo QTc. As drogas que interferem podem ser encontradas no seguinte endereço eletrônico: http://www.azcert.org/medical-pros/drug-lists/drug-lists.cfm ^150^

#### 11.1.3. Alternância Elétrica

Presença de QRS com amplitudes alternadamente maiores e menores, cíclicas e não relacionadas à respiração, em QRS sucessivos.

#### 11.1.4. Alternância da Onda T

Sua aplicabilidade clínica tem sido cada vez mais investigada. Caracteriza-se pela variação da amplitude, do formato e orientação da onda T, batimento a batimento, podendo essas variações ser episódicas ou permanentes. Ao ECG convencional, as variações podem ser macroscópicas (macroalternância) ou tão pequenas que há necessidade do auxílio de algoritmos computadorizados para sua análise (microalternância).

#### 11.1.5. Comprometimento Agudo do Sistema Nervoso Central

Ondas T negativas gigantes (mais raramente positivas), simulando isquemia subepicárdica (onda T cerebral); aumento do intervalo QTc; quando tratado, apresenta reversibilidade das alterações.

#### 11.1.6. Comunicação Interatrial (CIA)

Atraso final de condução pelo ramo direito e possível associação com sobrecarga do ventrículo direito. A associação com o desvio do eixo do QRS para cima e para a esquerda está relacionada com a CIA *ostium primum* . Não é infrequente o aparecimento de arritmias supraventriculares como fibrilação/flutter atrial.

#### 11.1.7. COVID-19

O comprometimento cardíaco decorrente da COVID-19 pode chegar a 44% dos casos graves, sendo que alterações eletrocardiográficas foram encontradas em até 93% dos hospitalizados em estado crítico. Dentre as explicações para as alterações miocárdicas com modificações do ECG temos: tempestade das citocinas, lesão pela hipóxia, alterações eletrolíticas, ruptura de placa, espasmo coronariano, microtrombo e lesão direta endotelial ou miocárdica. O ECG pode apresentar: taquicardias supraventriculares (taquicardia sinusal, fibrilação atrial, flutter atrial, taquicardia por reentrada nodal), arritmia ventricular maligna (taquicardia ventricular monomórfica e polimórfica / fibrilação ventricular), bradicardia e bloqueios AV (segundo e terceiro graus), aumento do intervalo QT, bloqueio de ramos direito e esquerdo, desvio do eixo do QRS para a direita, elevação ou depressão do segmento ST, inversão da onda T, ondas Q patológicas e sinais de tromboembolismo pulmonar (taquicardia sinusal / fibrilação atrial, sobrecarga do VD, BRD, inversão T de V1 a V3, S1Q3T3). Além disso, a COVID-19 pode desmascarar o padrão de Brugada naqueles indivíduos portadores da doença. ^151^

#### 11.1.8. Derrame Pericárdico

Efeito dielétrico (ver item 11.14), taquicardia sinusal e alternância elétrica.

#### 11.1.9. Dextrocardia

Onda P negativa em D1 e V6 e positiva em aVR; complexos QRS negativos em D1 e aVL e progressivamente menores de V1 a V6 (principal diagnóstico diferencial com a troca de eletrodos dos MMSS).

#### 11.1.10. Dextroposição

Pode ocorrer onda P negativa ou minus-plus em D1, onda Q profunda em D1 e aVL e complexos qRS a partir das precordiais direitas.

#### 11.1.11. Distúrbios Eletrolíticos

##### 11.1.11.1. Hiperpotassemia

As alterações dependem dos níveis séricos e ocorrem sequencialmente: onda T de grande amplitude, simétrica e de base estreita; redução do intervalo QTc; distúrbio de condução intraventricular (QRS alargado); diminuição da amplitude da onda P até seu desaparecimento, com presença de condução sinoventricular.

##### 11.1.11.2. Hipopotassemia

Aumento da amplitude da onda U; depressão do segmento ST e da onda T; aumento do intervalo QTU. A mensuração do intervalo QT deve ser feita, preferencialmente, na derivação aVL (onde a onda U tende a ser mais isoelétrica).

##### 11.1.11.3. Hipocalcemia

Retificação e aumento da duração do segmento ST com consequente aumento do intervalo QTc.

##### 11.1.11.4. Hipercalcemia

Encurtamento e eventual desaparecimento do segmento ST com consequente encurtamento do intervalo QT.

##### 11.1.12. Doença Pulmonar Obstrutiva Crônica (DPOC)

Efeito dielétrico (ver item 11.1.14); desvio do eixo do complexo QRS para direita; desvio posterior da zona de transição precordial do QRS para a esquerda (rS de V1 a V6). A presença de cardiopatia associada acarreta, também, na sobrecarga das câmaras direitas e desvio do eixo da onda P para a direita, próximo de +90º (P *pulmonale* ).

## 11.1.13. Drogas Antiarrítmicas

As drogas antiarrítmicas podem estar relacionadas ao fenômeno de pró-arritmia e, a seguir, apresentaremos as que mais interferem no ECG.

### 11.1.13.1. Amiodarona

Droga antiarrítmica da classe III, pode provocar o prolongamento do intervalo PR, bradicardia sinusal, aumento do intervalo QTc (sem aumento na dispersão da repolarização e, consequentemente, menor chance do aparecimento de *Torsades des Pointes* ) e alterações da onda T. Estas são melhor observadas nas derivações precordiais, tendo como característica principal uma onda T bífida ou achatada no seu pico. Tais alterações também podem ser observadas nas derivações do plano frontal, o que diferencia da onda T bífida da criança que, habitualmente, é mais observada nas derivações do precórdio.

### 11.1.13.2. Propafenona

Droga antiarrítmica da classe IC, pode provocar bloqueios AV e arritmias ventriculares complexas (extrassístoles ventriculares pareadas/em salva, TVNS e TV polimórfica), especialmente em pacientes com insuficiência coronariana.

### 11.1.13.3. Sotalol

Droga antiarrítmica da classe III, pode provocar bradicardia sinusal, bloqueios AV, aumento do intervalo QTc (TV polimórfica).

### 11.1.14. Efeito Dielétrico

Baixa voltagem do QRS em todo o traçado (<0,5 mV nas derivações do plano frontal e <1,0 mV nas derivações precordiais). Pode ser decorrente de derrame pericárdico volumoso, derrame pleural, enfisema, DPOC, obesidade mórbida e anasarca. Hipotireoidismo e doenças infiltrativas cardíacas também podem apresentar um padrão de baixa voltagem.

### 11.1.15. Embolia Pulmonar

Taquicardia sinusal, atraso final de condução no ramo direito, desvio agudo do eixo do QRS para direita, infradesnivelamento do segmento ST (V1 a V3) e negativação de ondas T de V1 a V3, preferencialmente. Pode ocorrer a clássica morfologia S1Q3T3.

## 11.1.16. Fenômeno de Ashman (ou de Gounaux-Ashman) ^17^

Condução aberrante, em batimento de origem supraventricular, que segue um ciclo longo-ciclo curto, em razão do aumento do período refratário no sistema de condução principalmente no ramo direito do His, sendo mais frequente na fibrilação atrial. ^152 , 153^ A manutenção da aberrância nos batimentos subsequentes é decorrente da condução transeptal retrógrada oculta.

## 11.1.17. Hipotermia

Bradicardia, presença de entalhe final do QRS de grande amplitude e curta duração (onda J ou de Osborn) e prolongamento do intervalo QT.

## 11.1.18. Hipotireoidismo

Pode apresentar, em casos graves (mixedema), bradicardia, baixa voltagem e alteração difusa da repolarização ventricular.

## 11.1.19. Insuficiência Renal Crônica

A presença de alterações eletrocardiográficas da hiperpotassemia e da hipocalcemia está relacionada a déficit funcional renal importante.

## 11.1.20. Pericardite ^154^

As seguintes alterações eletrocardiográficas podem ser vistas na fase aguda do processo inflamatório, geralmente ocorrendo na sequência abaixo:

- *Depressão do segmento PR* em D1, D2, aVF e de V2 a V6. Elevação do mesmo segmento em aVR, podendo; também; ocorrer em V1;
- *Segmento ST* – Elevação difusa com concavidade superior com exceção de V1 e aVR. Não ocorrem ondas q associadas;
- *Onda T* – Na fase inicial se apresenta pouco aumentada e simétrica. Caracteristicamente não apresenta inversão enquanto ocorrem manifestações de elevação do ST. Pode apresentar inversão na fase crônica da doença, após a normalização do ST. Quando esta ocorre, raramente é profunda o suficiente para assemelhar-se ao padrão da onda T isquêmica.

## 11.1.21. Quimioterápicos 155 - 158

O advento dos novos quimioterápicos tem propiciado uma sobrevida maior destes pacientes, tornando-se importante reconhecer esses efeitos. Anormalidades do ECG podem ocorrer em pacientes com injúria miocárdica induzida por drogas quimioterápicas. Com uma patogênese complexa, as alterações do ECG podem depender tanto da ação tóxica direta dos quimioterápicos sobre o substrato eletrofisiológico, como por agressão direta ao miocárdio, endocárdio e pericárdio por processos de isquemia, inflamação ou por radiação. Motivo pelo qual as alterações que acompanham a disfunção cardíaca não são específicas, mas podem, inclusive, preceder os sintomas, ou mesmo antes das alterações ao ecocardiograma. Os achados eletrocardiográficos são melhor identificados com o sequenciamento do exame. Dentre eles temos a taquicardia sinusal, o achatamento ou inversão da onda T, o aumento do QT e a baixa voltagem.

Algumas arritmias, por vezes graves como *Torsades des Pointes* , taquicardia/fibrilação ventricular, podem ocorrer no curso do tratamento.

As mais conhecidas são as antraciclinas, entretanto, há também os agentes alquilantes (ciclofosfamida), anti-metabólicos (5-Fluoroulacil), agentes anti-microtúbulos (paclitaxel), drogas imunomoduladoras (talidomida) e terapias-alvo do câncer.

## 12. O ECG em Atletas

### 12.1. A Importância do ECG do Atleta 159 - 164

As alterações eletrocardiográficas, decorrentes de adaptações fisiológicas ao treinamento (sem haver, necessariamente, a presença de alterações anatômicas/estruturais), tornam a interpretação do ECG de um atleta um desafio. Com a inclusão do ECG de repouso na avaliação pré-participação esportiva, devemos estar a par das recomendações específicas para essa população. Atualmente, os achados eletrocardiográficos, em atletas, podem ser divididos em três categorias:

#### 12.1.1. Achados Eletrocardiográficos Normais (Grupo 1)

- Aumento da voltagem do QRS para SVE ou SVD;
- Distúrbio de condução pelo ramo direito;
- Repolarização precoce / elevação do segmento ST;
- Elevação do segmento ST seguida de inversão da onda T (V1 a V4) em atletas negros;
- Inversão da onda T (V1 a V3) em atletas com idade menor 16 anos;
- Bradicardia sinusal / arritmia sinusal;
- Ritmo ectópico atrial ou juncional;
- BAV de 1º grau;
- BAV de 2º grau tipo I.

#### 12.1.2. Achados Eletrocardiográficos Anormais (Grupo 2)

- Inversão de onda T nas outras situações;
- Depressão do segmento ST;
- Ondas Q patológicas;
- Bloqueio de ramo esquerdo;
- Duração do QRS ≥ 160 ms;
- Onda Épsilon;
- Pré-excitação ventricular;
- Intervalo QT proçongado;
- Padrão de Brugada tipo 1;
- Bradicardia sinusal acentuada (< 30bpm);
- Intervalo Pr ≥ 400 ms;
- BAV de 2º grau tipo II;
- BAV de 3º grau ou total;
- Duas ou mais extrassístoles ventriculares;
- Taquiarritmias atriais;
- Arritmias ventriculares.

#### 12.1.3. Achados Eletrocardiográficos Limítrofes (Grupo 3)

- Desvio do eixo QRS para a esquerda (cima);
- Aumento do átrio esquerdo;
- Desvio do eixo do QRS para a direita;
- Aumento do átrio direito;
- Bloqueio de ramo direito.

Aqueles atletas com ECG normal não necessitam de mais investigação, desde que sejam assintomáticos e sem história familiar de doença cardíaca hereditária e/ou morte súbita. Por outro lado, os com ECG com os achados do grupo 2 devem ser investigados sobre transtornos cardiovasculares patológicos associados à morte súbita em atletas. Todas as alterações mencionadas nesse grupo podem ser manifestações estruturais no atleta. Finalmente, atletas com ECG limítrofe serão dispensados de maiores investigações desde que apresentem APENAS uma das alterações (grupo 3), além de ser assintomático e sem história familiar de doença cardíaca hereditária e/ou morte súbita. Caso apresente duas ou mais das alterações listadas (grupo 3), deve ser também investigada a presença de transtornos cardiovasculares patológicos associados à morte súbita em atletas.

## 13. O ECG em Crianças

### 13.1. Introdução

Embora os princípios gerais para a interpretação do ECG da criança e do adulto sejam bastante semelhantes, a análise do ECG pediátrico constitui um desafio à prática clínica. Tal fato se deve, em grande parte, à ocorrência de padrões eletrocardiográficos específicos na criança ( Tabela 13.1 ), relacionados à idade e às alterações anatômicas e fisiológicas próprias do desenvolvimento. ^165^

**Tabela 13.1:** Achados eletrocardiográficos normais e particulares da criança

PR mais curto que o do adulto e complexos QRS mais estreitos
Desvio do SÂQRS para a direita é normal no primeiro ano de vida
Ondas Q proeminentes nas derivações inferiores e laterais
A análise da repolarização ventricular é mais importante que a amplitude do QRS para o diagnóstico das sobrecargas ventriculares
Repolarização precoce
T negativa de V1 a V4 (até os 12 anos de idade)
Onda T bífida ou entalhada nas precordiais direitas
Onda U proeminente
Alterações comuns e fisiológicas do ritmo: Onda U proeminente Arritmia sinusal pronunciada, ritmo atrial baixo fisiológico Bloqueios atrioventriculares de primeiro grau e de segundo grau tipo I fisiológicos Extrassístoles atriais e ventriculares eventuais

O ECG do recém-nascido reflete as repercussões da circulação fetal sobre o ventrículo direito e as alterações anatomofisiológicas decorrentes da transição para a circulação neonatal. Até a 32 ^a^ semana de gestação, o ventrículo esquerdo é maior que o direito. A partir dessa fase até o final da gestação, prevalece o ventrículo direito devido ao aumento progressivo da resistência vascular pulmonar. ^166^ Ao nascimento, a aeração dos pulmões leva à queda acentuada da pressão arterial pulmonar, enquanto a remoção da placenta e fechamento do canal arterial elevam a resistência vascular sistêmica. ^167^ Em geral, ao final do primeiro mês de vida, o ventrículo esquerdo se iguala ao direito para depois predominar anatomicamente sobre o ventrículo direito. ^166 , 167^ A maioria das mudanças adaptativas acontecem ao nascimento e durante o primeiro ano de vida. O amadurecimento do sistema nervoso autônomo, o crescimento corporal e alterações na posição do coração ocorrem de maneira progressiva até a fase adulta. ^168^ Como resultado, o ECG normal muda rapidamente nas primeiras semanas de vida da criança, e somente por volta dos dois a três anos de idade a criança começa a apresentar gradativamente padrões eletrocardiográficos semelhantes aos de um adulto. ^167^

### 13.2. Aspectos Técnicos

O ECG de crianças deve incluir as doze derivações clássicas, que podem ser complementadas pelas derivações V3R e V4R quando há suspeita de sobrecarga das câmaras direitas. ^169^ Artefatos são comuns e em geral se devem a posicionamento inadequado dos eletrodos, deformidades na parede torácica, e movimentos (voluntários ou não) próprios de cada faixa etária. ^170^

Na população pediátrica, a variação nos valores normais de diversos parâmetros eletrocardiográficos com a idade faz da consulta a tabelas e gráficos, prática corriqueira e necessária. ^170^ Grande parte desses valores, particularmente os relacionados ao primeiro ano de vida, deriva dos dados canadenses de Davignon et al., ^171^ que, apesar da existência de estudos mais recentes, ^172^ permanecem como principal referência na prática clínica ( Tabela 13.2 ). Ainda é discutível se esses dados podem ser extrapolados para a população brasileira. Há, atualmente, dois estudos baseados na população brasileira. Um deles, com quase cem recém-nascidos a termo com ecocardiograma normal na primeira semana de vida, mostrou parâmetros eletrocardiográficos diferentes dos de Davignon. ^173^ O segundo, numa população acima de um ano de idade, incluiu mais de um milhão de crianças. ^174^ A leitura computadorizada e automática do ECG tem acurácia questionável em pediatria e seu uso rotineiro ainda não é recomendável. ^170^

**Tabela 13.2:** Parâmetros eletrocardiográficos de normalidade de acordo com a idade

	0-1 dia	1-3 dias	3-7 dias	7-30 dias	1-3 meses	3-6 meses	6-12 meses	1-3 anos	3-5 anos	5-8 anos	8-12 anos	12-16 anos
FC	94 - 155	91 - 158	90 - 166	106 - 182	120 - 179	105 - 185	108 - 169	89 - 152	73 - 137	65 - 133	62 - 160	60 - 120
P (mV)	0,01 - 0,28	0,03 - 0,28	0,07 - 0,29	0,07 - 0,30	0,07 - 0,26	0,04 - 0,27	0,06 - 0,25	0,07 - 0,25	0,03 - 0,25	0,04 - 0,25	0,03 - 0,25	0,03 - 0,25
PR D2 (segundos)	0,08 - 0,20	0,08 - 0,14	0,07 - 0,15	0,07 - 0,14	0,07 - 0,13	0,07 - 0,15	0,07 - 0,16	0,08 - 0,15	0,08 - 0,16	0,09 - 0,16	0,09 - 0,17	0,09 - 0,18
QRS (segundos)	0,02 - 0,10	0,02 - 0,07	0,02 - 0,07	0,02 - 0,08	0,02 - 0,08	0,02 - 0,08	0,03 - 0,08	0,03 - 0,08	0,03 - 0,07	0,03 - 0,08	0,04 - 0,09	0,04 - 0,09
SÂQRS	59 - 189	64 - 197	76 - 191	70 - 160	30 - 115	7 - 105	6 - 98	7 - 102	6 - 104	10 - 139	6 - 116	9 - 128

*FC: frequência cardíaca; mV: milivolts.*

### 13.3. Parâmetros Eletrocardiográficos e suas Variações

O ECG de criança deve ser avaliado sistematicamente e de acordo com a faixa etária ( Tabela 13.2 ). Sua análise deve considerar da mesma forma que no ECG de adulto: ritmo, frequência cardíaca, onda P (eixo, amplitude e duração), condução atrioventricular, complexo QRS (eixo, duração e morfologia), segmento ST, onda T e onda U. Devem ser realizados de rotina a medida do intervalo QT e o cálculo do QT corrigido. ^175^

#### 13.3.1. Frequência Cardíaca e Ritmo Sinusal

A massa contrátil e complacência ventricular são relativamente menores na criança, particularmente durante o primeiro ano de vida. Como resultado, seu débito cardíaco depende basicamente da frequência cardíaca (FC), que é bem mais elevada em crianças que em adultos. Um recém-nascido saudável pode apresentar FC de 150 a 230 bpm, conforme o seu grau de atividade. A FC normal aumenta do primeiro dia até o primeiro e o segundo mês de vida e retorna a valores próximos aos registrados ao nascimento no sexto mês. A partir de então, a FC cai progressivamente para, por volta dos 12 anos, chegar a valores considerados normais para adultos. ^168^

##### 13.3.1.1. Possíveis Alterações

**13.3.1.1.1. Arritmia Sinusal**

Bastante frequente em crianças, em geral é fásica e se relaciona à respiração. ^165^ É menos pronunciada em frequências cardíacas mais elevadas e em neonatos, principalmente na primeira semana de vida.

**13.3.1.1.2. Taquicardia Sinusal**

Ritmo sinusal com FC acima do 98 ^o^ percentil para a idade, em geral menor que 220 bpm. ^170 , 171^ A taquicardia sinusal pode ter diversas causas, sendo as mais frequentes: atividade física, febre (aumento da FC em 10 bpm para cada grau Celsius de elevação na temperatura corporal), anemia e desidratação. ^170^

**13.3.1.1.3. Bradicardia Sinusal**

Ritmo sinusal com frequência cardíaca abaixo do 2 ^o^ percentil para a idade ^170 , 171^ ( Tabela 13.2 ). Pode ter várias etiologias, como infecções, insuficiência respiratória, hipotermia, hipotireoidismo e aumento da pressão intracraniana. Em neonatos, a ocorrência de bradicardia sinusal transitória pode estar associada à passagem transplacentária de anticorpos anti-Ro/SSA, principalmente em mães portadoras de lúpus eritematoso sistêmico ou outras doenças do tecido conjuntivo. Por fim, pacientes com canalopatias cardíacas, como a síndrome do QT longo tipo 3 e a síndrome de Brugada, podem manifestar bradicardia sinusal.

**13.3.1.1.4. Outras Bradicardias**

O prolongamento súbito do intervalo P-P é comum, ocorre em quase metade dos neonatos normais e um sexto dos adolescentes. Essas pausas com frequência se relacionam a um aumento no tônus vagal ^165^ e algumas podem ser sucedidas por batimentos de escape supraventriculares ou ventriculares. ^168^

## 13.3.2. A onda P e a Atividade Elétrica Atrial

As características de ativação dos átrios permanecem relativamente constantes ao ECG em todas as idades. A determinação do eixo da onda P é crucial para a determinação da região de origem do ritmo, do situs víscero-atrial e da posição cardíaca. ^170^ O eixo da onda P (SÂP) sinusal está entre 0 e +90 graus. A onda P normal não deve ultrapassar 0,12 s de duração e 2,5 mm de amplitude, parâmetros que pouco variam nas diferentes faixas etárias da criança ( Tabela 13.2 ).

### 13.3.2.1. Possíveis Alterações

**13.3.2.1.1. Sobrecargas Atriais**

A sobrecarga atrial direita produz um aumento na amplitude da onda P, melhor visualizado em DII.

A sobrecarga atrial esquerda se caracteriza pelo aumento da duração total da onda P (conforme o percentil para a idade) e/ou da sua deflexão final em V1 (> 40ms em duração e > 0,1 mV em amplitude). ^168^

**13.3.2.1.2. Ritmo Juncional**

Caracterizado por mudanças na morfologia da onda P e diminuição do intervalo PR, usualmente associadas à lentificação gradual da frequência sinusal. O ritmo juncional pode ocorrer em até um terço das crianças normais e tem duração variável. É mais comum durante o sono, mas pode ocorrer na vigília e, em geral, não tem significado patológico.

## 13.3.3. Intervalo PR e a Condução Atrioventricular

O intervalo PR aumenta com a idade, é inversamente proporcional à FC e varia conforme o tônus autonômico ( Tabela 13.2 ).

### 13.3.3.1. Possíveis Alterações

**13.3.3.1.1. Bloqueios Atrioventriculares**

Episódios de bloqueio atrioventricular de primeiro grau e de segundo grau tipo I ocorrem em cerca de 10% das crianças e até 20% dos adolescentes normais, eventualmente ocorrendo períodos de bloqueio atrioventricular do tipo 2:1. São mais frequentes durante o sono, mas podem também ocorrer na vigília, principalmente em indivíduos vagotônicos e atletas. ^165^

Os bloqueios atrioventriculares de segundo grau tipo II e avançado, e o de terceiro grau (bloqueio atrioventricular total – BAVT) são geralmente patológicos, e podem ocorrer de maneira isolada ou se associar a malformações cardíacas complexas. A forma isolada do BAVT congênito incide em 1:20.000 nascidos vivos e comumente se relaciona à passagem transplacentária dos anticorpos maternos anti-Ro/SSA e anti-La/SSB. ^168^

**13.3.3.1.2. Intervalo PR curto e Pré-excitação Ventricular**

O intervalo PR curto pode ser detectado nos casos de ritmos atriais baixos ou juncionais e em doenças de acúmulo, como as de Pompe e de Fabry. ^168 , 170^

A pré-excitação ventricular caracteriza-se pelo encurtamento do intervalo PR associado à onda delta. ^170^ A pré-excitação ventricular intermitente não é incomum entre recém-nascidos e crianças. Mesmo quando persistentes, as alterações eletrocardiográficas da pré-excitação podem ser sutis em crianças e detectadas apenas através das derivações precordiais médias (V3-V4). A síndrome de Wolff-Parkinson-White (WPW) tem incidência de 0,15 a 0,3% na população pediátrica em geral. Verifica-se aumento da prevalência de pré-excitação ventricular em indivíduos portadores de cardiomiopatia hipertrófica, anomalia de Ebstein, L-transposição das grandes artérias e tumores cardíacos.

## 13.3.4. Atividade Elétrica Ventricular

As alterações mais acentuadas da atividade elétrica ventricular ocorrem durante o primeiro ano de vida da criança. Nos primeiros dias de vida, o eixo elétrico do QRS (SÂQRS) orienta-se para a direita e para baixo no plano frontal, pode variar entre 55° e 200 ^o^ e reflete o predomínio do VD sobre o VE, menos evidente nos traçados de recém-nascidos pré-termo, uma vez que no feto com menos de 32 semanas, o VE é maior que o VD. À medida que a criança cresce, o SÂQRS se devia para a esquerda e, quando a criança completa seis meses, está ao redor de 65º. ^166^ No plano horizontal, o eixo do QRS orienta-se para a direita e para frente ao nascimento. Ainda durante a primeira semana de vida, o SÂQRS desvia-se para a esquerda, mas mantém orientação anterior, resultando no aumento da onda R em V6 com persistência de R pura em V1. O desvio do eixo do QRS para trás no plano horizontal é gradativo. Desta forma, a onda R diminui lentamente em V1 no decorrer do primeiro ano de vida, mesmo quando já exibe padrões normais em V5 e V6. ^166^ A morfologia dos complexos QRS nas derivações precordiais muda durante o desenvolvimento da criança e é ditada pelas alterações do eixo de ativação elétrica ventricular. Observam-se:

- Amplitude da onda R de V1 cresce durante o primeiro mês de vida da criança e depois diminui lentamente por vários anos. Sua amplitude nessa derivação deve ser < 18 mm no primeiro ano de vida e < 10 mm após;
- Do nascimento até os seis meses, a R de V1 é maior que a R de V6. A amplitude da R em V1 torna-se praticamente igual à de V6 entre os seis e doze meses. A partir de então, a amplitude da R aumenta em V6 e diminui em V1 progressivamente;
- Ondas Q são normais e podem ser bastante pronunciadas nas derivações inferiores e precordiais laterais esquerdas, representando a ativação septal, embora estejam ausentes em DI e aVL. A amplitude das ondas Q varia conforme a idade da criança e a derivação analisada. Sua duração não deve ultrapassar o valor de 0,03 s ( Tabela 13.2 ).

Em neonatos, o QRS pode ser bastante estreito – em geral menor que 0,08 s. Sua duração aumenta progressivamente com a idade, principalmente a partir do terceiro ano de vida ( Tabela 13.2 ).

### 13.3.4.1. Possíveis Alterações

**13.3.4.1.1. Alterações do Eixo e da Amplitude do QRS**

O desvio do eixo para a esquerda pode ser observado em diversas doenças, dentre elas defeitos do septo ventricular, atresia tricúspide e síndrome de WPW, mas pode ser uma variante do normal. O desvio do eixo para a direita pode acontecer na Síndrome de Noonan mesmo na ausência de hipertensão pulmonar importante e na sobrecarga de VD. ^170^

- Sobrecarga do ventrículo direito: pode ser suspeitada na presença de onda T positiva em V1 após a primeira semana de vida e do aumento das amplitudes da R, em V1, e da S, em V6. O padrão QR em V1 é comumente visto nos casos de sobrecargas pressóricas e o rSR’ nos quadros de sobrecarga de volume do VD; ^168^
- Sobrecarga do ventrículo esquerdo: o ECG tem acurácia limitada para detecção da sobrecarga do VE em crianças. Os sinais que mais auxiliam no diagnóstico da SVE são aumento da S em V1, aumento da amplitude da R em V6 e anormalidades da onda T em V5 e V6; ^168^
- Sobrecarga bicameral (VD + VE): resulta em complexos amplos e isodifásicos nas derivações precordiais médias – sinal de Katz-Wachtel. A soma de R+S > 60 mm em V4 é bastante específica e pode ocorrer, por exemplo, nos casos de defeitos amplos do septo interventricular. ^170^

**13.3.4.1.2. Alterações das Ondas Q**

Ondas Q patológicas podem ser vistas em crianças com coronária anômala, pré-excitação ventricular, miocardites, miocardiopatias e distrofias musculares. ^175^ São frequentes no ECG de pacientes com cardiomiopatia hipertrófica, principalmente nas derivações anterolaterais (V4 a V6, DI e aVL) e, geralmente, estão associadas a sinais de sobrecarga ventricular, alterações do segmento ST e da onda T. ^176^ Deve-se ressaltar que a presença de onda Q em V1 é sempre patológica.

**13.3.4.1.3. Distúrbios da Condução Intraventricular**

O diagnóstico do bloqueio de ramo em crianças é determinado pela duração do QRS e a idade do paciente ( Tabela 13.2 ). O bloqueio de ramo direito pode ocorrer em algumas formas de cardiopatia, como a anomalia de Ebstein, e após cirurgia corretiva de malformações congênitas, como a tetralogia de Fallot e a comunicação intraventricular. Formas congênitas isoladas de bloqueio de ramo, tanto direito ou esquerdo, são raras. Atresia tricúspide, comunicação interatrial do tipo *ostium primum* , coronária anômala e defeitos do septo atrioventricular podem se associar ao bloqueio divisional anterossuperior do ramo esquerdo. O achado de bloqueio de ramo esquerdo é menos frequente em crianças. A presença de BRE em pacientes com cardiomiopatias graves resulta de acometimento significativo do VE / sistema de condução e geralmente carrega um mau prognóstico. ^168 , 170^

**13.3.4.1.4. Onda Épsilon e a Cardiomiopatia Arritmogênica do Ventrículo Direito**

Ver item 10.1.2.1.

## 13.3.5. Repolarização Ventricular

A repolarização ventricular é avaliada no ECG de superfície através da medida do intervalo QT e da análise da morfologia do segmento ST, da onda T e da onda U, nas diferentes derivações. ^168^

### 13.3.5.1. Intervalo QT

A duração do intervalo QT guarda relação inversa com a frequência cardíaca – quanto maior a FC, menor o intervalo QT e vice-versa. Em crianças, certas peculiaridades devem ser analizadas: ^168^

- O intervalo QT deve ser medido em DII, V5 e V6 – utilizar o maior deles para o cálculo do QTc;
- Em FC mais altas, a onda P pode se sobrepor à onda T, dificultando a mensuração do QT, principalmente se prolongado;
- A onda U pode ser bastante proeminente em crianças e não deve ser computada no intervalo QT se estiver bem separada da T. Quando ocorrer fusão entre T e U, ou se a U for bastante ampla (>50% da T), a técnica da tangente deve ser utilizada;
- Nos casos de arritmia sinusal importante, o QTc deve ser calculado através da média das medidas obtidas em vários ciclos cardíacos;
- Aos 4 dias de vida, crianças de ambos os gêneros têm QTc médio de 400 ± 20 ms. Por volta dos dois meses, ocorre um prolongamento fisiológico do QTc (média 410 ms), que diminui progressivamente até os seis meses, quando retorna aos valores registrados na primeira semana de vida;
- O intervalo QTc normal em crianças é de até 440 ms (percentil 97,5); ^168^
- Apesar de seu uso rotineiro como triagem cardiovascular em pediatria ainda estar em debate, o ECG tem papel crucial no diagnóstico precoce de cardiopatias arritmogênicas letais que se manifestam na infância e na adolescência, com destaque para a síndrome do QT longo (vide a seguir).

**13.3.5.1.1. Possíveis Alterações**

**13.3.5.1.1.1. Síndrome do QT Longo**

Manifesta-se principalmente durante a infância e a adolescência – poucos pacientes têm sintomas durante o primeiro ano de vida. ^177^ Morte súbita é a apresentação inicial da SQTL em até 12% dos casos. ^177^ Apesar de a doença ser relativamente rara, esforços empregados para a sua triagem se justificam pela eficácia do tratamento precoce na prevenção da morte súbita. ^168^ O diagnóstico diferencial deve ser feito com causas secundárias de prolongamento do QTc – vide item 11 para maior detalhamento. Durante os primeiros meses de vida, filhos de mães portadoras de doenças autoimunes que expressam o Anti-Ro/SSA podem apresentar QTc bastante prolongado, achado em geral transitório e que se normaliza por volta do sexto mês. ^168^

**13.3.5.1.1.2. Síndrome do QT Curto**

Ver item 10.1.1.2.

### 13.3.5.2. Segmento ST

O desnivelamento do segmento ST deve ser sempre medido com relação à linha isoelétrica que geralmente está na altura do segmento PQ. Em neonatos e bebês, a altura do segmento TP (linha isoelétrica entre onda T e onda P seguinte) é mais indicada como referência para a linha de base. ^164^

**13.3.5.2.1. Possíveis Alterações**

**13.3.5.2.1.1. Desnivelamentos do Segmento ST**

Discretos desnivelamentos do ST são comuns durante o primeiro mês de vida, quando em geral são < 2 mm. Supradesnivelamentos de até 3 mm ocorrem com alguma constância nas precordiais direitas e constituem achado normal, principalmente a partir de um ano de idade. ^168^ Sobrecargas ventriculares, cardiomiopatias, pericardites, pré-excitação ventricular, anomalia coronariana, fármacos, dentre outros, podem alterar a repolarização ventricular, levando ao supra ou infradesnivelamento do segmento ST. Apesar de pouco sensível, o infradesnivelamento do ST tem boa especificidade para o diagnóstico de sobrecarga ventricular. Casos de origem anômala do tronco coronariano esquerdo (saindo da artéria pulmonar) manifestam-se como infarto anterior extenso usualmente depois do primeiro mês de vida. ^170^

**13.3.5.2.1.2. Repolarização Precoce**

Vide item 9.1.2.1.

**13.3.5.2.1.3. Padrão eletrocardiográfico de Brugada**

O padrão de Brugada é raro em crianças e sua frequência é bem menor na população pediátrica que na adulta. ^177^ Maior detalhamento no item 10.1.1.3.

### 13.3.5.3. Onda T

Ao nascimento, ondas T positivas nas derivações precordiais direitas são normais e se devem provavelmente à adaptação fisiológica do VD às novas características hemodinâmicas e menor elasticidade miocárdica. Em crianças normais, após o segundo ou terceiro dia de vida, a onda T passa a se orientar para trás e para a esquerda, tornando-se negativa em V1 ao final da primeira semana. Dos sete dias aos sete anos de idade, ondas T positivas em V1 em geral se associam à SVD. ^170^ A onda T pode permanecer negativa de V1 a V4 – padrão juvenil – até os 12-14 anos, quando se torna positiva de V2 a V6. A persistência de T negativas nessas derivações após essa idade pode ser considerada variante do normal em 1-3% dos casos e, portanto, deve ser investigada. ^170 , 178 , 179^ Pericardites, miocardites, cardiomiopatias, isquemia miocárdica, sobrecargas ventriculares e distúrbios hidroeletrolíticos podem também levar a alterações da T. Ondas T simétricas, negativas e amplas nas derivações precordiais não são incomuns em pacientes com cardiomiopatia hipertrófica. A presença de lesões cerebrais agudas graves em crianças pode cursar com ondas T negativas e de longa duração, em várias derivações, alteração conhecida como “T cerebral” (ver item 11.1.5).

### 13.3.5.4. Onda U

Nem sempre é visível ao ECG, mas pode ser proeminente em crianças, em casos de hipocalemia, uso de antiarrítmicos e síndrome do QT Longo.

## 13.4. Distúrbios do Ritmo Cardíaco

Os critérios eletrocardiográficos utilizados para a avaliação de arritmias cardíacas em crianças seguem os utilizados para adultos. Vide item 3.

## 13.5. Reconhecimento do Situs, da Posição Cardíaca e da Inversão Ventricular

O reconhecimento do *situs* através do ECG baseia-se fundamentalmente na orientação da onda P, que se inscreve positivamente em D1 e V6 no *situs solitus* e negativamente, no *inversus* . ^166^ Nesse caso, a inversão de eletrodos e o ritmo atrial esquerdo são os principais diagnósticos diferenciais.

No plano frontal, em pacientes com *situs solitus* e levocardia, o SÂP e o SÂQRS situam-se no quadrante inferior esquerdo. No *situs inversus* com dextrocardia, o eixo da P e do QRS estão localizados no quadrante inferior direito. O SÂP e o SÂQRS encontram-se em quadrantes diferentes quando há discordância entre *situs* e posição cardíaca, como na dextrocardia com *situs solitus* , que comumente se associa a cardiopatias congênitas complexas. ^170^

A orientação dos primeiros vetores (5-20 ms) do QRS é importante na determinação da posição dos ventrículos. Na inversão ventricular, os primeiros vetores orientam-se para a esquerda e não se observam ondas Q em D1 e V6. ^166^

## 14. O ECG durante Estimulação Cardíaca Artificial

### 14.1. Estimulação Cardíaca Artificial (ECA)

Basicamente, o ECG do portador de dispositivos cardíacos eletrônicos implantáveis (DCEI) se caracteriza pela presença ou ausência de *espículas* (artefato que resulta da emissão de energia para estimulação artificial dos átrios e/ou dos ventrículos).

Com exceção dos monitores implantáveis ( *Loop Recorder* ), todos os demais DCEI (marca-passos, ressincronizadores cardíacos e cardioversores-desfibriladores implantáveis – Tabela 14.1 ) são capazes de emitir um impulso elétrico (representado por espícula), especialmente para tratamento de bradicardias; por isso, geralmente, a interpretação do ECG do portador de DCEI não permite identificar o tipo de dispositivo em uso. O funcionamento adequado, bem como o reconhecimento de disfunções do sistema, por outro lado, podem ser reconhecidos ao ECG. A identificação da espícula é fundamental para o reconhecimento do ECG de um portador de dispositivo implantável. A programação de estimulação em modo unipolar ou bipolar determinará o tamanho da espícula. Na primeira opção, a diferença de potencial se dá entre a carcaça do gerador do dispositivo e a ponta do eletrodo, o que determinará uma diferença de potencial com um vetor de grande amplitude. Consequentemente, observar-se-ão espículas com grande amplitude. Na segunda opção, bipolar, essa diferença de potencial ocorre entre os polos na ponta do eletrodo, portanto, o vetor gerado pela diferença de potencial será pequeno e as espículas registradas nesse modo apresentar-se-ão pequenas (às vezes quase imperceptíveis).

**Tabela 14.1:** Tipos de DCEI e indicações clássicas.

DCEI	Propriedades básicas	Indicação principal
**MP convencional**	Estimulação atrial e/ou ventricular	Bradiarritmias
**Ressincronizador**	Estimulação atriobiventricular	Insuficiência cardíaca refratária com bloqueio de ramo esquerdo
**CDI**	Estimulação atrial e/ou ventricular e terapias anti-taquiarritmias ventriculares	Prevenção de morte súbita cardíaca
**CDI-RC**	Estimulação atriobiventricular Terapias anti-taquiarritmias ventriculares	Insuficiência cardíaca refratária com bloqueio de ramo esquerdo Prevenção de morte súbita cardíaca

DCEI: dispositivos cardíacos eletrônicos implantáveis; CDI: cardioversor-desfibrilador implantável; RC ressincronizador cardíaco.

**Figura 14.1:**
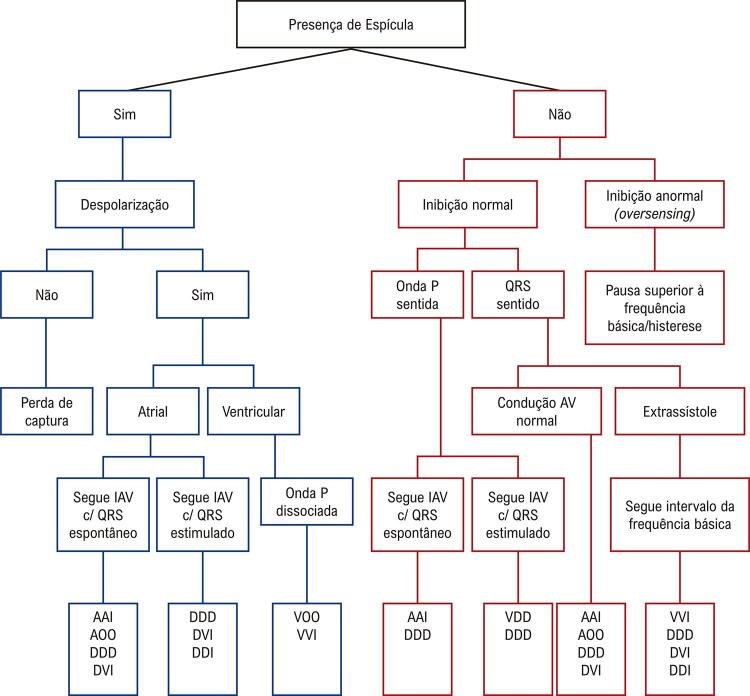
Algoritmo de interpretação do eletrocardiograma do portador de DCEI. 181

Os termos e a codificação (código de 5 letras – Tabela 14.2 ) utilizados para descrever as propriedades dos DCEI seguem uma padronização internacional (em inglês) idealizada pela *North American Society of Pacing and Electrophysiology* (NASPE) e pelo *British Pacing and Electrophysiology Group* (BPEG). ^180^ Na Figura 14.1 pode-se observar o algoritmo de identificação do modo de operação dos DCEI.

**Tabela 14.2:** Código de 5 letras para identificação eletrocardiográfica do modo de operação dos DCEI

I Câmara Estimulada	II Câmara Sentida	III Resposta à Sensibilidade	IV Modulação em Frequência	IV Funções Multisítio
O: Nenhuma	O: Nenhuma	O: Nenhuma	O: Nenhuma	O: Nenhuma
V: Ventrículo	V: Ventrículo	T: Trigger		A: Atrial
A: Átrio	A: Átrio	I: Inibida		V: Ventricular
D: Dual (A+V)	D: Dual (A+V)	D: Dual (A+V)		D: Dual (A+V)
S: Câmara única (A ou V)	S: Câmara única (A ou V)		R: Modulação em Frequência	

Trigger: deflagrar.

#### 14.1.1. Termos Básicos

- Espícula – Corresponde ao estímulo elétrico emitido pelo DCEI;
- Captura – Despolarização tecidual artificial provocada pela emissão da espícula;
- Frequência básica – Frequência de estimulação (atrial e/ou ventricular) sem interferência de batimentos espontâneos;
- Intervalo atrioventricular (IAV) – Intervalo entre uma atividade atrial espontânea (sentida) ou estimulada (espícula) e o estímulo ventricular;
- Intervalo interventricular (IVV) – Intervalo entre duas espículas ventriculares, programável por telemetria, disponível em ressincronizadores cardíacos e que eventualmente pode ser identificada ao ECG de repouso;
- Limite máximo de frequência (LMF) *–* Frequência máxima de estimulação. Nos geradores de câmara única a frequência máxima é atingida com a ativação do sensor de variação de frequência. Nos geradores de câmara dupla a frequência máxima é alcançada em resposta à sensibilidade atrial (frequência das ondas P) ou também por ativação do sensor.
- Sensibilidade *–* Capacidade de reconhecimento de eventos elétricos espontâneos atriais (P) ou ventriculares (QRS);
- Inibição normal *–* A atividade estimulatória é inibida pelo ritmo intrínseco (ausência de espículas).

#### 14.1.2. Análise das Características Eletrocardiográficas dos DCEI

- DCEI normofuncionante *–* Quando se observa captura e sensibilidade normais;
- Perda de captura atrial e/ou ventricular (intermitente ou persistente) *–* Ausência de despolarização da câmara estimulada ( *espícula* presente, mas sem deflagrar onda P ou QRS *);*
- Falha de sensibilidade:
  c.1) Sensibilidade excessiva (“ *oversensing* ”) – Exagerada sensibilidade que resulta na identificação equivocada de um sinal elétrico que não corresponde à despolarização da câmara relacionada (interferência eletromagnética, miopotenciais, onda T, etc);
  c.2) Sensibilidade diminuída (“ *undersensing* ”) – Incapacidade de reconhecimento da despolarização espontânea. Pode ocorrer por programação inadequada ou por modificações da captação do sinal intrínseco (o sistema não “enxerga” a onda P ou o QRS).
- Batimentos de fusão *–* Correspondem à ativação artificial do tecido cardíaco de forma simultânea à despolarização espontânea, provocando complexos híbridos. A *espícula* do MP é seguida de onda P (fusão atrial) ou QRS (fusão ventricular), cujas características morfológicas são intermediárias entre batimento capturado e espontâneo;
- Batimentos de pseudofusão – Ativação espontânea do tecido cardíaco, simultânea à emissão da *espícula* do MP, que não tem efeito sobre a onda P ou QRS (pseudofusão atrial e ventricular, respectivamente); a morfologia da onda que segue a *espícula* é igual à onda espontânea;
- Taquicardia mediada pelo marca-passo – Arritmia restrita aos DCEI atrioventriculares, caracterizada pela deflagração ventricular a partir de onda P retrógrada. Trata-se, portanto, de uma arritmia por movimento circular em que o sistema de estimulação cardíaca artificial faz o papel de componente anterógrado do circuito, cuja porção retrógrada é anatômica (via normal ou anômala);
- Taquicardia conduzida pelo marca-passo *–* Taquiarritmia que envolve DCEI atrioventriculares, caracterizada pela presença de arritmia supraventricular que, sentida pelo canal atrial, deflagra capturas ventriculares em frequências elevadas, mantendo certas características da arritmia espontânea;
- Taquicardia induzida pelo marca-passo – Alterações da sensibilidade ou interferências eletromagnéticas que provocam arritmias atriais ou ventriculares.

## 15. Tele-eletrocardiografia

A telemedicina é definida como a prestação de serviços de saúde através do uso de informação e tecnologias de comunicação, em situações nas quais um profissional de saúde e um paciente (ou dois profissionais de saúde) não se encontram no mesmo local. ^182^ Os sistemas de tele-eletrocardiografia (Tele-ECG) registram o traçado eletrocardiográfico feito a distância, por diferentes meios e tecnologias de transferência de dados, com a análise e interpretação do traçado eletrocardiográfico por um médico distante do paciente, e retorno do laudo por meios eletrônicos. A tele-ECG está ligada ao próprio desenvolvimento da eletrocardiografia – já em 1905, Einthoven descreveu a transmissão transtelefônica do ECG do hospital acadêmico até o laboratório de fisiologia na Universidade de Leiden, a 1,5 km de distância. ^183^

Com o desenvolvimento do ECG (ECG) computadorizado ^184^ associado a sistemas capazes de transmitir os traçados eletrocardiográficos pela internet, tornou-se possível a disponibilização do ECG, bem como seu laudo realizado por um especialista em tempo real, para localidades distantes dos grandes centros. Serviços de tele-ECG começaram a ser implementados no Brasil na primeira década do século XXI, com efeitos sobre a melhoria do acesso da população ao diagnóstico eletrocardiográfico e reconhecimento precoce de alterações eletrocardiográficas relevantes e potencialmente fatais. ^185^

Para a implementação e o funcionamento de um serviço de tele-ECG, uma infraestrutura específica é necessária ( Tabela 15.1 ). A central de leitura dos ECG deve contar com uma equipe de cardiologistas, de especialistas em tecnologia da informação (TI) e de suporte administrativo. Uma estrutura completa de TI com computadores, hardwares, softwares, sistema de proteção e armazenamento de dados é imprescindível para o funcionamento do serviço. As unidades remotas de saúde que realizarão o ECG devem ser preparadas com eletrocardiógrafo digital aprovado pelos órgãos federais responsáveis, conexão com a internet, aparelhos e serviços para comunicação por áudio ou vídeo com a central, além de treinamento operacional para todos os profissionais envolvidos. ^182 , 186^ Recomenda-se a transmissão do sinal eletrocardiográfico original ou de imagens geradas pelo próprio eletrocardiógrafo ou por escâneres profissionais, evitando-se a digitalização com distorções ou baixa qualidade, que podem dificultar ou impedir a análise do traçado. ^182^

**Tabela 15.1:** Características técnicas para implementação da tele-eletrocardiologia

NORMAS TÉCNICAS
Registro ANVISA
ABNT NBR IEC 60601-1 (norma geral de segurança)
ABNT NBR IEC 60601-1-1 (segurança de sistemas eletromédicos)
ABNT NBR IEC 60601-1-2 (compatibilidade eletromagnética)
ABNT NBR IEC 60601-1-4 (sistemas eletromédicos programáveis)
ABNT NBR IEC 60601-2-25 (segurança de eletrocardiógrafos)
ABNT NBR IEC 60601-2-251 (norma de segurança, incluindo desempenho essencial de eletrocardiógrafos, gravador e analisador monocanal e multicanal)
**PRÉ-REQUISITOS MÍNIMOS GERAIS DA MÁQUINA**
Desktop ou notebook
1 entrada USB 2.0 ou 3.0 (ao menos)
Leitor de CD/DVD
Memória de 4GB
Processador Intel Pentium
Windows 7, 8 ou 10
HD de 250GB ou superior
**RECOMENDAÇÕES**
Possuir 12 derivações
Realizar traçado com qualidade alta (1.200 amostra/segundo/canal)

A tele-eletrocardiografia tem se mostrado uma estratégia eficaz para racionalização do acesso à propedêutica complementar, diagnóstico precoce, priorização de encaminhamentos e organização de listas de espera nos sistemas de saúde, com melhora na relação custo benefício, bem como na assistência à saúde ( Tabela 15.2 ). ^187^

**Tabela 15.2:** Benefícios da tele-eletrocardiografia ^187^

Diagnóstico eletrocardiográfico rápido permitindo identificações de casos normais e diferentes do normal
Atendimento (pré) ao paciente em seu local de origem
Acesso a especialistas em acidentes e emergências
Redução do tempo e custo dispendido pelo paciente
Agilização da triagem por especialistas
Auxílio e orientação a não especialistas
Facilita gerenciamento dos recursos de saúde
Na reabilitação, aumenta a segurança do paciente pós-cirúrgico
Cooperação e integração de pesquisadores para compartilhamento de registros clínicos
Acesso a programas educacionais de formação e qualificação
Segunda opinião

A realização do tele-ECG pré-hospitalar em pacientes com síndrome coronariana aguda, especialmente em áreas rurais, apresentou impacto na redução do tempo porta-balão, bem como na mortalidade a longo prazo. ^188 , 189^ Houve melhoria na detecção de fibrilação atrial ^190^ e de algumas canalopatias, como síndrome de Brugada. ^191^ Ademais, o uso dos bancos de dados dos serviços de tele-eletrocardiografia também são de grande importância para o desenvolvimento de estudos epidemiológicos nacionais. ^192^

O constante desenvolvimento da tecnologia voltada à saúde abriu novas perspectivas no cenário da tele-eletrocardiografia. A aplicação de técnicas de inteligência artificial (IA) na eletrocardiografia se encontra em exponencial crescimento, com bons resultados no diagnóstico automático de anormalidades eletrocardiográficas. ^193 , 194^ A utilização da inteligência artificial também pode levar ao desenvolvimento de novos marcadores de risco cardiovascular. ^195^ O surgimento de aparelhos “vestíveis” como a cinta com monitor cardíaco, o adesivo com registro eletrocardiográfico, os *smartphones* e *smartwatches* possibilitou a identificação mais precoce de possíveis arritmias cardíacas, principalmente a fibrilação atrial. ^196^ Esses aparelhos portáveis e de fácil utilização tornaram possível a rápida gravação do ritmo cardíaco durante o dia a dia do paciente, em qualquer ambiente ou horário, seguida de uma interpretação automática imediata por IA. A maior limitação de sua utilização ainda é o custo. Não podemos ignorar, como consequências da modernidade, um possível aumento da carga de trabalho (registros e envios das informações feitos pelos pacientes aos seus médicos), os casos de falsos positivos decorrentes de artefatos, além um aumento da carga emocional, em alguns pacientes, ao “descobrir” uma arritmia cardíaca. Esperamos que os próximos anos possam clarear o papel das novas metodologias e tecnologias na prática clínica, mas a expectativa é que, com tais avanços, a eletrocardiografia ganhe novos usos e aplicações.
