# Supplementary material for: Diretriz da Sociedade Brasileira de Cardiologia sobre a Análise e Emissão de Laudos Eletrocardiográficos – 2022
Source: Arq Bras Cardiol. 2022 Sep 8;119(4):638–80. [Article in Portuguese] doi: 10.36660/abc.20220623 (PMC9563889; doi:10.36660/abc.20220623)
Supplement: Translation

# Translation: Brazilian Society of Cardiology Guidelines on the Analysis and Issuance of Electrocardiographic Reports – 2022

**Authors:** Nelson Samesima, Epotamenides Good God, Jose Claudio Lupi Kruse, Marcelo Garcia Leal, Claudio Pinho, Francisco Faustino de A. C. França, João Pimenta, Acácio Fernandes Cardoso, Adail Paixão, Alfredo Fonseca, Andrés R. Pérez-Riera, Antonio Luiz Pinho Ribeiro, Bruna Affonso Madaloso, Bráulio Luna, Carlos Alberto Rodrigues de Oliveira, César José Grupi, Dalmo Antonio Ribeiro Moreira, Elisabeth Kaiser, Gabriela Miana de Mattos Paixão, Gilson Feitosa, Horacio Gomes Pereira, José Grindler, José Luiz Aziz, Marcos Sleiman Molina, Mirella Facin, Nancy M. M. de Oliveira Tobias, Patricia Alves de Oliveira, Paulo César R. Sanches, Ricardo Alkmin Teixeira, Severiano Melo Atanes, Carlos Alberto Pastore

**Affiliations:** 1 Brasil Instituto do Coração (InCor) do Hospital das Clínicas da Faculdade de Medicina da Universidade de São Paulo (HCFMUSP), São Paulo, SP – Brasil; 2 Brasil Hospital SOCOR, Belo Horizonte, MG – Brasil; 3 Brasil Instituto de Cardiologia do Rio Grande do Sul,Porto Alegre, RS – Brasil; 4 Brasil Hospital das Clínicas de Ribeirão Preto, Ribeirão Preto, SP – Brasil; 5 Brasil Pontifícia Universidade Católida (PUC), Campinas, SP – Brasil; 6 Brasil Clínica Pinho, Valinhos, SP – Brasil; 7 Brasil Instituto Dante Pazzanese de Cardiologia, São Paulo, SP – Brasil; 8 Brasil Hospital do Servidor Público Estadual, São Paulo, SP – Brasil; 9 Brasil Serviço de Eletrocardiologia do Hospital das Clínicas da Faculdade de Medicina da Universidade de São Paulo (HCFMUSP), São Paulo, SP – Brasil; 10 Brasil Hospital Unimec, Vitória Da Conquista, BA – Brasil; 11 Brasil Hospital das Clínicas da Faculdade de Medicina da Universidade de São Paulo (HCFMUSP), São Paulo, SP – Brasil; 12 Brasil Faculdade de Medicina do ABC, Santo André, SP – Brasil; 13 Brasil Universidade Federal de Minas Gerais (UFMG), Belo Horizonte, MG – Brasil; 14 Brasil Hospital São Paulo, Universidade Federal de São Paulo (UNIFESP), São Paulo, SP – Brasil; 15 Brasil Grupo Fleury, São Paulo, SP – Brasil; 16 Brasil Hospital das Clínicas da Universidade Federal de Minas Gerais (UFMG), Belo Horizonte, MG – Brasil; 17 Brasil Hospital Santa Izabel, Santa Casa da Bahia, Salvador, BA – Brasil; 18 Brasil Clínica São Paulo, São Paulo, SP – Brasil; 19 Brasil Clínica Médica de Cardiologia Dr. Paulo Sanches, Campinas, SP – Brasil; 20 Brasil Hospital Renascentista, Pouso Alegre, MG – Brasil; 21 Brasil Faculdade de Medicina da Universidade do Vale do Sapucaí (UNIVÁS), Pouso Alegre, MG – Brasil; 22 Brasil Policlínica Maceió SS, São Paulo, SP – Brasil

**Table:** 

Brazilian Society of Cardiology Guidelines on the Analysis and Issuance of Electrocardiographic Reports – 2022	
The report below lists declarations of interest as reported to the SBC by the experts during the period of the development of these statement, 2021	
Expert	Type of relationship with industry
Acácio Fernandes Cardoso	Nothing to be declared
Adail Paixao Almeida	Nothing to be declared
Alfredo José da Fonseca	Nothing to be declared
Andrés R. Pérez-Riera	Nothing to be declared
Antonio Luiz Pinho Ribeiro	Other relationships Performance, in the previous year, as a medical auditor for health insurance companies or the like: - Consulting in Artificial Intelligence in Health for Unimed-BH Participation in government-related regulatory authorities or advocacy authorities in cardiology: - Working with the Ministry of Health in digital health agreements and support for Primary Health Care
Braulio Luna Filho	Nothing to be declared
Bruna Affonso Madaloso	Financial declaration A - Economically relevant payments of any kind made to (i) you, (ii) your spouse/partner or any other person living with you, (iii) any legal person in which any of these is either a direct or indirect controlling owner, business partner, shareholder or participant; any payments received for lectures, lessons, training instruction, compensation, fees paid for participation in advisory boards, investigative boards or other committees, etc. from the brazilian or international pharmaceutical, orthosis, prosthesis, equipment and implants industry: - SBC - Basic Electrocardiography Course Clinical Research; Immune BioSolutions Inc.
Carlos Alberto Pastore	Nothing to be declared
Carlos Alberto Rodrigues de Oliveira	Nothing to be declared
Cesar José Grupi	Nothing to be declared
Claudio Pinho	Other relationships Funding of continuing medical education activities, including travel, accommodation and registration in conferences and courses, from the brazilian or international pharmaceutical, orthosis, prosthesis, equipment and implants industry: - Bayer: Xarelto
Dalmo Antônio Ribeiro Moreira	Other relationships Funding of continuing medical education activities, including travel, accommodation and registration in conferences and courses, from the brazilian or international pharmaceutical, orthosis, prosthesis, equipment and implants industry: - Bayer, Abbott, Libbs, Astra Zeneca, Daichy Sankio
Elisabeth Kaiser	Nothing to be declared
Epotamenides Maria Good God	Nothing to be declared
Francisco Faustino de Albuquerque Carneiro de França	Nothing to be declared
Gabriela Miana de Mattos Paixão	Nothing to be declared
Gilson Soares Feitosa Filho	Nothing to be declared
Horacio Gomes Pereira Filho	Nothing to be declared
João A Pimenta de Almeida	Nothing to be declared
Jose Claudio Lupi Kruse	Nothing to be declared
José Grindler	Nothing to be declared
Jose Luis Aziz	Financial declaration A - Economically relevant payments of any kind made to (i) you, (ii) your spouse/partner or any other person living with you, (iii) any legal person in which any of these is either a direct or indirect controlling owner, business partner, shareholder or participant; any payments received for lectures, lessons, training instruction, compensation, fees paid for participation in advisory boards, investigative boards or other committees, etc. from the brazilian or international pharmaceutical, orthosis, prosthesis, equipment and implants industry: - Astrazeneca: hypertension and diabetes; Daiichi Sankyo: hypertension and atrial fibrillation
Marcelo Garcia Leal	Nothing to be declared
Marcos Sleiman Molina	Other relationships Any economically relevant equity interest in companies in the healthcare or education industry or in any companies competing with or supplying to SBC: - Owner of a private clinic in the city of Mogi das Cruzes
Mirella Facin	Nothing to be declared
Nancy Maria Martins De Oliveira	Nothing to be declared
Nelson Samesima	Nothing to be declared
Patricia Alves de Oliveira	Nothing to be declared
Paulo César Ribeiro Sanches	Nothing to be declared
Ricardo Alkmim Teixeira	Financial declaration A - Economically relevant payments of any kind made to (i) you, (ii) your spouse/partner or any other person living with you, (iii) any legal person in which any of these is either a direct or indirect controlling owner, business partner, shareholder or participant; any payments received for lectures, lessons, training instruction, compensation, fees paid for participation in advisory boards, investigative boards or other committees, etc. from the brazilian or international pharmaceutical, orthosis, prosthesis, equipment and implants industry: - Daichii-Sankyo: Lixiana; Boehringer-Ingelheim: Pradaxa, Jardiance; Biotronik /Abbott / Medtronic: cardiac implantable electronic devices Other relationships Funding of continuing medical education activities, including travel, accommodation and registration in conferences and courses, from the brazilian or international pharmaceutical, orthosis, prosthesis, equipment and implants industry: - Biomedical: laser extraction baths
Severiano Atanes Netto	Nothing to be declared

## Content

Introduction

1. Standards for the Analysis and Issuance of

Electrocardiographic Reports

1.1. Standards for Electrocardiographic Analysis

1.2. The Electrocardiographic Report

**1.2.1. Descriptive Report**

**1.2.2. Final Report**

**1.2.3. Automated Report**

1 **.2.4. Reports Via the Internet**

2. Tracing Technical Quality Analysis

2.1. Tracing Technical Evaluation Criteria

**2.1.1. Calibration of the Electrocardiograph**

**2.1.2. Lead Reversal**

**2.1.2.1. Lead Switches**

**2.1.2.1.1. Transposition of Upper Limb Electrodes**

**2.1.2.2. Lower Limb Electrode Swapped for Upper Limb Electrodes**

**2.1.2.3. Left Arm Electrode Swapped for Left Leg Electrode**

**2.1.2.4. Precordial Electrode Reversals**

**2.1.2.5. Misplacement of the V1 and V2 Electrodes**

**2.1.3. Other Interferences**

**2.1.3.1. Muscle Tremors**

**2.1.3.2. Neurostimulation**

**2.1.3.3. Cold, Fever, Hiccups, and Psychomotor Agitation**

**2.1.3.4. “Large Precordial Electrode”**

**2.1.3.5. Baseline Wander**

**2.1.3.6. Other Electrical and Electromagnetic Interferences**

**2.1.3.7. Alterations Due to the Malfunction of Software and**

**Computerized Electrocardiographic Signal Acquisition Systems**

3. Heart Rhythm Analysis

3.1. Analysis of the P Wave, HR, and Rhythm

**3.1.1. Definition of Sinus Rhythm**

**3.1.2. Frequency of the Sinus P Wave**

3.2. Analysis of Supraventricular Rhythm Alterations

**3.2.1. Definition of Cardiac Arrhythmia**

**3.2.2. Supraventricular Arrhythmia**

**3.2.3. Presence of Sinus P Wave**

**3.2.3.1. Sinus Arrhythmia**

**3.2.3.2. Sinus Bradycardia**

**3.2.3.3. Second-degree Sinoatrial Block**

**3.2.3.4. Interatrial Blocks (IAB)**

**3.2.3.5. Sinus Tachycardia**

**3.2.4. Absence of P Wave before the QRS**

**3.2.4.1. Atrial Fibrillation (AFib)**

**3.2.4.2. Atrial Flutter**

**3.2.4.3. Junctional Rhythm**

**3.2.4.4. Junctional Extrasystole**

**3.2.4.5. Common atrioventricular nodal reentrant tachycardia (AVNRT)**

**3.2.4.6. Orthodromic AV Reciprocating Tachycardia (AVRT)**

**3.2.5. Occurrence of a Non-sinus P Wave Before the QRS Complex**

**3.2.5.1. Ectopic Atrial Rhythm**

**3.2.5.2. Multifocal Atrial Rhythm**

**3.2.5.3. Junctional Rhythm**

**3.2.5.4. Delayed Atrial Beat**

**3.2.5.5. Premature Atrial Complex (PAC)**

**3.2.5.6. Blocked or Non-conducted PAC**

**3.2.5.7. Atrial Tachycardia**

**3.2.5.8. Multifocal Atrial Tachycardia**

**3.2.5.9. Uncommon AV Nodal Reentrant Tachycardia (AVNRT)**

**3.2.5.10. Permanent Junctional Reciprocating Tachycardia**

**(Coumel Tachycardia)**

**3.2.6. Pauses**

**3.2.6.1. Sinus Arrest**

**3.2.6.2. Sinus Node Dysfunction**

**3.2.7. Classification of Supraventricular Tachycardias Based on**

**the RP Interval**

**3.2.8. Supraventricular Arrhythmias with a Wide QRS Complex**

**3.2.8.1. Aberrant Conduction**

**3.2.8.2. PAC with Aberrant Conduction**

**3.2.8.3. Supraventricular Tachycardia (SVT) with Aberrant Conduction**

**3.2.8.4. Antidromic AV Reentrant Tachycardia**

4. AV Conduction

4.1. Defining a Normal AV Conduction

**4.1.1. Delayed AV Conduction**

**4.1.1.1. First-degree AV Block**

**4.1.1.2. Type I Second-degree AV Block (Mobitz I)**

**4.1.1.3. Type II Second-degree AV Block (Mobitz II)**

**4.1.1.4. 2:1 Second-degree AV Block**

**4.1.1.5. Advanced or High-grade Second-degree AV Block**

**4.1.1.6. Third-degree or Complete AV Block**

**4.1.1.7. Paroxysmal AV Block**

**4.1.2. Ventricular Pre-excitation**

**4.1.3. Other Mechanisms of Changes in the AV Relationship**

**(normal AV node conduction)**

**4.1.3.1. AV Dissociation**

**4.1.3.2. Retrograde Atrial Activation**

5. Analysis of Ventricular Activation

5.1. Normal Ventricular Activation

**5.1.1. Definition of a Normal QRS**

**5.1.2. Normal Electrical Axis in the Frontal Plane**

**5.1.3. Normal Ventricular Activation in the Transversal Plane**

**5.1.4. Analysis of Ventricular Rhythm Alterations**

**5.1.4.1. Definition of Cardiac Arrhythmia**

**5.1.4.2. Ventricular Arrhythmia**

**5.1.4.3. Analysis of Ventricular Arrhythmias**

**5.1.4.3.1. Premature Ventricular Complex (PVC)**

**5.1.4.3.2. Ventricular Escape Beat**

**5.1.4.3.3. Ventricular Escape Rhythm – Idioventricular Rhythm**

**5.1.4.3.4. Accelerated Idioventricular Rhythm**

**5.1.4.3.5. Ventricular Tachycardia (VT)**

**5.1.4.3.5.1. Monomorphic VT**

**5.1.4.3.5.2. Polymorphic VT**

**5.1.4.3.5.3. Torsades des Pointes (TdP)**

**5.1.4.3.5.4. Bidirectional VT**

**5.1.4.3.5.5. VT Length**

**5.1.4.3.6. Fusion Beat**

**5.1.4.3.7. Supraventricular Capture Beat During Idioventricular Rhythm**

**5.1.4.3.8. Ventricular Parasystole**

**5.1.4.3.9. Ventricular Fibrillation (VF)**

**5.1.4.4. Criteria for Differentiating Wide QRS Complex Tachycardias**

6. Cardiac Chambers hypertrophy

6.1. Atrial hypertrophy

**6.1.1. Left Atrial Hypertrophy**

**6.1.2. Right Atrial Hypertrophy**

**6.1.3. Biatrial hypertrophy**

**6.1.4. Left Ventricular Hypertrophy**

**6.1.4.1. Romhilt-Estes Criteria**

**6.1.4.2. Sokolow-Lyon Index**

**6.1.4.3. Cornell Index**

**6.1.4.4. Peguero-Lo Presti**

**6.1.4.5. Changes in Ventricular Repolarization**

**6.1.5. Right Ventricular Hypertrophy**

**6.1.5.1. QRS Axis**

**6.1.5.2. Tall R Wave**

**6.1.5.3. qR or qRs Morphology**

**6.1.5.4. rsR’ Morphology**

**6.1.5.5. Ventricular Repolarization**

**6.1.5.6. Seattle Criteria for RVH**

**6.1.6. Biventricular Hypertrophy**

**6.1.7. Differential Diagnoses for Increased QRS Amplitude**

7. Analysis of Intraventricular Blocks (Conduction Delay)

7.1. Intraventricular Blocks^74 , 75^

**7.1.1. Left Bundle-Branch Block (LBBB)**

**7.1.1.1. LBBB in Association with LVH**

**7.1.1.2. LBBB in Association with RVH (at Least 2 out of 3 Criteria)**

**7.1.2. Right Bundle-Branch Block (RBBB)**

**7.1.2.1. End Conduction Delay**

**7.1.3. Left Fascicular Blocks**

**7.1.3.1 Left Anterosuperior Fascicular Block (LAFB)**

**7.1.3.2. Left Anteromedial Fascicular Block**

**7.1.3.3 Left Posteroinferior Fascicular Block (LPFB)**

**7.1.4. Right Fascicular Blocks**

**7.1.4.1. Right Superior Fascicular Block**

**7.1.4.2. Right Inferior Fascicular Block**

**7.1.5. Bundle-Branch and Fascicular Blocks Association**

**7.1.5.1. LBBB in Association with LAFB**

**7.1.5.2. LBBB in Association with LPFB**

**7.1.5.3. RBBB in Association with LAFB**

**7.1.5.4. RBBB in Association with LPFB**

**7.1.5.5. RBBB in Association with LAFB and Left Anteromedial**

**Fascicular Block**

**7.1.5.6. LAFB in Association with Left Anteromedial Fascicular Block**

**7.1.5.7. Masquerading Bundle-Branch Block**

**7.1.6. Special Situations Involving Intraventricular Conduction**

**7.1.6.1. Peri-infarction Conduction Block**

**7.1.6.2. Peri-ischemia Block**

**7.1.6.3. QRS Complex Fragmentation (fQRS)**

**7.1.6.4. Atypical LBBB**

**7.1.6.5 Parietal or Purkinje/Muscle or Focal Intraventricular Block**

8. Analysis of the ECG In Coronary Heart Disease

8.1. Diagnostic Criteria for Myocardial Ischemia

**8.1.1. Presence of Ischemia**

**8.1.2. Circumferential or Global Ischemia**

**8.1.3. Secondary Changes**

8.2. Subendocardial and Subepicardial Injury

Diagnostic Criteria

8.3. Definition of Myocardial Fibrosis

8.4. Topographic Analysis of Ischemia, Injury, and Necrosis

**8.4.1. ECG Topographic Analysis of Ischemic Manifestations (Meyers)**

**8.4.2. ECG Topographic Analysis of Ischemic Manifestations in**

**Association with Magnetic Resonance Imaging**

**8.4.3. Electrocardiographic Correlation with the**

**Culprit Artery**

8.5. Particular Areas of Infarction

**8.5.1. Right Ventricle Myocardial Infarction**

**8.5.2. Atrial Infarction**

8.6. Differential Diagnoses

**8.6.1. Subepicardial Ischemia**

**8.6.2. Acute Myocardial Infarction (AMI) with ST-segment Elevation**

8.7. Association of Myocardial Infarction with Bundle-Branch Blocks

**8.7.1. Myocardial Infarction in the Presence of RBBB**

**8.7.2. Myocardial Infarction in the Presence of LBBB**

9. Analysis of Ventricular Repolarization

9.1. Ventricular Repolarization

**9.1.1. Normal Ventricular Repolarization**

**9.1.1.1. J-point**

**9.1.1.2. ST-Segment**

**9.1.1.3. T Wave**

**9.1.1.4. U Wave**

**9.1.1.5. QT interval and Corrected QT Interval (QTc)**

**9.1.2. Variants of Ventricular Repolarization**

**9.1.2.1. Early Repolarization Pattern**

10. Channelopathies and Other Genetic Alterations

10.1. Genetics and the ECG

**10.1.1. Channelopathies**

**10.1.1.1. Congenital Long QT Syndrome**

**10.1.1.2. Short QT Syndrome**

**10.1.1.3. Brugada Syndrome**

**10.1.1.4. Catecholaminergic Tachycardia**

**10.1.2. Genetic Diseases with Primary Cardiac Involvement**

**10.1.2.1. Arrhythmogenic Right Ventricular Cardiomyopathy**

**(Dysplasia)**

**10.1.2.2. Hypertrophic Cardiomyopathy**

**10.1.3. Genetic Diseases with Secondary Cardiac Involvement**

**10.1.3.1. Muscular Dystrophy**

11. Electrocardiographic Pattern in Specific

Clinical Situations

11.1. Clinical Conditions that Can Modify the ECG

**11.1.1. Digitalis Action**

**11.1.2. Drug-induced ST-T Changes**

**11.1.3. Electrical Alternans**

**11.1.4. T-wave Alternans (TWA)**

**11.1.5. Acute Injury of the Central Nervous System**

**11.1.6. Interatrial Communication**

**11.1.7. COVID-19**

**11.1.8. Pericardial Effusion**

**11.1.9. Dextrocardia**

**11.1.10. Dextroposition**

**11.1.11. Electrolyte Imbalance**

**11.1.11.1. Hyperkalemia**

**11.1.11.2. Hypokalemia**

**11.1.11.3. Hypocalcemia**

**11.1.11.4. Hypercalcemia**

**11.1.12. Chronic Obstructive Pulmonary Disease (COPD)**

**11.1.13. Antiarrhythmic Drugs**

**11.1.13.1. Amiodarone**

**11.1.13.2. Propafenone**

**11.1.13.3. Sotalol**

**11.1.14. Dielectric Effect**

**11.1.15. Pulmonary Embolism**

**11.1.16. Ashman or Gounaux-Ashman Phenomenon** ^17^

**11.1.17. Hypothermia**

**11.1.18. Hypothyroidism**

**11.1.19. Chronic Renal Failure**

**11.1.20. Pericarditis**

**11.1.21. Chemotherapy Drugs**

12. The ECG in Athletes

12.1. The Importance of Understanding the Athlete’s ECG

**12.1.1. Normal ECG Findings (Group 1)**

**12.1.2. Abnormal ECG Findings (Group 2)**

**12.1.3. Borderline ECG Findings (Group 3)**

13. The ECG in Children

13.1. Introduction

13.2. Technical Aspects

13.3. Electrocardiographic Parameters and their Variations

**13.3.1. Heart Rate and Sinus Rhythm**

**13.3.1.1. Possible Alterations**

**13.3.1.1.1. Sinus Arrhythmia**

**13.3.1.1.2. Sinus Tachycardia**

**13.3.1.1.3. Sinus Bradycardia**

**13.3.1.1.4. Other Bradycardias**

**13.3.2. P wave and Atrial Electrical Activity**

**13.3.2.1. Possible Alterations**

**13.3.2.1.1. Atrial Hypertrophy**

**13.3.2.1.2. Junctional Rhythm**

**13.3.3. PR Interval and AV Conduction**

**13.3.3.1. Possible Alterations**

**13.3.3.1.1. AV Blocks**

**13.3.3.1.2. Short PR Interval and Ventricular Pre-excitation**

**13.3.4. Ventricular Electrical Activity**

**13.3.4.1. Possible Alterations**

**13.3.4.1.1. Changes in the QRS Axis and Amplitude**

1 **3.3.4.1.2. Q-wave Alterations**

**13.3.4.1.3. Intraventricular Conduction Disturbances**

**13.3.4.1.4. Epsilon Wave and Arrhythmogenic Right Ventricular**

**Cardiomyopathy (Dysplasia)**

**13.3.5. Ventricular Repolarization**

**13.3.5.1. QT Interval**

**13.3.5.1.1. Possible Alterations**

**13.3.5.1.1.1. Long QT Syndrome**

**13.3.5.1.1.2. Short QT Syndrome**

**13.3.5.2. ST-Segment**

**13.3.5.2.1. Possible Alterations**

**13.3.5.2.1.1. ST-Segment Deviations**

**13.3.5.2.1.2. Early Repolarization**

**13.3.5.2.1.3. Brugada Electrocardiographic Pattern**

**13.3.5.3. T Wave**

**13.3.5.4. U Wave**

13.4. Heart Rhythm Disorders

13.5. Identification of Situs, Cardiac Position, and Ventricular Inversion

14. The ECG during Cardiac Pacing

14.1. Cardiac Pacing

**14.1.1. Basic Terms**

**14.1.2. Analysis of the Electrocardiographic Characteristics of CIED**

15. Tele-electrocardiography

References

## Introduction

This review of electrocardiography guidelines is a result of advances in the understanding of various diseases, with important repercussions to the electrocardiographic tracing. Some professionals may imagine that electrocardiogram (ECG) interpretation has not gone through changes with time; they certainly ignore recently described diseases and other health problems whose electrophysiological mechanisms are better understood now than in the past. Some electrocardiographic parameters are important prognostic markers in Chagas disease, and changes considered as predictors of mortality can be identified in the general population (ECG-age). A crucial question is: when to recommend an ECG?

The ECG is a simple, low cost, non-invasive exam that provides an idea of an individual’s cardiac condition and can eventually identify situations with risk of sudden death. Therefore, ECG findings within normal limits may anticipate that the ventricular function should be normal or close to normal, which is an important factor at first contact with the patient.

We believe that all persons should undergo an ECG at some point in their lives, and this examination should only be repeated in case of clinical need. Some guidelines state a class IIb indication for an ECG in asymptomatic individuals of the general population and a class IIa indication in case of hypertension and/or diabetes.^1^

The possibility of transmitting ECG tracings by the internet allowed the diffusion of this technology in various underprivileged regions of our country and the achievement of better standards of health assistance. In recent years, a significant increase (with millions of analyzed ECGs) has been seen in studies of artificial intelligence and automatic interpretation systems as additional tools for electrocardiography. Some results were able to demonstrate the ability of these new systems to identify some arrhythmias, as well as predict their appearance, in addition to anticipating outcomes such as ischemic stroke. Therefore, we expect that this update helps standardize the issuing of electrocardiographic reports by medical doctors allowing easier electrocardiographic understanding.

## 1. Standards for the Analysis and Issuance of Electrocardiographic Reports

### 1.1. Standards for Electrocardiographic Analysis

Three characteristics should be considered for an accurate electrocardiographic interpretation:

- Age: ECG characteristics depend on age and are clearly seen in age groups of newborns, infants, children, and adolescents up to 16 years of age. In the first 2 groups, these changes are faster (Section 13). Adults may also show negative T waves exclusively in V1.^2^
- Body type: slender individuals sometimes have their hearts in the upright (vertical) position, and the resulting axes, especially the P wave and QRS complex, are normally shifted to the right with a clockwise rotation in the frontal plane leads. On the other hand, in short and broad individuals with hearts in horizontal position, deviations are usually to the left (frontal plane).
- Sex: In female adults, negative T waves are commonly observed in the right precordial leads, with a larger QTc than male individuals and children.

### 1.2. The Electrocardiographic Report 1 , 3 - 5

#### 1.2.1. Descriptive Report

- Analysis of the rhythm and quantification of the heart rate (HR);
- Analysis of the duration, amplitude, and morphology of the P wave, and duration of the PR interval;
- Electrical axis determination: P wave, QRS complex, and T wave;
- Analysis of the duration, amplitude, and morphology of the QRS complex;
- Analysis of ventricular repolarization and description of ST-segment, T wave, QT, and U changes, when present.

#### 1.2.2. Final Report

It should contain the synthesis of diagnoses listed in these guidelines. Abbreviations can be used in reports, scientific texts, and protocols, among other documents, between parentheses and after the standard diagnostic definition.

#### 1.2.3. Automated Report

In recent years, technological development has brought significant improvements to the accuracy of automatic measurements made by the currently available equipment, making automated interpretation an important auxiliary tool for the medical report. However, the verification of automatic measurements by a medical doctor is of paramount importance. The simple use of automatic metric and vector measurements, as well as reports issued by these systems, if not revised, are not recommended.

#### 1.2.4. Reports Via the Internet

Tele-ECG systems^6 - 8^send ECGs performed remotely to referral centers for report issuance. The technique for performing ECGs (by the performing units), as well as the interpretation and reports (by the referral centers), should follow the most recent national and international guidelines. These are a part of telecardiology, which also comprehends other examinations in this specialty that are performed, recorded, and transmitted from one site to another for remote interpretation. Some examples include the monitoring of pacemakers, Holter monitors, and the cardiac event recorder. Among the numerous benefits of telecardiology, we cite:

- Pre-hospital care at the patient’s own location;
- Reductions in time and costs for the patient;
- Faster triage by specialists;
- Access to specialists in case of accidents and emergencies;
- Facilitated management of health care resources;
- Increased safety of post-surgical patients during rehabilitation;
- Cooperation and integration between researchers for sharing clinical records;
- ccess to educational programs of training and qualification.

According to various authors, telecardiology is a socially and economically advantageous activity for service providers, sponsors, and patients. It is recognized as a useful tool in locations that are distant from large city centers.

## 2. Tracing Technical Quality Analysis

### 2.1. Tracing Technical Evaluation Criteria

#### 2.1.1. Calibration of the Electrocardiograph

In analogic equipment, calibration should always be verified. The normal pattern must have 1 mV (10 mm). On the other hand, in modern computerized equipment with digitized tracing, the calibrator pattern is verified automatically. Digital filters should follow internationally accepted recommendations, especially those from the AHA. For adults and adolescents, high-frequency filter cutoffs of at least 150 Hz should be used. For children, filter cutoffs of up to 250 Hz. Filters with lower frequencies can interfere when capturing pacemaker spikes. Low-frequency filters should use a 0.05 Hz cutoff. Some equipment use bidirectional filters.^9^

#### 2.1.2. Lead Reversal

Figure 2.1 shows the correct position of peripheral electrodes — right arm (RA), left arm (LA), right leg (RL), and left leg (LL) — with their respective colors (red, yellow, black, and green).

**Figure 2.1:** Positioning of peripheral electrodes.

RA: right arm; LA: left arm; RL: right leg; LL: left leg.

##### 2.1.2.1. Lead Switches

**2.1.2.1.1. Transposition of Upper Limb Electrodes**

DI leads with negative waves and aVR with positive waves.

##### 2.1.2.2. Lower Limb Electrode Swapped for Upper Limb Electrodes

Isoelectric line or very small wave amplitudes in D2 (right arm) or D3 (left arm). Swapping upper limb electrodes with lower limb electrodes shows this pattern in D1 due to the negligible potential difference in the upper limbs.

##### 2.1.2.3. Left Arm Electrode Swapped for Left Leg Electrode

This is the most difficult lead reversal to detect. The QRS axis tends to shift to the left. It may look like a normal ECG, but it produces the following alterations:

- Inverted P wave in D3;
- Positions of D1 and D2 are changed; QRS voltage is higher in D1 and lower in D2;
- In D3, P, QRS, and T are inverted. Positions of aVL and aVF are also changed. The aVR lead does not change.

##### 2.1.2.4. Precordial Electrode Reversals

The normal progression of the R wave from V1 to V6 is changed.

##### 2.1.2.5. Misplacement of the V1 and V2 Electrodes

V1 and V2 electrodes incorrectly positioned above the second intercostal space may produce an rSr’ pattern simulating end conduction delay (or an IRBBB pattern), or an rS morphology from V1 to V3 and a negative P wave in V1, simulating left atrial hypertrophy.

## 2.1.3. Other Interferences

### 2.1.3.1. Muscle Tremors

Muscle tremors may interfere with the baseline, mimicking electrocardiographic changes such as atrial flutter and ventricular fibrillation^10^in patients with Parkinson’s disease.

### 2.1.3.2. Neurostimulation

Patients with central nervous system disorders who require electrical stimulation devices can present artifacts that mimic cardiac pacemaker spikes.

### 2.1.3.3. Cold, Fever, Hiccups, and Psychomotor Agitation

These conditions produce baseline artifacts and can mimic arrhythmias such as atrial fibrillation and atrial flutter.

### 2.1.3.4. “Large Precordial Electrode”

The use of conductive gel as a continuous strip on the precordium results in similar tracings in V1–V6, corresponding to the mean electric potential in these leads.^3^

### 2.1.3.5. Baseline Wander

It may be caused by loose electrodes, movement of the limbs, breathing, or when the patient is in a wheelchair. When the patient is in a wheelchair, other artifacts can also be recorded.

### 2.1.3.6. Other Electrical and Electromagnetic Interferences

These result from interferences from electrical lines or equipment and cell phones. Prior to performing the ECG, the patient should be requested to put away all metallic objects and cell phones. Transcutaneous pacemakers can produce spikes, which could be mistaken for a false capture. The filter used in the ECG is also important since it sometimes creates a false capture failure, which generates a pause represented by an isoelectric line between 2 beats.^11 - 12^

### 2.1.3.7. Alterations Due to the Malfunction of Software and Computerized Electrocardiographic Signal Acquisition Systems

Data acquisition by computerized systems, in some older electrocardiographs, can seldom present specific problems that are not yet completely understood. In the absence of an electrocardiographic signal in one of the electrodes, for example, the system may counterbalance the other acquired signals and create bizarre QRS complexes. Twelve-lead simultaneous equipment, which automatically measure P wave and QRS durations, can overestimate these measurements because the software uses the first and the last wave (among the 12 leads) for generating these values.

## 3. Heart Rhythm Analysis

### 3.1. Analysis of the P Wave, HR, and Rhythm

Population-based studies on normal electrocardiographic ranges have been used for many years as reference for our population, even though it is known that ethnic differences influence what is considered normal. In 2017, among all the information obtained by the ELSA-Brasil study, a research paper on the normal values for the Brazilian population without heart disease was published.^13^

The parameters addressed on Section 3 refer to adult ECGs. Pediatric ECGs will be addressed on Section 13.

#### 3.1.1. Definition of Sinus Rhythm

This is the physiological rhythm of the heart, originating from the upper right atrium and observed on the surface ECG as positive P waves in the D1, D2, and aVF leads regardless of the presence of a QRS complex. The P axis may vary from 0º to +90º. The maximum amplitude and duration of the normal P wave are 2.5 mm and 110 ms, respectively. Changes in its morphology may happen depending on the HR and orientation (P-wave axis) in the observed leads.^14^

#### 3.1.2. Frequency of the Sinus P Wave

The normal HR during waking hours ranges from 50 bpm to 99 bpm.^14 - 16^

## 3.2. Analysis of Supraventricular Rhythm Alterations

### 3.2.1. Definition of Cardiac Arrhythmia

Cardiac arrhythmia is due to abnormality of formation and/or conduction of the electrical impulse across the myocardium.^17^After the definition (or not) of the presence of sinus rhythm, cardiac arrhythmia is investigated.

### 3.2.2. Supraventricular Arrhythmia

This rhythm originates above the bundle of His. The site of origin of this arrhythmia should be identified whenever possible. When this is not possible, the generic term “supraventricular” will be used.

### 3.2.3. Presence of Sinus P Wave

#### 3.2.3.1. Sinus Arrhythmia

It is usually physiologic and depends on the autonomic nervous system, being characterized by a variation in PP intervals between 160 ms and 220 ms during sinus rhythm. Phasic variations are related to breathing (frequently seen in children), as opposed to nonphasic variations.

#### 3.2.3.2. Sinus Bradycardia

Sinus bradycardia is a sinus rhythm with HR below 50 bpm.

#### 3.2.3.3. Second-degree Sinoatrial Block

The second-degree exit block of sinus depolarization results in a lack of P wave in a cycle. Type I sinoatrial block is characterized by progressively shorter PP cycles before the block. Type II sinoatrial block shows no differences between PP cycles, and the pause corresponds to 2 previous PP cycles. Sinoatrial block I is not visible on a standard ECG. Third-degree blocks are seen as atrial or junctional escape rhythm.

#### 3.2.3.4. Interatrial Blocks (IAB)

These are conduction delays between the right and left atria, which can be classified as first-degree (P wave duration of 120 ms or longer), second-degree (these patterns are transitory), and third-degree or advanced (P wave duration of 120 ms or longer, biphasic or plus-minus in the inferior wall, related with supraventricular arrhythmias and Bayés syndrome).^18 , 19^

#### 3.2.3.5. Sinus Tachycardia

Sinus Tachycardia is a sinus rhythm with a HR of 100 bpm or higher.

## 3.2.4. Absence of P Wave before the QRS

### 3.2.4.1. Atrial Fibrillation (AFib)

Disorganized atrial electrical activity, with rates ranging from 450 to 700 beats/min and a variable ventricular response. The baseline can be isoelectric, with fine or coarse irregularities, or have a combination of these changes (f-waves). Regular RR intervals indicate atrioventricular (AV) dissociation. The ventricular response of an AFib can be calculated from a 6-s tracing (number of QRS complexes in this period and multiplied by 10). We then have the following possibilities of ventricular response (during resting ECG):

1. AFib rhythm with slow ventricular response, when HR is ≤ 50 bpm;
2. AFib rhythm with adequate HR control, when ventricular response is between 60 bpm and 80 bpm;
3. AFib rhythm with inadequate HR control, when ventricular response is between 90 bpm and 110 bpm;
4. AFib rhythm with rapid ventricular response, when HR is > 110 bpm.

### 3.2.4.2. Atrial Flutter

Atrial flutter is an oganized atrial electrical activity (macroreentrant mechanism) across a large area of the right atrium. It is named typical atrial flutter when it runs through (and is dependent on) the cavotricuspid isthmus (CTI). Macroreentry can occur both in the counterclockwise (90% of the cases) or clockwise (10%) directions. In the former, it is named typical atrial flutter and in the latter, it is named REVERSE typical atrial flutter. In a typical atrial flutter, the known F waves have rates of 240 bpm to 340 bpm and a characteristic pattern: a sawtooth aspect, being negative in the inferior leads and generally positive in V1. Varying degrees of AV conduction may occur, and when higher than 2:1, the detection of negative F waves is easier. On the other hand, F waves have higher rates in the reverse atrial flutter (between 340 bpm and 430 bpm). F waves are positive in the inferior leads, and also widened. When it comes to the ECG, it is not possible to differentiate between a REVERSE typical atrial flutter and left atrial tachycardia (originating from the right superior pulmonary vein). The so-called atypical atrial flutter does not go through the CTI. Therefore, this classification includes scar-related atrial tachycardias, atrial tachycardias arising from the inferior vena cava, and reentrant tachycardias originating from the mitral valve annulus, which are all very difficult to diagnose on the ECG (receiving the generic name of atrial tachycardia).

### 3.2.4.3. Junctional Rhythm

Junctional rhythm is an escape rhythm originating from the AV junction, with QRS which are similar or slightly different from sinus rhythm. This aberrancy is due to a different origin of the stimulus and not to phasic aberrant conduction, which depends on the stimulus being altered by phase 3 (early) or 4 (late) of the action potential. It can happen with no visible P wave on ECG. These “positions” of the P wave are due to the conduction velocities of electrical impulses to the atria and ventricles. By reaching the ventricles first and the atria second, the P wave is located within or after the QRS complex. Junctional escape rhythm is defined when HR is < 50 bpm. Active junctional rhythm is determined when HR > 50 bpm. Finally, tachycardia is defined if HR > 100 bpm.

### 3.2.4.4. Junctional Extrasystole

Junctional extrasystole is an early ectopic beat originating from the AV junction. There are 3 possible electrocardiographic presentations for this phenomenon:

- Negative P wave in the inferior leads with a short PR interval;
- Lack of atrial activity preceding the QRS complex (P wave buried within the QRS);
- Negative P wave in the inferior leads after the QRS complex;

The QRS complex has similar morphology and duration to the baseline rhythm, although aberrant conduction may occur (see Items 3.2.8.1 and 3.2.8.2).

### 3.2.4.5. Common atrioventricular nodal reentrant tachycardia (AVNRT) ^20^

This type of tachycardia happens within the AV node, and nodal reentry is its electrophysiological mechanism. In ninety percent, one circuit uses a fast pathway (retrograde) and the other uses a slow pathway (anterograde) and it is called common AVNRT. When the QRS is narrow during tachycardia, pseudo S waves can be seen in the inferior leads and an rSr’ (pseudo r’) morphology can be seen in V1, reflecting atrial activation from AV node to sinus node direction. This retrograde atrial activation, in most cases, occurs within 80 ms of QRS onset (RP < 80 ms). Sometimes, the atrial activation wave is buried within the QRS and is thus not seen on the ECG. There are some similarities between common AVNRT and orthodromic AV reciprocating tachycardia (AVRT). RP interval is used to distinguish them and it will be described next. In cases of common AVNRT with a wide QRS, differential diagnoses must consider monomorphic ventricular tachycardia.

### 3.2.4.6. Orthodromic AV Reciprocating Tachycardia (AVRT)

This type of reentrant tachycardia uses the normal conduction system in the anterograde direction and an accessory pathway in the retrograde direction. In general, the QRS is narrow and the P wave is retrograde, being more commonly located in the ST-segment. The P wave can present diverse morphologies, according to the location of the accessory pathway. The RP interval is > 80 ms.

## 3.2.5. Occurrence of a Non-sinus P Wave Before the QRS Complex

### 3.2.5.1. Ectopic Atrial Rhythm

Ectopic atrial rhythm corresponds to atrial activity occurring in a different location from the anatomic region of the sinus node. Thus, the P wave has a different morphology (polarity) from that characterizing sinus rhythm.

### 3.2.5.2. Multifocal Atrial Rhythm

It is originated from multiple atrial foci, with an HR < 100 bpm, recognized on the ECG by the presence of at least 3 different P wave morphologies and 3 different PR intervals. PP and PR intervals are frequently variable, and one P wave is seen for each QRS complex; blocked P waves may occur.

### 3.2.5.3. Junctional Rhythm

As mentioned on Item 3.2.4.3, it is characterized by negative P waves in the D2, D3, and aVF leads, in addition to a short PR interval. Junctional escape rhythm is defined when HR < 50 bpm. Active junctional rhythm is determined when HR is > 50 bpm; junctional tachycardia is defined when HR > 100 bpm.

### 3.2.5.4. Delayed Atrial Beat

A delayed atrial beat can be considered a “replacement” atrial beat. It is frequently seen when a temporary interruption of normal sinus automaticity occurs as a consequence of sinus node inhibition / failure. It can be from right or left atrium, usually late, and it has a P wave of non-sinus morphology.

### 3.2.5.5. Premature Atrial Complex (PAC)

Premature atrial complex is an early atrial ectopic beat. It may recycle the baseline PP interval.

### 3.2.5.6. Blocked or Non-conducted PAC

Ectopic beat that originates from the atrium sometimes cannot be conducted to the ventricles, thus a premature P wave without a QRS complex can be seen. There are two main causes for the lack of conduction: a very early premature atrial complex that reaches the AV node within its absolute refractory period, or a previous His-Purkinje conduction system disease. PACs not conducted to ventricles may lead to bradycardia.

### 3.2.5.7. Atrial Tachycardia

Atrial tachycardia is an atrial rhythm that originates from a region other than the sinus node (characterized by a P wave of a different morphology) with an atrial rate > 100 bpm. Variable AV conduction is common.

### 3.2.5.8. Multifocal Atrial Tachycardia

It has the same characteristics of multifocal atrial rhythm with an atrial rate > 100 bpm.

### 3.2.5.9. Uncommon AV Nodal Reentrant Tachycardia (AVNRT)

Its location is exactly the same to common AVNRT (3.2.4.5), but the circuit activation happens in the reverse direction (10%). Ventricular activation occurs through the fast pathway (anterograde) and the atrial activation occurs through the slow pathway (retrograde) and it is called uncommon AVNRT; this is why retrograde atrial activation happens later, with a characteristic longer RP interval than PR. Therefore, uncommon AVNRT is not a differential diagnosis for common AVNRT or orthodromic AVRT.

### 3.2.5.10. Permanent Junctional Reciprocating Tachycardia (Coumel Tachycardia)

Permanent junctional reciprocating tachycardia is a supraventricular tachycardia that uses a particular accessory pathway (with an exclusive and decremental retrograde conduction). It is characterized by tachycardia with a long RP interval and its differential diagnoses include those described on Items 3.2.5.7 and 3.2.5.9.

## 3.2.6. Pauses

Pauses are defined by a lack of P wave and QRS complex with an interval > 1.5 s. Clinical significance is considered when longer than 2 s. The occurrence of pauses may be related to sinus arrest, non-conducted PAC, sinoatrial block, and AV block.

### 3.2.6.1. Sinus Arrest

Sinus arrest corresponds to a pause in sinus activity > 1.5 times the basic PP cycle.

### 3.2.6.2. Sinus Node Dysfunction

The inability of the sinus node to maintain HR above the physiological need for the present situation is named sinus node dysfunction. On the ECG, this abnormality (or dysfunction) encompasses sinus pause, sinoatrial block, sinus bradycardia, replacement rhythms, AFib, atrial flutter, and tachy-brady syndrome, among other disorders.^21^

## 3.2.7. Classification of Supraventricular Tachycardias Based on the RP Interval

The RP interval is a commonly used measure for characterizing supraventricular tachycardia. Its measurement is done from the beginning of the QRS complex to the following P wave (RP). Depending on the position of this P wave, the RP interval can be short (P wave before the midpoint of 2 consecutive QRS) or long (P wave located after the midpoint of 2 QRS). Therefore, paroxysmal supraventricular tachycardias can be divided into:

- Short RP (normally up to 120–140 ms), as observed in common AVNRT and orthodromic AVRT;
- Long RP, as observed in atrial tachycardia, uncommon AVNRT, and Coumel tachycardia (permanent junctional reciprocating tachycardia).^22^

## 3.2.8. Supraventricular Arrhythmias with a Wide QRS Complex

### 3.2.8.1. Aberrant Conduction

Supraventricular stimulus with hampered propagation in the conduction system, generating a QRS complex of different morphology when compared to the baseline QRS complex; it may resemble a bundle-branch block pattern, a fascicular block pattern, or both.

### 3.2.8.2. PAC with Aberrant Conduction

PAC with aberrant conduction is an early P wave followed by a QRS complex with a bundle-branch block pattern or fascicular block pattern, or both.

### 3.2.8.3. Supraventricular Tachycardia (SVT) with Aberrant Conduction

It is a generic denomination for the aforementioned tachycardias presenting with aberrant conduction.

### 3.2.8.4. Antidromic AV Reentrant Tachycardia

Reentrant tachycardia uses an accessory pathway in an anterograde direction and the conduction system in a retrograde direction. The aberrant QRS is characterized by the presence of ventricular pre-excitation. Differential diagnoses must consider ventricular tachycardia. The presence of 1:1 retrograde atrial depolarization favors accessory pathway conduction involvement and AV dissociation is diagnostic of ventricular tachycardia.

## 4. AV Conduction

### 4.1. Defining a Normal AV Conduction

The period from the beginning of the P wave until the beginning of the QRS complex determines the PR interval, when there is atrial activation and physiological delay in the AV junction and/or the His-Purkinje system. Its duration is 120–200 ms, considering a maximum HR of 90 bpm. The PR interval varies according to HR and age.

#### 4.1.1. Delayed AV Conduction 23 - 26

Before studying them it is essential to remember the normal decremental conduction related to the AV node, which is an important electrophysiological characteristic of the AV node. This property refers to a reduction in the conduction velocity of the electrical impulse in the AV node, and it can be estimated through the PR interval on conventional ECG. This interval is considered normal in adults when between 120 ms and 200 ms, depending on age and HR.

Delayed AV conduction occurs when atrial impulses have a delay or fail to reach the ventricles.

Anatomically, abnormal AV node delayed conduction can be located in the AV node itself (nodal block), in the His-Purkinje bundle (intra-His block), or below this structure (infra-His block). Nodal conduction delays normally present narrow QRS complexes (< 120 ms) and have a good prognosis; these are expressed by an increased PR interval. On the other hand, intra- and infra-His delays usually have wide QRS complexes and worse disease progression. In these cases, a normal PR interval is uncommon.

We highlight that the AV node is greatly influenced by the autonomic nervous system; therefore, in situations where parasympathetic tone prevails (during sleep, in athletes), first-degree AV block and/or type I second-degree AV block may be seen, even without AV node lesion.

##### 4.1.1.1. First-degree AV Block

In this case, the PR interval is > 200 ms in adults, for an HR between 50 bpm and 90 bpm.

##### 4.1.1.2. Type I Second-degree AV Block (Mobitz I)

In this case, AV conduction gradually slows down (Wenckebach phenomenon). Typically, there is a progressive increase in the PR interval; these increases are gradually shorter until AV conduction is blocked and a sinus beat cannot be conducted. Therefore, there is a gradual increase in the PR interval with simultaneous shortening of RR intervals until a P wave is blocked. This cycle may be repeated for variable periods, where the PR interval immediately after the blocked beat should be the smallest, and the next interval should have the largest proportional increase when compared to the ones that follow. The ratios for this block may vary such as 5:4; 4:3; 3:2 conduction.

##### 4.1.1.3. Type II Second-degree AV Block (Mobitz II)

An abrupt failure of AV conduction happens in this situation. There is 1:1 AV conduction with a fixed PR interval, then a sudden P wave is blocked followed by new 1:1 AV conduction with a similar PR interval to the previous ones. The block is located at an intra/infra-His Purkinje site.

**4.1.1.4. 2:1 Second-degree AV Block**

It is characterized by alternating conducted and blocked sinus P waves. Most of 2:1 AV blocks are located at an intra/infra His-Purkinje site. The diagnosis of non-conducted PAC should be excluded.

##### 4.1.1.5. Advanced or High-grade Second-degree AV Block

AV conduction happens in less than 50% of all sinus beats in a 3:1 ratio, 4:1 ratio, or higher. In general, AV conduction is noticed by a constant PR interval for each beat conducted. Most of them are located at an intra/infra His-Purkinje site. Junctional escapes may occur.

##### 4.1.1.6. Third-degree or Complete AV Block

Stimuli of sinus origin are unable to reach and depolarize the ventricles; a focus below the blocked region, therefore takes ventricular rhythm control. As a result, there is no relationship between atrial and ventricular electrical activities (AV dissociation), which is translated on the ECG as P waves unrelated with QRS complexes. The rate of the sinus rhythm is higher than that of the ventricular escape rhythm. Third-degree AV block may be intermittent or permanent. AV Blocks originating from the supra-Hisian region may present escapes that resemble the baseline ECG. On the other hand, an infra-Hisian origin shows large QRS complexes.

##### 4.1.1.7. Paroxysmal AV Block

Paroxysmal AV block is a series of a sudden, consecutive blocked P waves.

## 4.1.2. Ventricular Pre-excitation 27 - 30

In patients with ventricular pre-excitation, muscle fibers remain within the fibrous tissue and act as accessory pathways for conducting the electrical impulse between the atria and ventricles. These additional pathways may be located in any part of the AV annulus ( Figure 4.1 ). The classical pattern is characterized by a PR interval < 120 ms during sinus rhythm in adults and < 90 ms in children (varying with age and HR); a slurring (delta wave) on the initial portion of the QRS complex close to the P wave; QRS duration > 120 ms in adults and > 90 in children; and secondary changes in ST-segment and T wave. These ECG findings with the diagnosis of symptomatic paroxysmal SVT characterizes Wolff-Parkinson-White (WPW) syndrome. The accessory pathway can be anatomically located using 12-lead ECG. Left-sided pathways are the most common (50% of the cases), followed by posteroseptal pathways (25%), right-sided pathways (15%), and anteroseptal pathways (10%). Anterior regions of the AV annulus are superior; this way, local accessory pathways determine activation in the superoinferior direction, with a positive delta wave in the inferior leads. On the other hand, the basal posterior region is inferior, with a consequently negative delta wave in the inferior leads. Differential diagnosis should occasionally be performed with a short PR interval and no delta wave, which are present in Lown-Ganong-Levine syndrome,^31^and a normal PR interval with ventricular pre-excitation, which is present in fasciculoventricular pathways such as the Mahaim variant.^32^

**Figure 4.1:** Accessory pathway sites on the tricuspid and mitral valve annuli

Accessory pathways can be divided when the QRS complex is predominantly positive (R) in V1 and V2, which indicates a left accessory pathway. When the QRS complex is negative (QS or rS), the accessory pathway is located to the right. Left-sided accessory pathways appear on the ECG as a negative delta wave in the D1 and/or aVL leads, a positive delta wave in the D2, D3, and aVF leads, and in V1 and V2. Right-sided accessory pathways present a positive delta wave in D1, D2, and aVL leads, and a normally negative one in D3 and aVR leads, as well as V1. The frontal plane QRS axis is shifted leftwards. On the other hand, posteroseptal pathways show a negative delta wave in D2, D3, and aVF. The importance of recognizing the locations of anteroseptal and midseptal pathways concerns their proximity to the bundle of His, which is associated with a higher risk during catheter ablation. In both locations, the delta wave is positive in the D1, D2, and aVL leads, while it is negative in D3 and aVR and positive/isoelectric in aVF, with a normal QRS axis. In 80% of the cases, the R/S transition occurs in V2.^32^

The analysis of QRS complexes in V1 and V2 will define whether they are located to the right or to the left.^33^Several algorithms can be used to localize the accessory pathway based either on the polarity of the QRS complex or the accessory pathway.^34 - 36^It is important to note that diseases such as hypertrophic cardiomyopathy and familial forms of glycogen storage disease (Fabry disease) may mimic the presence of pre-excitation.

## 4.1.3. Other Mechanisms of Changes in the AV Relationship (normal AV node conduction)

### 4.1.3.1. AV Dissociation

AV dissociation is caused by the following mechanisms: replacement, interference, AV block, and arrhythmia.^37^Two dissociated rhythms take place, one is of atrial origin (usually a sinus rhythm with a regular PP interval), and the other is of junctional or ventricular origin, also with a regular RR interval. Both foci rates are similar (isorhythmic dissociation). Ventricular rhythm may be hyperautomatic.

### 4.1.3.2. Retrograde Atrial Activation

Retrograde atrial activation can be observed when the activation is originated from junctional or ventricle sites stimulation. There is a retrograde conduction, usually through the AV node or an accessory pathway. It is mandatory that a negative P wave after a QRS complex is found in the inferior leads.

## 5. Analysis of Ventricular Activation

### 5.1. Normal Ventricular Activation

#### 5.1.1. Definition of a Normal QRS

The QRS complex is considered normal when its duration is < 120 ms in all leads and its amplitude is 5–20 mm in the frontal plane leads and 10–30 mm in the precordial leads, with a normal orientation of the electrical axis.^38 , 39^

#### 5.1.2. Normal Electrical Axis in the Frontal Plane

The normal limits of the frontal plane QRS axis are normally -30º and +90º.

#### 5.1.3. Normal Ventricular Activation in the Transversal Plane

It is expected a smooth transition of the typical rS morphology in V1 to the qR pattern in V6. So, from V1 to V6 there is a progressive increase of the r wave and decrease of the S wave amplitudes. In general, intermediate RS pattern (transition zone) occurs in V3 or V4.^16^

#### 5.1.4. Analysis of Ventricular Rhythm Alterations

##### 5.1.4.1. Definition of Cardiac Arrhythmia

Cardiac arrhythmia can be defined as a change in frequency, formation, and/or conduction of the electrical impulse across the myocardium.^17^

##### 5.1.4.2. Ventricular Arrhythmia

Ventricular arrhythmia is an arrhythmia that originates below the bundle of His, usually seen with a wide QRS.

##### 5.1.4.3. Analysis of Ventricular Arrhythmias

**5.1.4.3.1. Premature Ventricular Complex (PVC)^40^**

PVC is a beat that originates in the ventricle before it is expected, in most cases with a postextrasystolic pause and recycling the RR interval. In the absence of a pause, it is named interpolated PVC. PVCs usually have a QRS > 120 ms. Exceptionally they can be < 120 ms (PVCs originating from the ventricular septum or close to the conduction system). Regarding their morphology, they can be monomorphic (the same morphology at the same lead) or polymorphic (two or more morphologies at the same lead). According to their recurrence, they can be classified as isolated, paired, bigeminal, trigeminal, quadrigeminal, or concealed.

**5.1.4.3.2. Ventricular Escape Beat**

Ventricular escape beat is characterized when ventricular depolarization occurs late. It appears due to the temporary inhibition of anatomically higher rhythms.

**5.1.4.3.3. Ventricular Escape Rhythm – Idioventricular Rhythm**

An idioventricular rhythm It originates in the ventricles, with a HR < 40 bpm, and replaces anatomically higher rhythms that were inhibited or blocked.

**5.1.4.3.4. Accelerated Idioventricular Rhythm**

An accelerated idioventricular rhythm originates in the ventricle (wide QRS), with HR > 40 bpm (50–130 bpm, more frequently 70–85 bpm) as a result of increased automaticity. It is not a subsidiary rhythm and competes with the baseline rhythm of the heart. It is usually self-limited and associated with ischemic myocardial disease (reperfusion/ischemia).^41^

**5.1.4.3.5. Ventricular Tachycardia (VT)**

VT is a cardiac rhythm that presents three or more successive beats at a rate > 100 bpm.

**5.1.4.3.5.1. Monomorphic VT**

Monomorphic VT is characterized by uniform QRS morphology in the same lead.

**5.1.4.3.5.2. Polymorphic VT**

Polymorphic VT is a fast ventricular rhythm with 3 or more different wide QRS morphologies.^42^

**5.1.4.3.5.3. Torsades des Pointes (TdP)**

TdP is a wide QRS polymorphic tachycardia with a QRS that “rotates” around the baseline (twisting motion). It is normally preceded by long-short cycles (sinus beat-PVC) and is due to a long QT interval in sinus rhythm, which can be congenital or secondary to medications, electrolyte imbalance, or certain heart diseases.^43^

**5.1.4.3.5.4. Bidirectional VT^44^**

Tachycardia of ventricular origin where the right bundle-branch (or rarely the left bundle-branch) is constantly blocked, while the anterosuperior and posteroinferior divisions of the left bundle-branch are blocked in an alternating mode, beat by beat. In the frontal plane, a beat with positive QRS is alternated with a beat with negative QRS, generating the bidirectional aspect. This type of arrhythmia is related with digitalis toxicity, severe myocardial disease due to advanced cardiomyopathy, and cases with no structural heart disease such as catecholaminergic polymorphic ventricular tachycardia; it usually precedes polymorphic VT.

**5.1.4.3.5.5. VT Length**

Sustained: a tachycardia that lasts > 30 s or is associated to symptoms of hemodynamic instability. Non-sustained: a tachycardia that lasts < 30 s and there is no symptoms of hemodynamic instability.

**5.1.4.3.6. Fusion Beat**

Fusion beat corresponds to a beat that is generated from two sites: an activation from the ventricle and another from the atria. Electrocardiographically, it presents a P wave followed by a wide QRS (a hybrid morphology between a supraventricular beat and a beat of ventricular origin). Fusion beats are seen in the following situations: ventricular pre-excitation, VT, parasystole, and some PVCs.

**5.1.4.3.7. Supraventricular Capture Beat During Idioventricular Rhythm**

This is a beat originating from the atrium that can overcome the anatomical or functional conduction block in the AV junction and completely or partially depolarize the ventricle; in case of partial depolarization, a fusion beat occurs.

**5.1.4.3.8. Ventricular Parasystole**

Ventricular parasystole corresponds to the beat that originates from a ventricular site and competes with the sinus rhythm (a parallel pacemaker with a permanent entry block and occasional exit block). It is electrocardiographically visible for its own rate, fusion beats, periods of interectopic intervals with a multiple denominator, and variable coupling intervals.^45^

**5.1.4.3.9. Ventricular Fibrillation (VF)**

VF is characterized by bizarre and chaotic waves, with variable amplitude and ventricular rate. It corresponds to one of the clinical presentations of cardiac arrest. It can be preceded by VT or TdP that degenerated into VF.

##### 5.1.4.4. Criteria for Differentiating Wide QRS Complex Tachycardias 46 - 57

Most wide QRS complex tachycardias (80%) are of ventricular origin, and the presence of structural heart disease reinforces this possibility. AV dissociation, fusion beats, and/or capture beats (with different QRS) strongly suggest a VT diagnosis. Some algorithms, such as those by Brugada and by Vereckei^48^(widely known) and others, help differentiate those wide QRS tachycardias ( Table 5.1 ).^49 - 54^Figures 5.1 and 5.2 show ECG features of Brugada and Steuer criteria for diagnosing VT.

**Table 5.1:** Electrocardiographic criteria for differentiating supraventricular tachycardia with aberrant conduction from ventricular tachycardia

Autor	Wellens^49^(1978)	Brugada^46^(1991)	Steuer^51^(1994)	Vereckei^54^(2008)	Pava^55^(2010)	Jastrzebski^56^or VT Escore (2016)	Santos Neto^57^(2021)
Findings and steps of analysis for each algorithm	AV dissociation	Absence of RS in the precordial leads	Predominantly negative QRS complexes from V4 to V6	Initial R wave in the aVR lead	Interval from the onset of the QRS complex to the apex of the R wave ≥ 50 ms in the D2 lead	Dominant R wave in the V1 lead	Predominantly negative polarity in 4 leads: DI, D2, V1, and V6
	QRS complex > 140 ms (RBBB)	RS ≥ 100 ms	QS complex in one or more leads from V2 to V6	Initial r or q > 40 ms		Initial r wave > 40 ms in V1 or V2	Predominantly negative polarity in 3 out of 4 leads
	QRS complex > 160 ms (LBBB)	AV dissociation	AV dissociation	Notch on the descending limb of a predominantly negative QRS		Notch on the S wave in the V1 lead	Predominantly negative polarity in 2 out of 4 leads
	QRS axis beyond -30º	Morphological criteria		Relação Vi/Vt ≤ 1		Initial R wave in the aVR lead	
	Mono or biphasic QRS in V1 (RBBB)					Interval from the onset of the QRS complex to the apex of the R wave ≥ 50 ms in the DII lead	
	QR or QS in V6 (LBBB)					Absence of RS in the precordial leads	
						AV dissociation	

**Figure 5.1:** Examples of Brugada criteria for diagnosing ventricular tachycardia.

**Figure 5.2:** Steuer criteria for diagnosing ventricular tachycardia.

## 6. Cardiac Chambers hypertrophy

### 6.1. Atrial hypertrophy

#### 6.1.1. Left Atrial Hypertrophy

- P wave duration: ≥ 120 ms (D2 lead), sometimes a P wave with two peaks (right and left atrial components ≥ 40 ms);
- Morris Index: P wave with an increased negative component in V1 lead (negative component of ≥ 1 mm^2^).

#### 6.1.2. Right Atrial Hypertrophy

Isolated right atrial hypertrophy is rare but is frequently associated with right ventricular hypertrophy. ECG findings:

- P waves amplitude: > 0.25 mV or 2.5 mm (D2 lead);
- V1 lead: positive initial portion > 0.15 mV or 1.5 mm;
- Some indirect signs: Peñaloza-Tranchesi (low-voltage QRS complex in V1 with an abruptly QRS amplitude increase in V2) and Sodi-Pallares (QR, Qr, qR, or qRS complexes in V1).

#### 6.1.3. Biatrial hypertrophy

There are both characteristics of right and left atrial hypertrophy.

#### 6.1.4. Left Ventricular Hypertrophy 58 - 68

Although the echocardiogram has a high accuracy for identifying LVH, the ECG has important prognostic significance when abnormal. The currently used criteria include:

##### 6.1.4.1. Romhilt-Estes Criteria ^66^

According to these criteria, LVH is present when 5-point score is achieved. Limitations: LBBB, RBBB, atrial flutter, atrial tachycardia, atrial fibrillation.

- *3 points* : increased QRS amplitude (≥ 20 mm in the frontal plane and/or ≥ 30 mm in the transversal plane); strain pattern (free of digitalis influence); Morris index;
- *2 points* : left QRS axis deviation beyond -30º;
- *1 point* : increased ventricular activation time (VAT) or intrinsicoid deflection > 40 ms; increased QRS duration (> 90 ms) in V5 and V6; and a strain pattern under digitalis influence.

##### 6.1.4.2. Sokolow-Lyon Index ^60^

LVH is considered when the sum of S wave amplitude (V1 lead) + R wave amplitude (V5/V6 lead) is ≥ 35 mm. In young people, this threshold can be 40 mm. This index should not be used in athletes.

##### 6.1.4.3. Cornell Index ^58^

LVH is considered when the sum of R wave amplitude (aVL lead) + S wave amplitude (V3 lead) is ≥ 28 mm in men and ≥ 20 mm in women.

##### 6.1.4.4. Peguero-Lo Presti 67 , 68

LVH is considered when the sum of the deepest S wave in all 12 leads + S wave in V4 is ≥ 28 mm in men and ≥ 23 mm in women.

##### 6.1.4.5. Changes in Ventricular Repolarization

A flat T wave in the left leads (D1, aVL, V5, and V6), or a strain pattern (ST depression ≥ 0.5 mm with a negative and asymmetrical T wave).

## 6.1.5. Right Ventricular Hypertrophy 69 - 72

### 6.1.5.1. QRS Axis

A QRS axis shift located to the right beyond +110° - frontal plane.

### 6.1.5.2. Tall R Wave

A tall R wave in V1 and V2 (R/S ratio ≥ 1), and deep S waves in the opposite leads (V5 and V6).

### 6.1.5.3. qR or qRs Morphology

A qR or qRs morphology in V1 (or V1 and V2) is one of the most specific signs of RVH. It indicates an increased intraventricular pressure (systolic).

### 6.1.5.4. rsR’ Morphology

A triphasic pattern (rsR’), with prominent R wave in the right precordial leads V1 and V2. It indicates an increased intraventricular pressure (diastolic) with an enlarged chamber.

### 6.1.5.5. Ventricular Repolarization

ECG can present a strain pattern (ST-segment depression with a negative T wave) in the right precordial leads (V1, V2, and, sometimes, V3).

### 6.1.5.6. Seattle Criteria for RVH

RVH is considered when the sum of R wave in V1 + S wave in V5–V6 > 10.5 mm (and right axis deviation > 120º).

## 6.1.6. Biventricular Hypertrophy

- a) Frontal plane QRS axis deviated to the right, associated with voltage criteria for LVH;
- b) An ECG typical of RVH, associated with one or more of the following features:
  b.1) deep Q waves in V5 and V6 and in inferior leads;
  b.2) Increased R wave voltage in V5 and V6;
  b.3) Sokolow-Lion criterion for LVH (S in V1-V2 + R in V5-V6);
  b.4) Intrinsicoid deflection in V6 ≥ 40 ms.
- c) Large biphasic QRS complexes, with R-S > 50 mm, in mid-precordial leads (V2 to V4 - Katz-Wachtel phenomenon).

## 6.1.7. Differential Diagnoses for Increased QRS Amplitude ^73^

Increased QRS amplitude is most commonly seen with ventricular hypertrophy. However, QRS may be increased in normal individuals in the following situations:

- Children, adolescents, and young adults;
- Slender individuals;
- Athletes;
- Women who underwent mastectomy surgery;
- Vagotonia.

## 7. Analysis of Intraventricular Blocks (Conduction Delay)

### 7.1. Intraventricular Blocks 74 , 75

Although the concept of “bundle-branch block” is well established in the literature, various degrees of delays in the intraventricular propagation of electrical impulses can occur, leading to changes in the morphology and duration of the QRS complex. These changes in intraventricular conduction may be fixed or intermittent, and also rate-dependent. These blocks may be caused by structural changes in the His-Purkinje conduction system or in the ventricular myocardium (necrosis, fibrosis, calcification, infiltrative diseases, or vascular insufficiency), or functional changes (due to the relative refractory period of part of the conduction system), generating aberrant intraventricular conduction.

#### 7.1.1. Left Bundle-Branch Block (LBBB) 76 , 77

- Wide QRS ≥ 120 ms (as an essential condition); classical manifestations of LBBB, however, width ≥ 130 ms in women and ≥ 140 ms in men);
- Absence of a q wave in D1, aVL, V5, and V6; variants may have a q wave in aVL only.
- Wide R waves, with notches and/or mid-terminal slurring in D1, aVL, V5, and V6;
- Delayed r wave progression from V1 to V3 (sometimes with QS complexes);
- Intrinsicoid deflection in V5 and V6 ≥ 50 ms.
- QRS axis between -30º and +60º;
- ST-segment depression and asymmetrical T wave opposed to the mid-terminal delay.

##### 7.1.1.1. LBBB in Association with LVH 78 - 79

The electrocardiographic diagnosis of LVH in association with LBBB is not trivial due to changes in the QRS complex inherent to the LBBB. Studies show variable results regarding the accuracy of electrocardiographic criteria for LVH:

- Left atrial hypertrophy;
- QRS duration > 150 ms;
- R wave in aVL > 11 mm;
- S waves in V2 > 30 mm and in V3 > 25 mm;
- QRS axis beyond -40º;
- Sokolow-Lyon Index ≥ 35 mm.

##### 7.1.1.2. LBBB in Association with RVH ^80^ (at Least 2 out of 3 Criteria)

- Low voltage in the precordial leads;
- Prominent R wave in aVR;
- R/S ratio < 1 in V5.

## 7.1.2. Right Bundle-Branch Block (RBBB) 81 , 82

- Wide QRS ≥ 120 ms as an essential condition;
- Slurred S waves in D1, aVL, V5, and V6;
- qR waves with slurred R wave in aVR;
- rSR’ or rsR’ with thickened R’ in V1;
- Variable QRS axis, usually shifted to the right in the frontal plane;
- Asymmetrical T wave opposed to the delay of the end of the QRS complex.

### 7.1.2.1. End Conduction Delay

This expression may be used when there is a subtle conduction disturbance in the right bundle-branch. It can be a normal variant and sometimes is also called incomplete right bundle-branch block.

## 7.1.3. Left Fascicular Blocks 83 - 92

A conduction delay that affects one of the left bundle-branch divisions may generate an upward/leftward shift (LAFB) or a downward/rightward shift (LPFB) or an anterior shift (left anteromedial fascicular block) of the QRS axis.

### 7.1.3.1 Left Anterosuperior Fascicular Block (LAFB) 83 - 87

- QRS axis ≥ -45º;
- rS complex in D2, D3 and aVF with an S3 greater than S2; QRS duration < 120 ms;
- S wave amplitude ≥ 15 mm in D3 (or equivalent area);
- qR complex in D1 and aVL with an intrinsicoid deflection time ≥ 50 ms or qRs complex with a minimal “s” wave in D1;
- qR complex in aVL with slurred R wave;
- Slow r wave progression from V1 to V3;
- Presence of S wave from V4 to V6.

### 7.1.3.2. Left Anteromedial Fascicular Block 88 - 90

- qR complex from V1 to V4.
- ncreasing R wave from V1 to V3 (≥ 15 mm) and decreasing QRS complex amplitude from V4 to V6;
- QRS duration < 120 ms;
- No deviation of the frontal plane QRS axis;
- T waves generally negative in the right precordial leads.

All the mentioned criteria are valid in the absence of RVH, septal hypertrophy, or old lateral myocardial infarction.

### 7.1.3.3 Left Posteroinferior Fascicular Block (LPFB) 83 - 85 , 91 , 92

- Frontal plane QRS axis shifted to the right > +90º;
- qR complex in D2, D3 and aVF with R3 > R2 and an intrinsicoid deflection > 50 ms;
- R wave in D3 > 15 mm (or equivalent area);
- Intrinsicoid deflection duration increased in aVF, V5–V6 ≥ 50 ms;
- rS complex duration < 120 ms in D1; slower R wave progression may occur from V1–V3;
- S wave from V2 to V6.

All these criteria are valid in the absence of a slender body type, RVH, and old lateral myocardial infarction.^80 , 91^

## 7.1.4. Right Fascicular Blocks ^82^

### 7.1.4.1. Right Superior Fascicular Block

- rS complex in D2, D3, and aVF with S2 > S3 (differentiating it from the LAFB);
- Rs complex with an S wave > 2 mm in D1, rS complex duration < 120 ms, rS complex in D1 or D1, D2, and D3 (S1, S2, S3);
- Slurred S waves in V1–V2/V5–V6 or, eventually, rSr’ complex in V1 and V2;
- qR complex with slurred R wave in aVR.

### 7.1.4.2. Right Inferior Fascicular Block

- R wave in D2 > R wave in D3;
- rS complex duration < 120 ms in D1;
- Frontal plane QRS axis shifted to the right > +90º;
- Slurred S waves in V1–V2/V5–V6 or, eventually, rSr’ complex in V1 and V2;
- qR complex with slurred R wave in aVR.

Given the difficulty in recognizing right fascicle blocks, the term “intraventricular end conduction delay” may be used.

## 7.1.5. Bundle-Branch and Fascicular Blocks Association ^93^

### 7.1.5.1. LBBB in Association with LAFB

An LBBB with a frontal plane QRS axis shifted to the left, beyond -30º, suggests the presence of LAFB.

### 7.1.5.2. LBBB in Association with LPFB

An LBBB with a frontal plane QRS axis shifted downwards and to the right, beyond +60º, suggests an association with LPFB, RVH, or congenital heart disease.

### 7.1.5.3. RBBB in Association with LAFB

An RBBB with a frontal plane QRS axis shifted to the left, beyond -30º, suggests the presence of LAFB.^94 , 95^

### 7.1.5.4. RBBB in Association with LPFB

An RBBB with a frontal plane QRS axis shifted downwards and to the right, beyond +120º, suggests an association with LPFB.

### 7.1.5.5. RBBB in Association with LAFB and Left Anteromedial Fascicular Block

RBBB in association with LAFB and left anteromedial fascicular block follows the same bundle-branch and fascicular block criteria described above.

### 7.1.5.6. LAFB in Association with Left Anteromedial Fascicular Block

LAFB and left anteromedial fascicular block follows the same fascicular block criteria described above.

### 7.1.5.7. Masquerading Bundle-Branch Block 96 , 97

RBBB pattern in V1 (R or rR’ complex) and an LBBB pattern in the frontal plane leads with LAFB. The S wave in D1 is normally absent or below 1 mm. In the presence of these associations, axis deviations are more prominent.

## 7.1.6. Special Situations Involving Intraventricular Conduction

### 7.1.6.1. Peri-infarction Conduction Block ^98^

QRS complex duration is increased in the presence of an abnormal Q wave due to myocardial infarction (inferior or lateral leads). QRS complex final portion is increased (QR complex).

### 7.1.6.2. Peri-ischemia Block 98 , 99

Peri-ischemia block occurs when there is a transient increase in QRS complex duration with ST-segment elevation (acute phase).

### 7.1.6.3. QRS Complex Fragmentation (fQRS) 99 , 100

Presence of notches in the R or S waves in 2 contiguous leads in the absence of bundle-branch block. With a narrow QRS, notches are more clearly seen in the inferior leads. With bundle-branch block more than 2 notches are needed. This diagnosis should be differentiated from end conduction delays when the notch appears in the S wave in V1 and V2. The more leads with fragmentation are observed, the worse the prognosis.

### 7.1.6.4. Atypical LBBB ^101^

In a patient with previous LBBB with a new infarction, there are deep and wide Q waves, a QS complex pattern in V1–V4, and QR complex in V5–V6, with QRS fragmentation.

### 7.1.6.5 Parietal or Purkinje/Muscle or Focal Intraventricular Block ^102^

This dromotropic disturbance is located between the Purkinje fibers and the muscle. It is seen in severe hypertrophies and cardiomyopathies. May be associated with LAFB or LVH, and the duration of the QRS complex is ≥ 120 ms, without LBBB morphology or LBBB with LAFB morphology.

## 8. Analysis of the ECG In Coronary Heart Disease

It is important to highlight that a normal ECG does not exclude the presence of a coronary event. Specific clinical guidelines for acute coronary syndromes should be followed.^103 , 104^

### 8.1. Diagnostic Criteria for Myocardial Ischemia ^105^

#### 8.1.1. Presence of Ischemia

- *Hyperacute phase* – peaked and symmetrical T wave as the initial presentation;
- *Subendocardial ischemia* – positive, symmetrical, and peaked T wave;
- *Subepicardial ischemia* – negative, symmetrical, and peaked T wave. This alteration is currently attributed to a pattern of reperfusion or edema instead of real ischemia of the subepicardial region.^106^

#### 8.1.2. Circumferential or Global Ischemia 107 , 108

A peculiar situation during an angina episode, with ST-segment depression in 6 or more leads, particularly in V4–V6, along with negative T waves associated with an ST-segment elevation > 0.5 mm in aVR.

#### 8.1.3. Secondary Changes

Secondary changes in the T wave are those not fitting within the definition of ischemic waves, especially due to asymmetry and the presence of other diagnostic characteristics such as chamber hypertrophy or intraventricular blocks.

## 8.2. Subendocardial and Subepicardial Injury: Diagnostic Criteria

- *Subepicardial injury* – J-point and ST-segment elevation, with upper concavity or convexity (more specific) of the segment in 2 contiguous leads, of at least 1 mm in the frontal plane and left precordial leads. In precordial leads (V1 to V3), ST-segment elevation should be ≥ 1.5 mm in women, ≥ 2 mm in men aged 40 years or older, and ≥ 2.5 mm in men aged less than 40 years;^109^
- *Subendocardial injury* ^109^– J-point and ST-segment depression, horizontal or downsloping ≥ 0.5 mm, in 2 contiguous leads, at 60 ms after the J-point.

Note: to diagnose injury one should consider the concomitant presence of changes in the T wave and ST-segment, recognized in at least 2 concordant leads.

## 8.3. Definition of Myocardial Fibrosis

An area with fibrosis (old myocardial infarction) is considered when ventricular activation does not occur as expected and does not suggest intraventricular conduction disturbance. Myocardial fibrosis (old myocardial infarction) is characterized by pathological Q waves in 2 contiguous leads, with duration ≥ 40 ms, associated or not with amplitude > 25% of the QRS amplitude, or a reduced R wave in an area where it is expected and should be present.

## 8.4. Topographic Analysis of Ischemia, Injury, and Necrosis

### 8.4.1. ECG Topographic Analysis of Ischemic Manifestations (Meyers)

- Anteroseptal wall – V1, V2, and V3 leads;
- Anterior wall – V1, V2, V3, and V4 leads;
- Anterolateral wall – V4 to V5, V6, D1, and aVL leads;
- Extensive anterior wall – V1 to V6, D1, and aVL leads;
- Lateral wall – D1 and aVL leads and/or V5 and V6 leads;
- Inferior wall – D2, D3, and aVF leads.

Note: The term “posterior wall” should no longer be used due to current evidence indicating that the recording of leads V7–V9 refers to the lateral wall.^110^

### 8.4.2. ECG Topographic Analysis of Ischemic Manifestations in Association with Magnetic Resonance Imaging ^111^

- Septal wall – Q wave in V1 and V2 leads;
- Anteroapical wall – Q wave in V1, V2 to V3–V6 leads;
- Anteromedial wall – Q wave (qS complex or r wave) in D1, aVL, occasionally in V2 and V3 leads;
- Lateral wall – Q wave (qR1 complex or r wave) in D1, aVL, V5–V6 and/or RS complex in V1 lead;
- Inferior wall – Q wave in D2, D3, and aVF leads.

The sites mentioned above present the best anatomic correlations in acute coronary syndromes with ST-segment elevation and in necrosis (when present). Topographic sites may vary in the case of cardiomegaly or major structural alterations.

### 8.4.3. Electrocardiographic Correlation with the Culprit Artery ( Table 8.1 ) ^112^

**Table 8.1:** – Correlation between electrocardiographic leads and culprit artery

		ST-segment elevation			ST-segment depression
**Left coronary branch**		aVR			V2-V6; I,L
**Anterior descending coronary artery**	Before the first septal branch	V1 - V4		I, L	II, III, F
**Anterior descending coronary artery**	Between the septal and diagonal branches	V1 - V6		I, L	
**Long left anterior descending coronary artery (post-crux cordis)**	After the septal and diagonal branches	V2 - V6		I, L	V2-V6; I,L
**Proximal right coronary artery**		V4 - V6	II < III, F		I, L, V1 - V3
**Mid-distal right coronary artery**			II < III, F	I, L	I, L, V1 - V3
**Distal right coronary artery**			II < III, F		I, L
**Right coronary artery (right ventricle)**		V1, V3R, V4R	II < III, F		
**Circumflex coronary artery**		V4 - V6	II > III, F	I, L	V1 - V3
**Circumflex coronary artery (right ventricle)**		V1, V3R, V4R; V4 - V6	II > III, F	I, L	

In Figure 8.1 we find the correlation between the culprit artery and the wall/ventricular segment involved.

**Figure 8.1:** Correlation between culprit artery and wall/ventricular segment (adapted from Selvester RH et al.) 112

## 8.5. Particular Areas of Infarction

### 8.5.1. Right Ventricle Myocardial Infarction

ST-segment elevation in the right precordial leads (V1, V3R, V4R, V5R, and V6R), particularly with ST-segment elevation > 1 mm in V4R. ST-segment elevation in right ventricle infarctions appears for a short period of time due to the low oxygen consumption of the right ventricular muscle. In general, this infarction is associated with low inferior wall and/or lateral wall infarctions of the left ventricle.^113^

### 8.5.2. Atrial Infarction

Atrial infarction can be recognized by the presence of PR segment elevation > 0.5 mm. It can be associated with atrial arrhythmias.^114^

## 8.6. Differential Diagnoses ^115^

### 8.6.1. Subepicardial Ischemia

It should be differentiated from secondary changes in ventricular repolarization in LVH or bundle-branch blocks (asymmetrical T wave).

### 8.6.2. Acute Myocardial Infarction (AMI) with ST-segment Elevation

It should be differentiated from:

- Early repolarization;
- Pericarditis and myocarditis;
- Former AMI with dyskinetic area and persistent ST-segment elevation (left ventricular aneurysm);
- Acute pancreatitis;
- Hyperkalemia;
- Catecholaminergic syndromes;
- Brugada syndrome.

## 8.7. Association of Myocardial Infarction with Bundle-Branch Blocks

### 8.7.1. Myocardial Infarction in the Presence of RBBB

The electrocardiographic diagnosis of myocardial infarction is not hindered by the presence of an RBBB.

### 8.7.2. Myocardial Infarction in the Presence of LBBB

The presence of an LBBB hinders the recognition of an associated myocardial infarction. In LBBB, the conduction delay begins with the disappearance of the first vector and mid to terminal ventricular activation impairment. In septal infarctions, a larger and wider R wave is seen (as opposed to the usually small or absent r wave in LBBB) in V1 and/or V2, associated with a q wave in V5 and V6. In lateral infarctions, we notice slurred or notched S waves in the ascending phase. Inferior infarctions result in slurred or notched S waves in D2, D3, and aVF.^116^

ST-segment deviations may allow the identification of recent myocardial infarction, according to criteria defined by Sgarbossa et al. Five points or more indicate a high accuracy in the identification of myocardial infarction with ST-segment elevation.^117^

- 5 points: ST-segment elevation ≥ 1mm concordant with the QRS/T;
- 3 points: ST-segment depression ≥ 1 mm in V1, V2, and V3;
- 2 points: ST-segment elevation ≥ 5 mm discordant with the QRS/T.

## 9. Analysis of Ventricular Repolarization

### 9.1. Ventricular Repolarization

The analysis of ventricular repolarization is extremely complex, as it represents the interaction of various systems that can be expressed through segments and electrical waves. The repolarization phenomenon received greater attention after the recognition of its contribution to the risk stratification of major arrhythmic events and sudden cardiac death.

#### 9.1.1. Normal Ventricular Repolarization

Ventricular repolarization comprehends the period between the end of the QRS and the end of the T wave (or U wave, when present). In this context, the following elements should be analyzed:

##### 9.1.1.1. J-point

J-point is located at the end of the QRS complex. Its position is used to identify ST-segment deviations.

##### 9.1.1.2. ST-Segment

ST-segment is located between the J-point and the T wave. It should be at the same level as the PR segment. Variation up to 0.5 mm (up or down) is considered within the normal range.

##### 9.1.1.3. T Wave

A normal T wave is asymmetrical, with a slower onset and a faster ending. It is also positive in almost all leads, and its amplitude corresponds to 10%-30% of the QRS amplitude. It is always negative in aVR and may be negative only in V1 and/or D3.

##### 9.1.1.4. U Wave

U wave is the last and smallest deflection in the ECG. When present, it occurs soon after the T wave and before the P wave of the next cycle. Its polarity is the same as that of the preceding T wave, and in most cases its amplitude corresponds to 5%-25% of the preceding T wave. In general, it is visible only in low heart rates and its genesis is attributed to the following:

- Late repolarization of the Purkinje fibers;
- Slow repolarization of papillary muscles;
- Late residual potentials in the septum;
- Electromechanical coupling;
- M cell activity;
- Delayed afterdepolarization (triggered activity).

##### 9.1.1.5. QT interval and Corrected QT Interval (QTc)

- *QT –* Measurement from the beginning of the QRS to the end of the T wave. It represents the total duration of ventricular electrical activity;
- *QTc –* QT is modified by the heart rate. In general, its correction (QTc) uses the Bazett formula:

$$QTc=\frac{QT^{*}}{\sqrt{RR}}$$

^*^QT measured in milliseconds and RR distance measured in seconds.

The Bazett formula,^118^although widely used for calculating QTc, presents limitations for HRs below 60 bpm or above 90 bpm. In these cases, linear formulas such as those by Framingham^119^and Hodges^120^should be used.

QT and QTc values do not need to be reported, but they should always be checked for normality. QTc values vary with gender and are accepted as normal when up to 450 ms for men and 470 ms for women. For children, the upper limit of normality is 460 ms,^121^and QT is considered short when below 340 ms.^122^

The measurement of the QT interval in bundle-branch blocks is controversial, and a simplified correction was recently proposed by Bogossian: QTm = QT_LBBB_- 0.5 QRS_LBBB_.^123^

## 9.1.2. Variants of Ventricular Repolarization

### 9.1.2.1. Early Repolarization Pattern

Historically, the ECG with “early repolarization” has always been considered normal. Some publications have associated the slurring or notching of the final portion of the QRS (also named early repolarization) with a higher death rate; thus caused scientific turmoil regarding the benignity of this condition. Early repolarization is characterized by the mandatory presence of a notch or slurring of the final portion of the QRS complex; J-point elevation may or may not be found.^124^

J wave with a straightened aspect of the ST in the inferior leads (or associated with lateral leads) may be an electrical marker of ventricular tachyarrhythmias risk.^125 - 129^

In the last decades, great advances have linked the ventricular repolarization features to risk stratification of major arrhythmic events and sudden cardiac death. These include the dispersion of ventricular repolarization as a marker of the non-uniform recovery of myocardial excitability and the recognition of cyclic (macrovolt or microvolt) T-wave alternans. It is important to consider significant changes in polarity, duration, and morphology of the electric phenomena described above as ventricular repolarization alterations.

## 10. Channelopathies and Other Genetic Alterations

### 10.1. Genetics and the ECG

In recent years, the improvement of genetic mapping techniques allowed a deeper understanding and differentiation of potentially fatal clinical conditions with characteristic electrocardiographic patterns. Within this group, we highlight conditions that affect structurally normal hearts, such as channelopathies and others with myocardium involvement such as hypertrophic cardiomyopathy and arrhythmogenic right ventricular cardiomyopathy.

#### 10.1.1. Channelopathies

Cardiac channelopathies are a result of genetic mutations or malfunctioning of ion channels, resulting in important modifications of the action potential. Some specific electrocardiographic findings associated with syncope (at rest or triggered by exercise), as well as ventricular arrhythmias in structurally normal hearts, should raise the hypothesis of channelopathies.

##### 10.1.1.1. Congenital Long QT Syndrome 129 , 130

Congenital long QT syndrome was the first described and most studied channelopathy. It allowed the understanding of the relationship between molecular biology and genetics, and the association with clinical manifestations, risk stratification, and treatment. Congenital long QT syndrome represents the main cause of a negative autopsy in cases of sudden death among young people.

Its main characteristic is the prolongation of the QTc on the ECG, with values > 460 ms. Clinically the presence of syncope or cardiac and respiratory arrest triggered by emotional and physical stress should raise the hypothesis of long QT syndrome. People with this condition are at a high risk of polymorphic VT, syncope, and sudden death (when polymorphic VT degenerates into VF). *Torsades des pointes* (TdP) is a polymorphic VT in a person with long QT. Although 16 genes have been linked to mutations associated with long QT syndrome (LQT), 3 of them correspond to 75% of all diagnosed cases: KCNQ1 (LQT1), KCNH2 (LQT2), and SCN5A (LQT3). Some triggers are gene-specific: exercise is more strongly associated with LQT1, emotional stress is associated with LQT2, and bradycardia is associated with LQT3. Characteristics on the ECG include:

- LQT1: T wave with a wide base and delayed beginning;
- LQT2: T wave with low amplitude, usually with notch;
- LQT3: delayed T wave, with a huge ST-segment.

##### 10.1.1.2. Short QT Syndrome 131 - 133

Described in 2000, short QT syndrome is characterized by a short QT interval (< 340 ms) associated with AFib and sudden cardiac death. There is increased activity of potassium channels (phase 3 of the action potential) resulting in shortening of the QT interval. Genes related with this syndrome are KCNH2, KCNQ1, and KCNJ2. When the ECG highlights short QTc (< 370 ms) and a distance < 120 ms between the J point and the peak of the T wave, a diagnosis of short QT is suspected.

##### 10.1.1.3. Brugada Syndrome 134 - 137

Brugada syndrome is a channelopathy caused by sodium channels defect. In most cases it happens in the right ventricular epicardium, affecting men more than women (8:1). Some individuals present syncope and or/cardiac arrest due to VF, besides family history of sudden death. In many cases, these events happen during rest and sleep, and may also be triggered by hyperthermia and some medications, culminating in sudden death.

Brugada syndrome has a dominant autosomal inheritance and is responsible for 20% of all sudden deaths with a normal heart at autopsy. It is a genetically heterogeneous condition, involving at least 13 genes. More than 200 mutations have been described, most of them occur in genes that affect the sodium channels (SCN5A) and it can be identified in only 20% to 25% of all cases.

A J-point elevation ≥ 2 mm in V1 and V2 leads, followed by a slow ST-segment depression with upward convexity and a T-wave inversion, characterizes the type 1 pattern. The diagnosis of Brugada syndrome is made by the electrocardiographic finding of the type 1 pattern in association with symptoms.

Type 2 pattern is characterized by a J-point elevation in V1 and V2 < 2 mm and a saddle-shaped ST-segment. This pattern is highly suspect but does not confirm the diagnosis. Its transient pattern hinders diagnosis, and in doubtful cases, ECG should be recorded with upper precordial leads. The electrodes are positioned in the second and third right and left intercostal spaces, which allow better assessment of the right ventricle outflow tract. This electrode position increases ECG sensitivity for type 1 pattern diagnoses.^138^

Brugada phenotype is characterized by the presence of Brugada type 1 ECG pattern in a person with no history of aborted sudden death or syncope, or sudden death in first-degree relatives.

Brugada phenocopy is not the same as Brugada phenotype, and is characterized by an electrocardiographic pattern that is presumably identical to the syndrome, but caused by many other conditions. It presents a transmural gradient (epicardium to endocardium) resulting from a sharp notch on the epicardial action potential, mediated by I_to_channels and by the loss of the action potential dome. Among the described situations, there are metabolic alterations, mechanical compression by extra-cardiac structures, ischemia, myocardial/pericardial disease, and some medications.^139^The ECG with Brugada pattern disappears after the resolution of these conditions.

##### 10.1.1.4. Catecholaminergic Tachycardia 140 , 141

Catecholaminergic tachycardia affects individuals during childhood and adolescence. Some of them report syncopal episodes and a family history of sudden death. Hereditary or sporadic mutations in ryanodine channels, responsible for regulation of the intracellular calcium, are responsible for 50% to 60% of all cases of catecholaminergic polymorphic VT. The resting ECG may be normal or with sinus bradycardia and/or U waves. Bidirectional ventricular arrhythmia triggered by exercise testing or isoproterenol infusion is typical.

Other frequent findings include PVC, which are usually isolated, intermittent, bigeminal, and paired, increasing in density with exercise.

## 10.1.2. Genetic Diseases with Primary Cardiac Involvement

### 10.1.2.1. Arrhythmogenic Right Ventricular Cardiomyopathy (Dysplasia) 142 - 144

Arrhythmogenic right ventricular cardiomyopathy is a genetic disease with primary involvement of the right ventricle. There is a replacement of myocytes with fibrofatty tissue and it is associated with arrhythmias, heart failure, and sudden death. ECG is characterized by a low voltage and longer duration QRS complex in V1/V2 (epsilon wave, present in 30% of the cases), associated with negative, rounded, and asymmetrical T waves from V1 to V4. It is associated with PVC originating from the right ventricle (LBBB morphology) and may have a superior or inferior orientation. The finding of negative T waves up to V6 suggests left ventricle involvement.

### 10.1.2.2. Hypertrophic Cardiomyopathy 145 , 146

Hypertrophic cardiomyopathy is a primary heart disease with genetic basis. It has mostly autosomal dominant inheritance, affects 1:500 live births. There are several described mutations and with severe segmental or diffuse ventricular hypertrophy. In almost 75% the ECG is altered, and in the pediatric population it has a good sensitivity.^147^It is characterized by rapid and deep Q waves in the inferior and/or precordial (lateral) leads, generally associated with classical ECG signs of LVH and accompanied by characteristic ST-T changes (deep and negative T waves).

## 10.1.3. Genetic Diseases with Secondary Cardiac Involvement

### 10.1.3.1. Muscular Dystrophy ^148^

Muscular dystrophy is a group of diseases that predominantly affect voluntary muscles. In some of them, there is respiratory and heart muscle involvement. The most common ECG findings are tall R wave in V1 and V2 (R/S ratio > 1), deep Q wave in V6, D1, and aVL, right bundle-branch conduction delay, QS complexes in D1, aVL, D2, and D3, and abnormal ventricular repolarization.

## 11. Electrocardiographic Pattern in Specific Clinical Situations

### 11.1. Clinical Conditions that Can Modify the ECG

Besides heart diseases, some peculiar ECG pattern can be recognized due to systemic diseases, metabolic disorders, and use of medications. In some of them, such as long QT, WPW, and Brugada syndrome, the ECG is the most sensitive and specific diagnostic test.^149^On the other hand, its sensitivity decreases in conditions such as myocardial infarction, pericarditis, and digitalis toxicity, although it is still one of the main diagnostic methods in clinical practice. Myocardial infarction and WPW syndrome, due to their prevalence and importance, are analyzed in separate chapters of this guideline. Other situations were grouped in this section.

We will now analyze the highly specific diagnostic features for the following conditions, in alphabetical order. However, we recommend that the terms “ECG suggestive of” or “ECG consistent with” should be used in the final ECG reports.

#### 11.1.1. Digitalis Action

The use of digitalis can be recognized by ST-T depression with upper concavity (reverse tick or Salvador Dali sagging) and shortened QTc interval. Several arrhythmias may occur in case of digitalis toxicity, especially PVC. Bidirectional ventricular tachycardia and atrial tachycardia (variable AV conduction) is highly suggestive of digitalis toxicity, as well as bradiarrhythmias (first-degree AV block and type I second-degree AV block).

#### 11.1.2. Drug-induced ST-T Changes

Drugs that interfere with the ST-T (increasing QTc interval) can be found on the following electronic address: http://www.azcert.org/medical-pros/drug-lists/drug-lists.cfm .^150^

#### 11.1.3. Electrical Alternans

Electrical alternans is characterized by alternately higher and lower QRS amplitudes. It is cyclic and not related with the respiratory cycle, in successive QRS complexes.

#### 11.1.4. T-wave Alternans (TWA)

The clinical applicability of T-wave alternans has been increasingly investigated. TWA is characterized by beat by beat modification of amplitude, shape, and orientation of the T wave (J-point and/or ST-segment). It may be intermittent or permanent. On the 12-lead ECG, these variations may be macroscopic (macrovolt alternans) or so small that computerized algorithms are required for its analysis (microvolt alternans).

#### 11.1.5. Acute Injury of the Central Nervous System

Hemorrhagic injury of the central nervous system (CNS) can produce giant negative T waves (more rarely positive) simulating subepicardial ischemia that is called cerebral T wave. Increased QTc interval can also be observed. All ECG alterations are reversible after CNS treatment.

#### 11.1.6. Interatrial Communication

An individual with an interatrial communication can present a right end conduction delay and, sometimes, RVH. If an upward and leftward deviation of the QRS axis is also present it is related with the *ostium primum* defect. Supraventricular arrhythmias, such as atrial fibrillation/flutter, is not uncommon.

#### 1.1.7. COVID-19

Cardiac impairment due to COVID-19 may reach 44% of severe cases; electrocardiographic alterations were observed in up to 93% of hospitalized patients with critical illness. The reasons for myocardial alterations with changes in the ECG include cytokine storm, hypoxic injury, electrolyte imbalance, plaque rupture, coronary artery spasm, microthrombi, or direct endothelial or myocardial injury. The ECG may present supraventricular tachycardia (sinus tachycardia, atrial fibrillation, atrial flutter, AVNRT), malignant ventricular arrhythmia (monomorphic and polymorphic VT and VF), bradycardia, and AV blocks (second- and third-degree), increased QT interval, RBBB and LBBB, QRS axis rightward deviation, ST-segment elevation or depression, T-wave inversion, pathological Q waves, and signs of pulmonary thromboembolism (sinus tachycardia/atrial fibrillation, RVH, RBBB, T-wave inversion from V1 to V3, S1Q3T3 pattern). Moreover, COVID-19 can uncover the Brugada pattern in individuals with this disease.^151^

#### 11.1.8. Pericardial Effusion

In pericardial effusion, ECG can present dielectric effect (see Item 11.14), sinus tachycardia, and electrical alternans.

#### 11.1.9. Dextrocardia

Individuals with dextrocardia present negative P, QRS and T waves in D1 and V6. Positive QRS complexes in V1 and negative QRS in V6 are also observed (main differential diagnosis with reversal of the upper limb electrodes).

#### 11.1.10. Dextroposition

Individuals with dextroposition may manifest a negative or minus-plus P wave in D1, a deep Q wave in D1 and aVL, and qRS complexes in the right precordial leads.

#### 11.1.11. Electrolyte Imbalance

##### 11.1.11.1. Hyperkalemia

ECG changes depend on serum potassium concentrations, and they occur sequentially: symmetrical T wave with increased amplitude and a narrow base, decreased QTc interval, intraventricular conduction delay (wide QRS), and decreased P wave amplitude until its disappearance, with sinoventricular rhythm.

##### 11.1.11.2. Hypokalemia

ECG can present increased U wave amplitude; ST-segment and T-wave depression; increased QTU interval. The QT interval should preferably be measured in the aVL lead because the U wave tends to be more isoelectric.

##### 11.1.11.3. Hypocalcemia

In patients with hypocalcemia, ECG can show flattening and increased duration of the ST-segment with an increase in the QTc interval.

##### 11.1.11.4. Hypercalcemia

In patients with hypercalcemia, ECG can show shortening and eventual disappearance of the ST-segment, with a consequent shortening of the QT interval.

## 11.1.12. Chronic Obstructive Pulmonary Disease (COPD)

COPD frequently presents ECG with dielectric effect (see Item 11.1.14); rightward QRS axis shift; rS complex from V1 to V6. Right chambers hypertrophy is associated to heart disease and a rightward shift of the P axis, near +90º (P *pulmonale* ).

## 11.1.13. Antiarrhythmic Drugs

Antiarrhythmic drugs can be related with proarrhythmia. In the following sections, we will present the drugs that can change the ECG.

### 11.1.13.1. Amiodarone

Amiodarone is a Class III antiarrhythmic drug; it may cause PR interval prolongation, sinus bradycardia, increased QTc (with no increase in the dispersion of repolarization), and T wave alterations. All of them are more clearly seen in the precordial leads with bifid or flattened T waves. These changes can also be observed in the frontal plane leads (it differentiates them from bifid T waves in children, which are limited to the precordial leads).

### 11.1.13.2. Propafenone

Propafenone is a Class IC antiarrhythmic drug that may cause AV blocks and complex ventricular arrhythmias (paired PVC, nonsustained VT, and polymorphic VT), especially in patients with coronary artery disease.

### 11.1.13.3. Sotalol

Sotalol is a Class III antiarrhythmic drug and may cause sinus bradycardia, AV blocks, and increased QTc interval (with polymorphic VT).

## 11.1.14. Dielectric Effect

Dielectric effect can present an ECG with low QRS voltage (< 0.5 mV in the frontal plane and < 1.0 mV in the precordial leads). It may result from a large pericardial effusion, pleural effusion, emphysema, COPD, morbid obesity, or anasarca. Hypothyroidism and infiltrative heart diseases can also present a low voltage pattern.

## 11.1.15. Pulmonary Embolism

Patients with pulmonary embolism can present sinus tachycardia, right end conduction delay, acute QRS axis rightward deviation, ST-segment depression (V1 to V3), and negative T waves from V1 to V3. The classical S1Q3T3 pattern may occur.

## 11.1.16. Ashman or Gounaux-Ashman Phenomenon ^17^

Ashman phenomenon is an aberrant conduction of a beat from supraventricular origin. It can happen after a long-short cycle. Due to its characteristic long refractory period (compared to the left bundle-branch) the aberrancy occurs more frequently at the right bundle-branch. Irregular rhythms such as atrial fibrillation, atrial flutter and atrial tachycardia favor this phenomenon.^152 , 153^Concealed transeptal retrograde conduction is responsible for the following consecutive beats with aberrancy.

## 11.1.17. Hypothermia

Patients with hypothermia can present bradycardia, prolonged QT interval and J wave or Osborn wave (notch with large amplitude and short duration at the final portion of a QRS.

## 11.1.18. Hypothyroidism

In severe cases (myxedema), it may present bradycardia, low voltage, and diffuse alterations of ventricular repolarization.

## 11.1.19. Chronic Renal Failure

Patients with chronic renal failure can present ECG abnormalities related to hyperkalemia and hypocalcemia (discussed elsewhere), and these findings can be associated with decreased renal function.

## 11.1.20. Pericarditis ^154^

The following electrocardiographic alterations may be seen in the acute phase of the inflammatory process, usually in this order:

- *PR-segment depression* in D1, D2, aVF, and from V2 to V6. PR segment elevation in aVR; it may also occur in V1;
- *ST segment* – diffuse elevation with upper concavity, except for V1 and aVR. There are no associated Q waves;
- *T wave* – slightly increased and symmetrical in the initial phase. Characteristically, it is not inverted in the presence of ST elevations. It may be inverted in the chronic phase of the disease, after normalization of the ST segment, but rarely with enough depth to resemble the ischemic T-wave pattern.

## 11.1.21. Chemotherapy Drugs 155 - 158

The development of new chemotherapy drugs has increased patient’s survival. ECG abnormalities may occur in patients with chemotherapy-induced myocardial injury. These alterations are characterized by a complex pathogenesis and may depend both on the direct toxicity of chemotherapy drugs (with electrophysiological substrate) and on direct injure to the myocardium, endocardium, and pericardium (due to ischemia, inflammation, or radiation); this is why alterations accompanying cardiac dysfunction are unspecific and can precede symptoms, even before ECG alterations appear. Electrocardiographic findings are better identified through with serial ECGs. These include sinus tachycardia, T-wave flattening or inversion, increased QT interval, and low QRS voltage.

Some arrhythmias, such as TdP, and VT/VF may occur during treatment. The most well-known drugs are anthracyclines; however, alkylating agents (cyclophosphamide), antimetabolites (5-fluorouracil), antimicrotubule agents (paclitaxel), immunomodulating drugs (thalidomide), and targeted therapy can cause cardiac damage.

## 12. The ECG in Athletes

### 12.1. The Importance of Understanding the Athlete’s ECG 159 - 164

Many physiological adaptations occur during sport training and some of them can lead to specific electrocardiographic findings, even without anatomical/structural changes. That’s why the interpretation of athletes’ ECGs becomes a challenging task. With the inclusion of the resting ECG in pre-participation evaluations, we should be aware of specific recommendations for this population. At the present time, athlete’s ECG findings can be divided into 3 categories:

#### 12.1.1. Normal ECG Findings (Group 1)

- Increased QRS voltage for LVH or RVH;
- Right bundle-branch conduction delay;
- Early repolarization/ST-segment elevation;
- ST-segment elevation followed by T-wave inversion (V1 to V4) in black athletes;
- T-wave inversion (V1 to V3) in athletes aged less than 16 years;
- Sinus bradycardia/sinus arrhythmia;
- Ectopic atrial rhythm or junctional rhythm;
- First-degree AV block;
- Type I second-degree AV block.

#### 12.1.2. Abnormal ECG Findings (Group 2)

- T wave inversion in other situations;
- ST-segment depression;
- Pathological Q waves;
- LBBB;
- QRS duration ≥ 160 ms;
- Epsilon wave;
- Ventricular pre-excitation;
- Prolonged QT interval;
- Brugada type 1 pattern;
- Severe sinus bradycardia (< 30 bpm);
- Pr interval ≥ 400 ms;
- Type II second-degree AV block;
- Third-degree AV block;
- Two or more PVC;
- Atrial tachyarrhythmias;
- Ventricular arrhythmias.

#### 12.1.3. Borderline ECG Findings (Group 3)

- Upward and leftward QRS axis deviation;
- Left atrial hypertrophy;
- Upward and rightward QRS axis deviation;
- Right atrial hypertrophy;
- RBBB.

Athletes with a normal ECG do not need further investigation if they are asymptomatic and have no family history of hereditary heart diseases and/or sudden death. On the other hand, athletes with findings of group 2 should undergo investigation of pathological cardiovascular conditions associated with sudden death. All findings mentioned in this group (2) could be manifestations of structural alterations. Finally, athletes with a borderline ECG (and asymptomatic and no family history of hereditary heart diseases and/or sudden death) are exempted from further investigation if they present only one of the findings in group 3. If these athletes present 2 or more findings from group 3, pathological cardiovascular conditions associated with sudden death in athletes should be investigated.

## 13. The ECG in Children

### 13.1. Introduction

Although the general principles for the interpretation of children’s and adults’ ECGs are similar, the analysis of pediatric ECGs constitutes a challenge for clinical practice. This is mainly due to electrocardiographic patterns that are specific in children ( Table 13.1 ). Such patterns are related with age and anatomical/ physiological changes intrinsic to human development.^165^

**Table 13.1:** Normal electrocardiographic findings in children

Shorter PR interval and narrower QRS complexes
A right QRS axis deviation in the first year of life
Prominent Q waves in the inferior and lateral leads
The analysis of ventricular repolarization is more important than QRS amplitude in the diagnosis of ventricular hypertrophy
Early repolarization
Negative T wave from V1 to V4 until 12 years of age
Bifid or notched T wave in the right precordial leads
Prominent U wave
Common and physiological rhythm findings: Prominent U wave inus arrhythmia, ectopic atrial rhythm First-degree and type I second-degree AV blocks Sporadic atrial and ventricular extrasystoles

The ECG of newborns reflects the hemodynamic effects of the fetal circulation on the right ventricle, as well as anatomical and physiological changes resulting from the transition to neonatal circulation. Up to 32 weeks of pregnancy, the left ventricle is larger than the right ventricle. From this phase and until the end of pregnancy, the right ventricle prevails due to the progressive increase in pulmonary vascular resistance.^166^During birth, lung aeration leads to a sharp drop in pulmonary arterial pressure, while the removal of the placenta and closure of the arterial duct increase systemic vascular resistance.^167^In general, at the end of the first month of life, the left ventricle size equals the right ventricle, and then anatomically prevails over it.^166 , 167^Most of these adaptive changes happen after birth and before the first year of life. Maturation of the autonomic nervous system, physical growth, and changes in the position of the heart occur progressively up to adult age.^168^As a result, the normal ECG changes rapidly during the first weeks of life, and the child starts to gradually present electrocardiographic patterns that are similar to those of an adult only by the age of 2 to 3 years.^167^

### 13.2. Technical Aspects

The ECG of children should include the classical 12 leads, which may be complemented by V3R and V4R leads in case of suspected right chamber hypertrophy.^169^Artifacts are common and usually caused by inadequate electrode placement, chest wall deformities, and movements (voluntary or not) that are intrinsic to each age group.^170^

Reference tables and graphs of age-related variations of electrocardiographic parameters are frequently consulted (necessary task).^170^Most of these values, particularly considering the first year of life, derive from Canadian data by Davignon et al.^171^Despite the publication of more recent studies^172^this continue to be the main reference in clinical practice ( Table 13.2 ). It is still debatable whether these data may be extrapolated to the general public. To date, 2 studies have been published based on the Brazilian population. One of them, including almost 100 term newborns with a normal ECG in the first week of life, showed electrocardiographic parameters that were different from those published by Davignon.^173^The second study, with a population over 1 year of age, included more than a million children.^174^Another controversial situation is the computerized and automatic ECG reports. It has questionable accuracy in pediatrics and its routine use is not yet recommended.^170^

### 13.3. Electrocardiographic Parameters and their Variations

The pediatric ECG should be systematically evaluated according to the patient’s age group ( Table 13.2 ), and its analysis should consider the same criteria as that in adults: rhythm, heart rate, P wave (axis, amplitude, and duration), AV conduction, QRS complex (axis, amplitude, duration, and morphology), ST-segment, T wave, and U wave. Measurement of the QT interval and QTc calculation should be routinely performed.^175^

**Table 13.2:** – Normal electrocardiographic parameters by age

	0–1 day	1–3 days	3–7 days	7–30 days	1–3 months	3–6 months	6–12 months	1–3 years	3–5 years	5–8 years	8–12 years	12–16 years
HR	94 - 155	91 - 158	90 - 166	106 - 182	120 - 179	105 - 185	108 - 169	89 - 152	73 - 137	65 - 133	62 - 160	60 - 120
P (mV)	0.01 - 0.28	0.03 - 0.28	0.07 - 0.29	0.07 - 0.30	0.07 - 0.26	0.04 - 0.27	0.06 - 0.25	0.07 - 0.25	0.03 - 0.25	0.04 - 0.25	0.03 - 0.25	0.03 - 0.25
PR D2 (seconds)	0.08 - 0.20	0.08 - 0.14	0.07 - 0.15	0.07 - 0.14	0.07 - 0.13	0.07 - 0.15	0.07 - 0.16	0.08 - 0.15	0.08 - 0.16	0.09 - 0.16	0.09 - 0.17	0.09 - 0.18
QRS (seconds)	0.02 - 0.10	0.02 - 0.07	0.02 - 0.07	0.02 - 0.08	0.02 - 0.08	0.02 - 0.08	0.03 - 0.08	0.03 - 0.08	0.03 - 0.07	0.03 - 0.08	0.04 - 0.09	0.04 - 0.09
QRS axis	59 - 189	64 - 197	76 - 191	70 - 160	30 - 115	7 - 105	6 - 98	7 - 102	6 - 104	10 - 139	6 - 116	9 - 128

HR: heart rate; mV: millivolts.

#### 13.3.1. Heart Rate and Sinus Rhythm

Contractile mass and ventricular compliance are relatively lower in children, particularly during the first year of life. As a result, their cardiac output depends basically on the HR, which is much higher in children than in adults. A healthy newborn can present HR between 150–230 bpm. Normal HR increases from the date of birth until the first and second months of life; on the sixth month, it returns to values similar to those of the first day. From this point on, HR progressively decreases and, around 12 years-old, it reaches values considered normal for adults.^168^

##### 13.3.1.1. Possible Alterations

**13.3.1.1.1. Sinus Arrhythmia**

Very frequent in children, it is generally phasic and associated with breathing.^165^It is less noticeable in higher HR and in newborns, especially in the first week of life.

**13.3.1.1.2. Sinus Tachycardia**

Sinus tachycardia is considered when sinus rhythm presents HR above the 98th percentile for the age (in general lower than 220 bpm).^170 , 171^Sinus tachycardia may have various causes, of which the most frequent are: physical activity, fever (10-bpm increase in HR for each 1ºC increase in body temperature), anemia, and dehydration.^170^

**13.3.1.1.3. Sinus Bradycardia**

Sinus bradycardia is considered when sinus rhythm presents HR below the second percentile for the age^170 , 171^( Table 13.2 ). It may have various etiologies, such as infections, respiratory failure, hypothermia, hypothyroidism, and increased intracranial pressure. In newborns, the occurrence of transient sinus bradycardia can be associated with the transplacental passage of anti-Ro/SSA antibodies, especially in mothers with systemic lupus erythematosus or other connective tissue diseases. Patients with cardiac channelopathies such as LQT3 and Brugada syndromes may also manifest sinus bradycardia.

**13.3.1.1.4. Other Bradycardias**

A sudden prolongation of the PP interval is common and occurs in almost half of all normal newborns and one-sixth of adolescents. These pauses are frequently related with an increased vagal tone^165^and some of them can be followed by supraventricular or ventricular escape beats.^168^

## 13.3.2. P wave and Atrial Electrical Activity

The characteristics of atrial activation remain relatively constant in all ages. P wave axis is very important to establish the rhythm site of origin, visceroatrial situs, and cardiac position.^170^The sinus P-wave axis is between 0 and +90 degrees. The normal P wave should not exceed 0.12 s and 2.5 mm (duration and amplitude); these parameters present few variations in different pediatric age groups ( Table 13.2 ).

### 13.3.2.1. Possible Alterations

**13.3.2.1.1. Atrial Hypertrophy**

P-wave amplitude is increased in right atrial hypertrophy, which is more clearly seen in D2 lead. Left atrial hypertrophy is characterized by an increase in P-wave duration (according to the age percentile) and/or its negative deflection in V1 (> 40 ms in duration and > 0.1 mV in amplitude).^168^

**13.3.2.1.2. Junctional Rhythm**

Junctional rhythm is characterized by a negative P wave in inferior leads (preceding, concomitant, or after the QRS complex). It can occur in up to one-third of normal children. It is more common during sleep, but it can happen during waking hours; in general, it does not have any pathological significance.

## 13.3.3. PR Interval and AV Conduction

### The PR interval increases with age, and presents an inverse relationship with HR. It varies according to autonomic tone ( Table 13.2 ).

## 13.3.3.1. Possible Alterations

**13.3.3.1.1. AV Blocks**

First-degree and type I second-degree AV block episodes occur in around 10% of normal children and up to 20% of normal adolescents. There are occasional periods of 2:1 AV block. They are more common during sleep, but can happen during waking hours, especially in individuals with vagotonia and in athletes.^165^

Type II and advanced second-degree AV block, as well as third-degree AV block, are usually pathological and can occur isolated or in association with complex cardiac malformations. The isolated form of congenital third-degree AV block affects 1:20 000 live births and is usually related with the transplacental passage of anti-Ro/SSA and anti-La/SSB antibodies.^168^

**13.3.3.1.2. Short PR Interval and Ventricular Pre-excitation**

A short PR interval can be detected in cases of junctional or other ectopic atrial rhythm. Pompe and Fabry diseases can also present it.^168 , 170^

Ventricular pre-excitation is characterized by a shortened PR interval with a delta wave.^170^Intermittent ventricular pre-excitation is not uncommon among newborns and children. Even when ventricular pre-excitation is persistent, the typical ECG pattern can be subtle. In these cases, the mid-precordial leads (V3-V4) can show them better. Wolf-Parkinson-White syndrome has an incidence of 0.15% to 0.3% in the pediatric population. An increased prevalence of ventricular pre-excitation is observed in individuals with hypertrophic cardiomyopathy, Ebstein anomaly, L-transposition of the great arteries, and cardiac tumors.

## 13.3.4. Ventricular Electrical Activity

The most prominent changes in ventricular electrical activity occur during a child’s first year of life. In the first days of life, the frontal plane QRS axis is directed downward and to the right, varying between 55º and 200º and reflects the predominance of the right ventricle over the left ventricle. It is less evident in tracings of preterm newborns, since fetuses with less than 32 weeks of gestational age have a larger left ventricle in comparison with the right ventricle. As the infant grows, the QRS axis deviates to the left, and when the baby reaches 6 months, it is at 65º.^166^On the transversal plane, QRS axis is directed rightwards and anterior at birth. Still in the first week of life, the QRS axis deviates to the left and maintains an anterior orientation, resulting in an increased R wave in V6 with persistent pure R waves in V1. On the transversal plane, the posterior QRS axis deviation is progressive. Thus, the R wave decreases slowly in V1 throughout the first year of life, even when exhibiting normal patterns in V5 and V6.^166^The morphology of QRS complexes in the precordial leads changes with the child development. This is explained by the modification of the ventricular electrical activation axis. The following aspects are present:

- The amplitude of the R wave in V1 increases during the first month of life and slowly decreases over the following several years. The amplitude, in this lead, should be < 18 mm in the first year of life and < 10 mm after this period;
- From birth to the sixth month of life, the R wave should be larger in V1 than in V6. R wave amplitude in V1 becomes virtually equal to that in V6 between 6 and 12 months of age. From this point on, it increases in V6 and progressively decreases in V1;
- Q waves are normal and can be very pronounced in the inferior and left precordial leads, and represent the septum activation, although they are absent in D1 and aVL. The Q waves amplitude varies with the child’s age and with the ECG lead. Its duration should not exceed 0.03 s ( Table 13.2 ).

In newborns, the QRS complex can be very narrow (in general, below 0.08 s). Its duration increases progressively with age, especially from the third year of life on ( Table 13.2 ).

### 13.3.4.1. Possible Alterations

**13.3.4.1.1. Changes in the QRS Axis and Amplitude**

A leftward axis deviation is present in many conditions, such as ventricular septal defects, tricuspid atresia, and WPW syndrome, but it can also be a normal variant. A rightward axis deviation can happen in Noonan syndrome, even in the absence of major pulmonary hypertension and in the presence of RVH.^170^

- RVH: should be suspected in the presence of a positive T wave in V1 after the first week of life and increased amplitudes of the R wave in V1 and S wave in V6. The QR pattern in V1 is usually seen in cases of pressure overload, and the rSR’ is seen in cases of right ventricular volume overload;^168^
- LVH: the ECG has limited accuracy for detecting LVH in children. The signs that help the most when diagnosing LVH are an increased S wave in V1, increased R wave amplitude in V6, and T wave abnormalities in V5 and V6;^168^
- Biventricular hypertrophy: wide and isodiphasic complexes in the mid-precordial leads — the Katz-Wachtel phenomenon. The R + S waves sum > 60 mm in V4 is very specific and can occur, for example, in cases of large ventricular septal defects.^170^

**13.3.4.1.2. Q-wave Alterations**

Pathological Q waves can be seen in children with anomalous coronary artery, ventricular pre-excitation, myocarditis, cardiomyopathy, and muscular dystrophies;^175^they are frequent in patients with hypertrophic cardiomyopathy, especially in the anterolateral leads (V4 to V6, D1 and aVL). In general, these are associated with signs of ventricular hypertrophy and ST-segment and T wave alterations.^176^It should be noted that the presence of a Q wave in V1 is always pathological.

**13.3.4.1.3. Intraventricular Conduction Disturbances**

Bundle-branch blocks diagnosis is determined by QRS duration and the patient’s age ( Table 13.2 ). RBBB can occur in some heart diseases such as Ebstein anomaly and after corrective surgery for congenital malformations such as Fallot tetralogy and interventricular communication. Isolated congenital forms of RBBB or LBBB are rare. Tricuspid atresia, ostium primum interatrial communication, anomalous coronary artery, and AV septal defects may be associated with left anterior fascicular block. LBBB is less frequent in children. The presence of LBBB in patients with severe cardiomyopathy is due to significant left ventricle/conduction system impairment. LBBB is generally associated with a poor prognosis.^168 , 170^

**13.3.4.1.4. Epsilon Wave and Arrhythmogenic Right Ventricular Cardiomyopathy (Dysplasia)**

See item 10.1.2.1.

## 13.3.5. Ventricular Repolarization

Ventricular repolarization is evaluated by measurement of the QT interval and the analysis of ST-segment morphology, T wave, and U wave in different leads.^168^

### 13.3.5.1. QT Interval

The QT interval presents an inverse relationship with the heart rate — the higher the HR, the shorter the QT interval, and vice-versa. In children, some peculiarities should be analyzed:^168^

- The QT interval should be measured in D2, V5, and V6 – use the longest one for calculating QTc;
- In higher heart rates, the P wave may overlap with the T wave and make QT measurement more difficult, especially in case of prolonged P wave;
- The U wave can be very prominent in children and should not be included in the QT interval if well separated from the T wave. In case of fusion between T and U waves, or when the U wave is too wide (> 50% of the T wave), the tangent method should be used;
- In cases of sinus arrhythmia, QTc should be calculated through the mean value of measurements obtained in various cardiac cycles;
- At 4 days old, children of both sexes have a mean QTc of 400 ± 20 ms. Around 2 months of life, there is a physiological prolongation of the QTc (mean value 410 ms); it then decreases progressively until 6 months of life, when it returns to the values recorded in the first week;
- In children, a normal QTc is up to 440 ms (97.5th percentile).^168^
- Although its routine use in pediatric cardiovascular screening is still under debate, the ECG has a crucial role in the early diagnosis of life-threatening arrhythmic heart diseases manifested during childhood and adolescence, mainly long QT syndrome (see below).

**13.3.5.1.1. Possible Alterations**

**13.3.5.1.1.1. Long QT Syndrome**

Long QT syndrome manifests predominantly during childhood and adolescence — few patients have symptoms during the first year of life.^177^Sudden death is the initial presentation of long QT syndrome in up to 12% of the cases.^177^Although this disease is relatively rare, the effort employed in its screening is justified by the efficacy of early treatment and prevention of sudden death.^168^Differential diagnoses should include secondary causes of QTc prolongation — see Section 11 for more details. During the first months of life, the infants of mothers with autoimmune diseases that express anti-Ro/SSA antibodies can present a very prolonged QTc, which is normally a transient finding that normalizes around the sixth month of life.^168^

**13.3.5.1.1.2. Short QT Syndrome**

See item 10.1.1.2.

### 13.3.5.2. ST-Segment

ST-segment should always be evaluated and the J-point position is fundamental to identify ST-segment elevation or depression. Thus, J-point position should be compared to the isoelectric line, which is usually at the level of the PQ segment. In newborns and infants, the TP segment (isoelectric line between the T wave and the following P wave) is more commonly used as reference for the baseline.^164^

**13.3.5.2.1. Possible Alterations**

**13.3.5.2.1.1. ST-Segment Deviations**

Slight ST-segment deviations are common during the first month of life (usually < 2 mm). ST-segment elevation up to 3 mm can be seen in the right precordial leads and is considered a normal finding (specially from 1 year of age).^168^Ventricular hypertrophy, cardiomyopathies, pericarditis, ventricular pre-excitation, anomalous coronary artery origin, and medications, among other factors, may alter ventricular repolarization and lead to ST-segment elevation or depression. Despite its low sensitivity, ST-segment depression presents good specificity for diagnosing ventricular hypertrophy. Cases of anomalous origin of the left coronary branch (arising from the pulmonary artery) are manifested as an extensive anterior infarction (usually after the first month of life).^170^

**13.3.5.2.1.2. Early Repolarization**

See item 9.1.2.1.

**13.3.5.2.1.3. Brugada Electrocardiographic Pattern**

Brugada pattern is rare in children and it has a much lower frequency than among adults.^177^For more details, see item 10.1.1.3.

### 13.3.5.3. T Wave

During birth, positive T waves in the right precordial leads are normal and are probably due to the physiological adaptation of the right ventricle to new hemodynamic characteristics and lower myocardial elasticity. In normal children, after the second or third day of life, the T wave vector is deviated posteriorly and to the left. Thus, the T wave becomes negative in V1 at the end of the first week. From 7 days to 7 years of life, positive T waves in V1 are usually associated with RVH.^170^The T wave can remain negative from V1 to V4 (juvenile T-wave pattern) until age 12–14 years, when it becomes positive from V2 to V6. The presence of negative T waves in these leads after this period can be considered a normal variant in 1–3% of the cases, but they must be investigated.^170 , 178 , 179^Pericarditis, myocarditis, cardiomyopathies, myocardial ischemia, ventricular hypertrophy, and hydroelectrolytic imbalance may also lead to changes in the T wave. Symmetrical, negative, and wide T waves are not uncommon in the precordial leads in patients with hypertrophic cardiomyopathy. In cases of children with severe acute brain injury large negative T waves in various leads can be seen. This is known as “cerebral T waves” (Item 11.1.5).

### 13.3.5.4. U Wave

U Wave is not always visible on the ECG, but it can be prominent in children, in cases of hypokalemia, with antiarrhythmic drugs, and long QT syndrome.

## 13.4. Heart Rhythm Disorders

The electrocardiographic criteria used for assessing cardiac arrhythmias in children follow those used for adults (see Section 3).

## 13.5. Identification of Situs, Cardiac Position, and Ventricular Inversion

The identification of *situs* through the ECG is fundamentally based on the orientation of the P wave, which is positive in D1 and V6 in *situs solitus* and negative in *situs inversus* .^166^In this case, electrode reversal and left atrial rhythm are the main differential diagnoses.

In patients with *situs solitus* and levocardia, the P wave axis and the QRS axis are in the left lower quadrant (frontal plane). In *situs inversus* with dextrocardia, the P wave axis and the QRS axis are in the right lower quadrant. The P wave axis and the QRS axis are located in different quadrants when there is a disagreement between *situs* and cardiac position, such as in dextrocardia with *situs solitus* (which is usually associated with complex congenital heart diseases).^170^

The orientation of the first (5–20 ms) QRS vectors is important for determining the position of the ventricles. In case of ventricular inversion, the first vectors are directed towards the left and Q waves are not seen in D1 and V6.^166^

## 14. The ECG during Cardiac Pacing

### 14.1. Cardiac Pacing

Basically, the ECG of an individual with a cardiac implantable electronic device (CIED) is characterized by the presence or absence of spikes (artifacts resulting from pulse energy emission by pacing).

Except for implantable monitors (loop recorders), all other CIED (pacemakers, cardiac resynchronization devices, and implantable cardioverter-defibrillators – Table 14.1 ) are capable of emitting spikes, especially for treating bradycardias. So, the identification of the type of CIED based on ECG interpretation frequently is not possible. On the other hand, proper device functioning, as well as dysfunctions, can be recognized on the ECG. Pacing configuration, unipolar or bipolar, determines the size of the spike. In unipolar pacing, potential difference is established between the pulse generator and the lead tip, which potential difference results in a large amplitude vector. Consequently, the spikes should have large amplitudes too. In bipolar pacing, potential difference is established between the anode and cathode on the lead tip; the vector generated by the potential difference should thus be small and the spikes recorded in this mode should also be small (sometimes almost imperceptible).

**Table 14.1:** Types of CIED and their classical indications.

CIED	Basic properties	Main indication
**Conventional pacemaker**	Atrial and/or ventricular pacing	Bradiarrhythmias
**Cardiac resynchronization device**	Atriobiventricular pacing	Refractory heart failure with LBBB
**Implantable cardioverter-defibrillator**	Atrial and/or ventricular pacing and therapies for ventricular tachyarrhythmia	Prevention of sudden cardiac death
**Implantable cardioverter-defibrillator and cardiac resynchronization device**	Atriobiventricular pacing Anti-ventricular tachyarrhythmia therapies	Refractory heart failure with LBBB Prevention of sudden cardiac death

CIED: cardiac implantable electronic devices.

The terms and codes (5-letter code – Table 14.2 ) used for describing CIED properties follow an international standard by the North American Society of Pacing and Electrophysiology (Naspe) and the British Pacing and Electrophysiology Group (BPEG).^180^ Figure 14.1 illustrates the algorithm for identifying the mode of operation of CIED.

**Table 14.2:** 5-letter code for electrocardiographic identification of the mode of operation of CIED

I Chambers paced	II Chambers sensed	III Response to sensing	IV Rate modulation	IV Multisite pacing
O: none	O: none	O: none	O: none	O: none
V: ventricle	V: ventricle	T: trigger		A: atrium
A: atrium	A: atrium	I: Inhibited		V: ventricle
D: dual (A+V)	D: dual (A+V)	D: dual (A+V)		D: dual (A+V)
S: Single-chamber (A or V)	S: Single-chamber (A or V)		R: rate modulation	

**Figure 14.1:** Algorithm for interpretation of ECG of individuals with CIED. 181

#### 14.1.1. Basic Terms

- Spike — corresponds to the electrical impulse emitted by the CIED;
- Capture — artificial tissue depolarization after the spike;
- Basic rate — atrial/ventricular pacing rate without interference of spontaneous beats;
- AV interval (AVI) — interval between spontaneous (sensed) or paced (spike) atrial activity and ventricular pacing;
- Interventricular interval (IVI) — interval between 2 ventricular spikes, programmable by telemetry, available in cardiac resynchronization devices, and that can be sometimes identified on a resting ECG;
- Maximum rate limit (MRL) — maximum pacing rate. In single-chamber devices, the maximum rate is reached with the activation of an adaptive rate pacing sensor. In dual-chamber devices, the maximum rate is reached in response to atrial sensitivity (frequency of P waves) or also by rate sensor activation;
- Sensitivity — ability to recognize atrial (P waves) or ventricular (QRS complex) spontaneous electrical events;
- Normal inhibition — the pacing activity is inhibited by the intrinsic rhythm (no spikes).

#### 14.1.2. Analysis of the Electrocardiographic Characteristics of CIED

- Normal device operation — when normal capture and sensitivity are observed;
- Loss of atrial and/or ventricular capture (intermittent or persistent) — absence of depolarization in the paced chamber (presence of a spike, but without triggering a P wave or QRS complex);
- Sensitivity failure:
  c.1) Excessive sensitivity (oversensing) — inappropriate sensitivity, resulting in the misidentification of spurious electrical signal that does not correspond to depolarization inhibiting spikes emission (electromagnetic interference, myopotentials, T wave, etc.);
  c.2) Reduced sensitivity (undersensing) — inability to recognize spontaneous depolarization. It may occur due to inadequate programming or due to changes in the intrinsic signal (the system does not “see” the P wave or QRS complex).
- Fusion beats — correspond to the artificial activation of cardiac tissue simultaneously to spontaneous depolarization, resulting in hybrid complexes. The pacemaker spike is followed by a P wave (atrial fusion) or a QRS complex (ventricular fusion), which morphology is in between capture and spontaneous beats;
- Pseudofusion beats — spontaneous activation of cardiac tissue, concurrent with the device spike, which has no effect on the P wave or QRS complex (ventricular and atrial pseudofusion, respectively). The morphology of the wave following the spike is similar to that of the spontaneous beat;
- Pacemaker-mediated tachycardia — restricted to AV CIED and characterized by ventricular triggering from a retrograde P wave. Therefore, this arrhythmia is caused by a circular movement in which the artificial pacing is the anterograde component of the circuit and the retrograde component is anatomic (normal or anomalous pathway);
- Pacemaker-conducted tachycardia — tachyarrhythmia also involving AV CIED, characterized by the presence of supraventricular arrhythmia that, when perceived by the atrial channel, triggers ventricular capture at high rate, maintaining some characteristics of spontaneous arrhythmia;
- Pacemaker-induced tachycardia — changes in sensitivity or electromagnetic interference that cause atrial or ventricular arrhythmias caused by inadequate pacing.

## 15. Tele-electrocardiography

Telemedicine is the remote delivery of health care services with the use of information and communication technologies in situations where one (or two) health care professionals and the patient are not physically present.^182^Tele-ECG systems record the electrocardiographic tracing obtained at a distance through different means and data transfer technologies. A remote physician analyzes and interprets the electrocardiographic tracing, and the report is made and sent electronically. Tele-ECG is related to ECG development. In 1905, Einthoven described the transtelephonic transmission of the ECG from the university hospital to the physiology laboratory at the University of Leiden, located 1.5 km away.^183^

With the advent of the computerized ECG^184^and systems capable of transmitting electrocardiographic tracings via the internet, the ECG with the specialist report became available in locations far from large counties centers. Tele-ECG units began to be implemented in Brazil in the 2000s, improving access to the electrocardiographic diagnosis and early recognition of relevant electrocardiographic abnormalities that could be fatal.^185^

Specific infrastructure is required to implement and run a tele-ECG department ( Table 15.1 ). The central ECG interpretation unit should have a team of cardiologists, information technology (IT) specialists, and administrative assistants. Complete IT infrastructure with computers, hardware, software, and data protection and storage systems are vital for proper functioning. The remote health care units that perform the ECG should have a digital electrocardiograph (approved by the corresponding federal agencies), internet connection, equipment, and services that allow audio or video communication, in addition to training all professionals involved.^182^We recommend that the original electrocardiographic signal be transmitted from images generated by the electrocardiograph itself or by professional scanners, avoiding low-quality digitization or image distortions that could hinder or prevent the ECG analysis.^182^

**Table 15.1:** Technical requirements for implementing tele-ECG

TECHNICAL STANDARDS
ANVISA registration
ABNT NBR IEC 60601-1 (general safety standard)
ABNT NBR IEC 60601-1-1 (safety of electromedical systems)
ABNT NBR IEC 60601-1-2 (electromagnetic compatibility)
ABNT NBR IEC 60601-1-4 (programmable electromedical systems)
ABNT NBR IEC 60601-2-25 (safety of electrocardiographs)
ABNT NBR IEC 60601-2-251 (safety standard, including the essential performance of electrocardiographs, single- and multi-channel recorders and analyzers)
**MINIMUM EQUIPMENT REQUIREMENTS**
Desktop or laptop computer
At least 1 USB port (2.0 or 3.0)
CD/DVD drive
4 GB memory
Intel Pentium® processor
Windows 7, 8, or 10
250GB hard drive or more
**RECOMMENDATIONS**
12 leads
High-quality tracing (1200 samples/second/channel)

Tele-ECG has been shown to be an effective strategy in the rationalization of access to complementary propaedeutics, early diagnostics, prioritization of referrals, and organization of waitlists in health care systems, improving cost-effectiveness and health care assistance ( Table 15.2 ).^187^Especially in rural areas, the prehospital tele-ECG performed in patients with acute coronary syndrome reduced door-to-balloon time and long-term mortality.^188^The detection of AF^190^and some channelopathies such as Brugada syndrome was also improved.^191^Moreover, the use of tele-ECG databases is essential for nationwide epidemiological studies.^192^

**Table 15.2:** Benefits of the tele-ECG ^187^

Quick electrocardiographic diagnosis, enabling the identification of normal and abnormal cases
Prehospital care at the patient’s location
Access to specialists in case of accidents and emergencies
Reductions in time and costs for the patient
Faster triage by specialists
Help and guidance to non-specialists
Easy management of health care resources
Increased safety of postoperative patients during rehabilitation
Cooperation and integration between researchers for sharing clinical records
Access to educational programs of training and qualification
A second opinion

The constant growth of healthcare-related technology extended new perspectives in tele-ECG. The use of artificial intelligence (AI) techniques in ECG is increasing exponentially, with good results in the automatic diagnosis of electrocardiographic abnormalities,^193^and in the development of new cardiovascular risk markers.^195^The wearable equipment such as the chest strap HR monitor, adhesive ECG patches, smartphones, and smartwatches allowed the early identification of possible cardiac arrhythmias, especially AF.^196^These portable and easy-to-use devices allowed fast HR recording in the patient’s daily life, in any environment and at any time, followed by automatic and real-time interpretation via AI. A significant limitation of this technology still is the cost. As a consequence of modern techniques, we cannot ignore a possible increase in workload (recording and sending information from patients to their physicians), false-positive cases resulting from artifacts, and the increased emotional burden to some patients when “discovering” a cardiac arrhythmia. The following years should elucidate the role of new methodologies and technologies in clinical practice. However, with these advancements, we believe the ECG can reach new uses and applications.
